# Synthesis and Transformations of NH‐Sulfoximines

**DOI:** 10.1002/chem.202102619

**Published:** 2021-10-13

**Authors:** Michael Andresini, Arianna Tota, Leonardo Degennaro, James A. Bull, Renzo Luisi

**Affiliations:** ^1^ Department of Pharmacy-Drug Sciences University of Bari “A. Moro” Via E. Orabona 4 70125 Bari Italy; ^2^ Department of Chemistry Imperial College London Molecular Sciences Research Hub White City Campus, Wood Lane London W12 0BZ UK

**Keywords:** hypervalent iodine, nitrogen transfer, organic synthesis, organosulfur, sulfoximines

## Abstract

Recent years have seen a marked increase in the occurrence of sulfoximines in the chemical sciences, often presented as valuable motifs for medicinal chemistry. This has been prompted by both pioneering works taking sulfoximine containing compounds into clinical trials and the concurrent development of powerful synthetic methods. This review covers recent developments in the synthesis of sulfoximines concentrating on developments since 2015. This includes extensive developments in both S−N and S−C bond formations. Flow chemistry processes for sulfoximine synthesis are also covered. Finally, subsequent transformations of sulfoximines, particularly in N‐functionalization are reviewed, including N−S, N−P, N−C bond forming processes and cyclization reactions.

## Graphical abstract

**This review** considered recent progress and achievements in the synthesis and transformation of NH‐sulfoximines. The development of strategies for direct and selective preparation of NH‐sulfoximines, even in enantioenriched form or by using sustainable approaches and continuous flow synthesis, are reported. Transformations of sulfoximines by N−S, N−P, N−C bond forming processes and cyclization reactions are also covered.

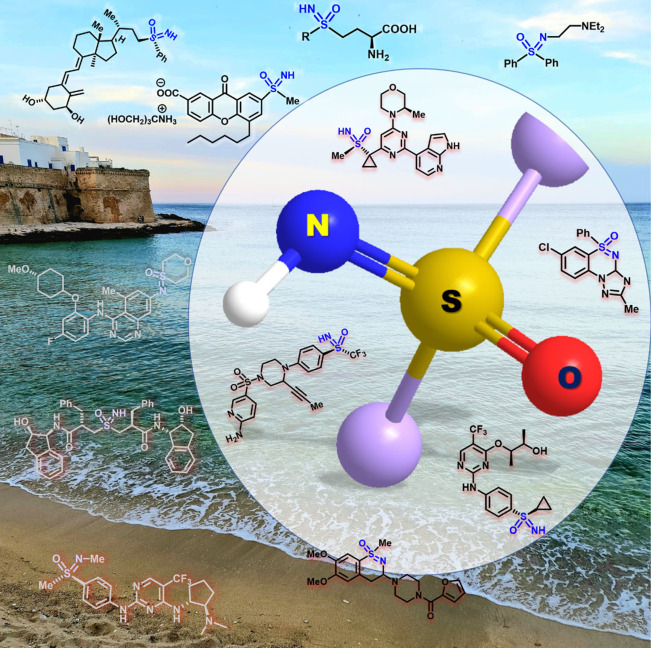

## 1 Introduction

**Scheme 1:**
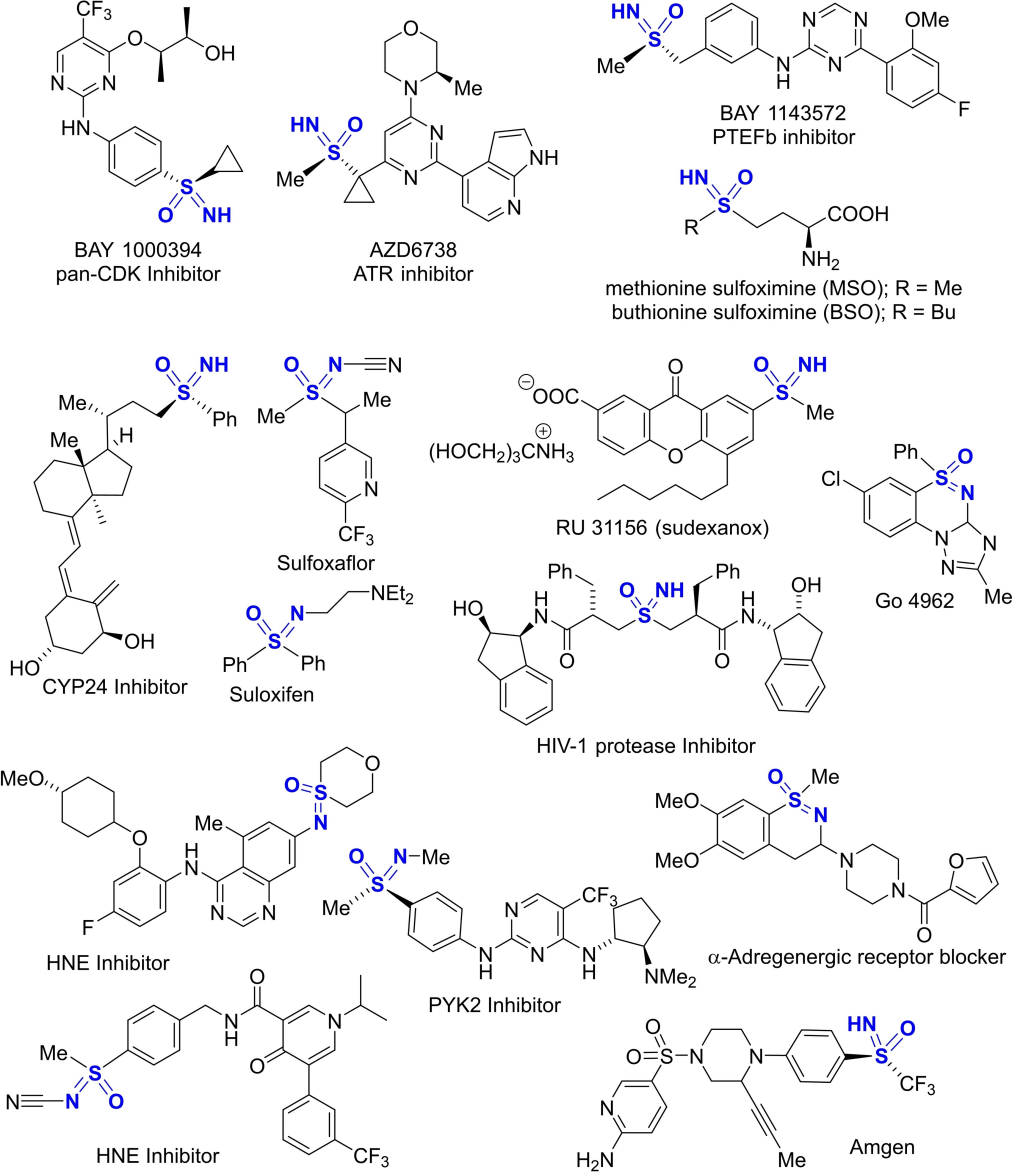
Biologically relevant sulfoximines.

Sulfoximines, the mono‐aza analogues of sulfones, have attracted the interest of numerous research groups worldwide, as witnessed by the large number of publications appeared in the last decade.[^1^, ^2^] Since the first discovery of the irreversible glutamine synthetase inhibitor L‐methionine‐(S)‐sulfoximine (MSO),[^3^, ^4^, ^5^] the number of bioactive molecules including the sulfoximine moiety in their structure increased dramatically.^[6]^ Soon after, buthionine sulfoximine (BSO), a gamma‐glutamylcysteine synthetase inhibitor, was found suitable for treating tumors in which GSH is overexpressed, and as adjuvant in chemotherapy.^[7]^ A wide range of sulfoximines have been assessed as bioactive agents and some entered clinical trials, as in the case of the kinase inhibitors roniciclib,^[8]^ BAY 1143572,^[9]^ and AZD 6738,^[10]^ for the treatment of cancer (Scheme 1). Very recently a new sulfoximine forming compound was reported to treat herpes infections.^[11]^

From a structural point of view, sulfoximines feature a tetrahedral sulfur atom, and a basic nitrogen atom able to coordinate metal ions and form salts with mineral acids.^[12]^ The stereogenicity of the sulfur center provides configurationally stable and hence optically active sulfoximine stereoisomers. The sulfoximine moiety can introduce favorable pharmacokinetic properties to molecular scaffolds such as better solubility in protic solvents, hydrogen‐bond acceptor/donor capability, and chemical and metabolic stability in comparison to related sulfone or sulfonamide structures.[^6^, ^13^, ^14^] These physicochemical properties can be additionally tuned by N‐functionalization reactions. In addition to the great interest in the chemistry of sulfoximines in drug discovery programs, this S(VI) functionality finds use in modern synthesis as chiral auxiliaries or ligands for asymmetric catalysis.[^15^, ^16^] In addition, possible degradation and conversion pathways for sulfoximines have been investigated, in order to assess the potential risk of sulfoximine metabolites for crop protection and medicinal chemistry applications.^[17]^ The renewed interest in the chemistry of sulfoximines, is showcased by the invention of new synthetic strategies for their preparation and functionalization.[^18^, ^19^]

This review aims to provide an up‐to‐date overview of the recently introduced synthetic strategies for accessing *NH*‐sulfoximines, and to also cover their functionalization. The field continues to expand rapidly, and the review will concentrate on recent advances from the last decade, and particularly since a major review by Bolm.^[19]^ We will first focus on methods that directly form NH‐sulfoximines (rather than via an intermediate protected form). We also cover those applications in continuous flow. Then we review methods for the functionalization of these NH derivatives, separated by the nature of the N‐functional group. Finally, we cover cyclisation reactions for the formation of non‐planar heterocycles containing the S(O)=N functionality. Together, we expect this will provide a valuable reference for the synthetic and medicinal chemistry communities for the preparation of these valuable motifs and their derivatives.

## 2 Synthesis of NH‐Sulfoximines

**Scheme 2:**
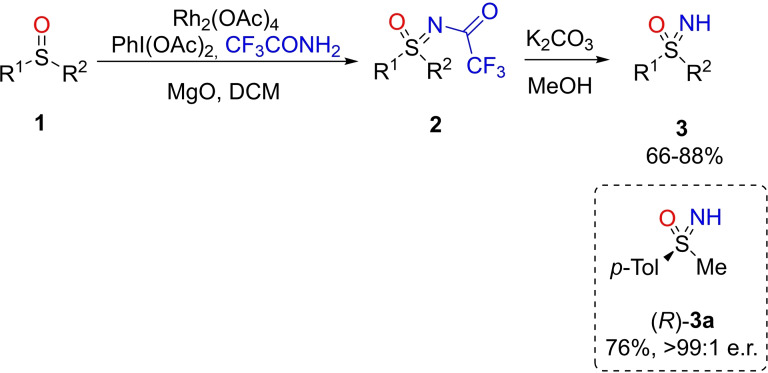
Rhodium‐catalyzed imination of sulfoxides and deprotection reaction.

The most classical routes to access sulfoximines involve the initial introduction of nitrogen or oxygen to sulfides to give, respectively, the corresponding sulfilimines or sulfoxides. Further oxidation of sulfilimines or N‐transfer to sulfoxides provide the corresponding sulfoximines. These simple routes commonly provide N‐protected sulfoximines which require a final deprotection step for the formation of NH‐sulfoximines. The N‐transfer steps have been carried out through metal‐catalyzed or metal‐free processes. In 2004, Bolm and Okamura described an efficient two‐step method for accessing NH‐sulfoximines from sulfoxides.^[20]^ This protocol achieved the synthesis of N‐trifluoroacetylsulfoximines **2** by reacting trifluoroacetamide with iodobenzene diacetate and magnesium oxide with Rh a catalyst (Scheme 2). The resulting N‐acyl sulfoximines were readily deprotected with potassium carbonate in methanol affording NH‐sulfoximines **3** in good yields (Scheme 2). Notably, an air‐stable rhodium catalyst and a mild oxidant is involved, avoiding the use of hazardous iminating agents such as azido derivatives or the explosive *O‐*(mesitylenesulfonyl)hydroxylamine (MSH).^[21]^ The reaction of an optically pure sulfoxide allowed the preparation of the corresponding enantiopure NH‐sulfoximine (*R*)‐**3** **a** (>99 : 1 er) without any loss of optical purity. Under these conditions, the imination reaction was stereospecific and occurred with retention of configuration at the sulfur center.

**Scheme 3:**
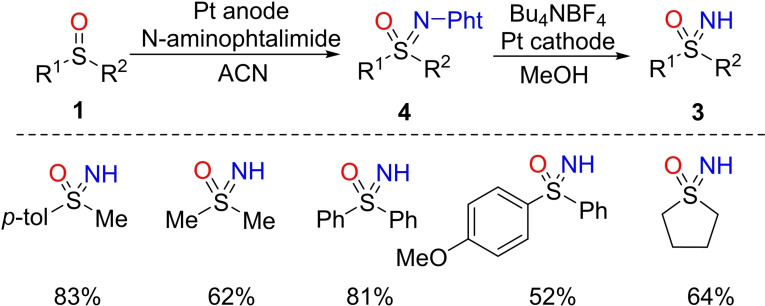
Electrochemical imination of sulfoxides.

NH‐Sulfoximines are also accessible through the electrolysis of N‐phtalimido sulfoximines **4** in methanol, using water as the proton source, under electrochemical conditions.^[22]^ The protocol, developed by Yudin and Siu, enabled the preparation of several dialkyl and diarylsulfoximines **3** in good yields (Scheme 3). The authors reported the complete conversion of the starting materials, and the strategy avoids metal‐based reagents, catalysts, and toxic oxidants.

**Scheme 4:**
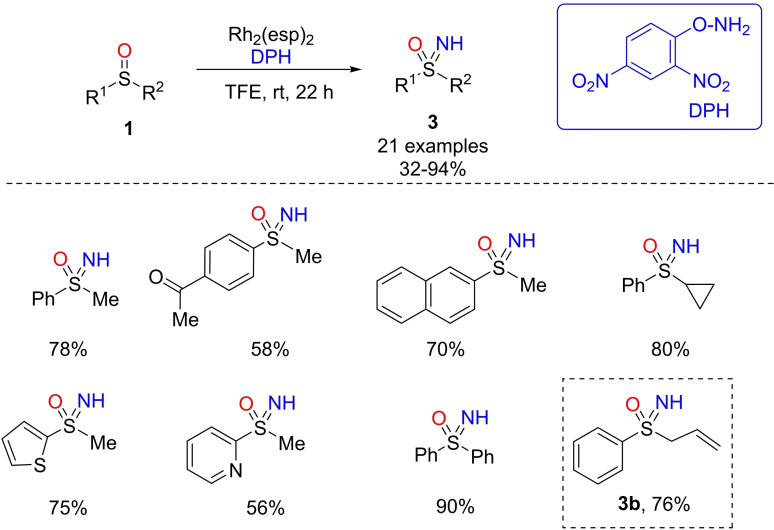
Direct Rh‐catalyzed synthesis of NH‐sulfoximines from sulfoxides.

More recently, novel strategies involving NH‐transfer or the simultaneous one‐pot NH‐ transfer or NH‐ and O‐transfer starting from sulfoxides or sulfides have been introduced, allowing direct access to NH‐sulfoximines without any further deprotection step. Inspired by the recent advances by Falck, Kurti and coworkers in the direct synthesis of NH‐aziridines from olefins,^[23]^ Richards and Ge developed the first rhodium‐catalyzed strategy for the preparation to NH‐sulfoximines directly from sulfoxides.^[24]^ The optimized protocol required 3 equivalents of O‐(2,4‐dinitrophenyl)‐hydroxylamine (DPH) and 2.5 mol% of Rh_2_(esp)_2_ in trifluoroethanol (TFE), to obtain NH‐sulfoximines **3** in moderate to excellent yields (Scheme 4). The scope of the reaction was broadly explored, as well as the compatibility of some functional groups such as halogens, and acyl groups on the phenyl ring of the starting sulfoxide. Diaryl, dialkyl, and cycloalkyl sulfoximines **3** were prepared with very good yields, and heteroaryl 2‐thiophenyl, 2‐pyridyl sulfoxides were likewise transformed. Moreover, the authors investigated the chemoselectivity of the reaction by reacting phenyl allyl sulfoxide. In this case, the imination reaction was found to be favored over aziridination providing sulfoximine **3** **b** in 76 % yield. Concerning the mechanism of this N‐transfer strategy, the authors proposed the generation of a reactive Rh‐nitrene intermediate, by the reaction of DPH with Rh_2_(esp)_2_ and subsequent loss of dinitrophenol.

**Scheme 5:**
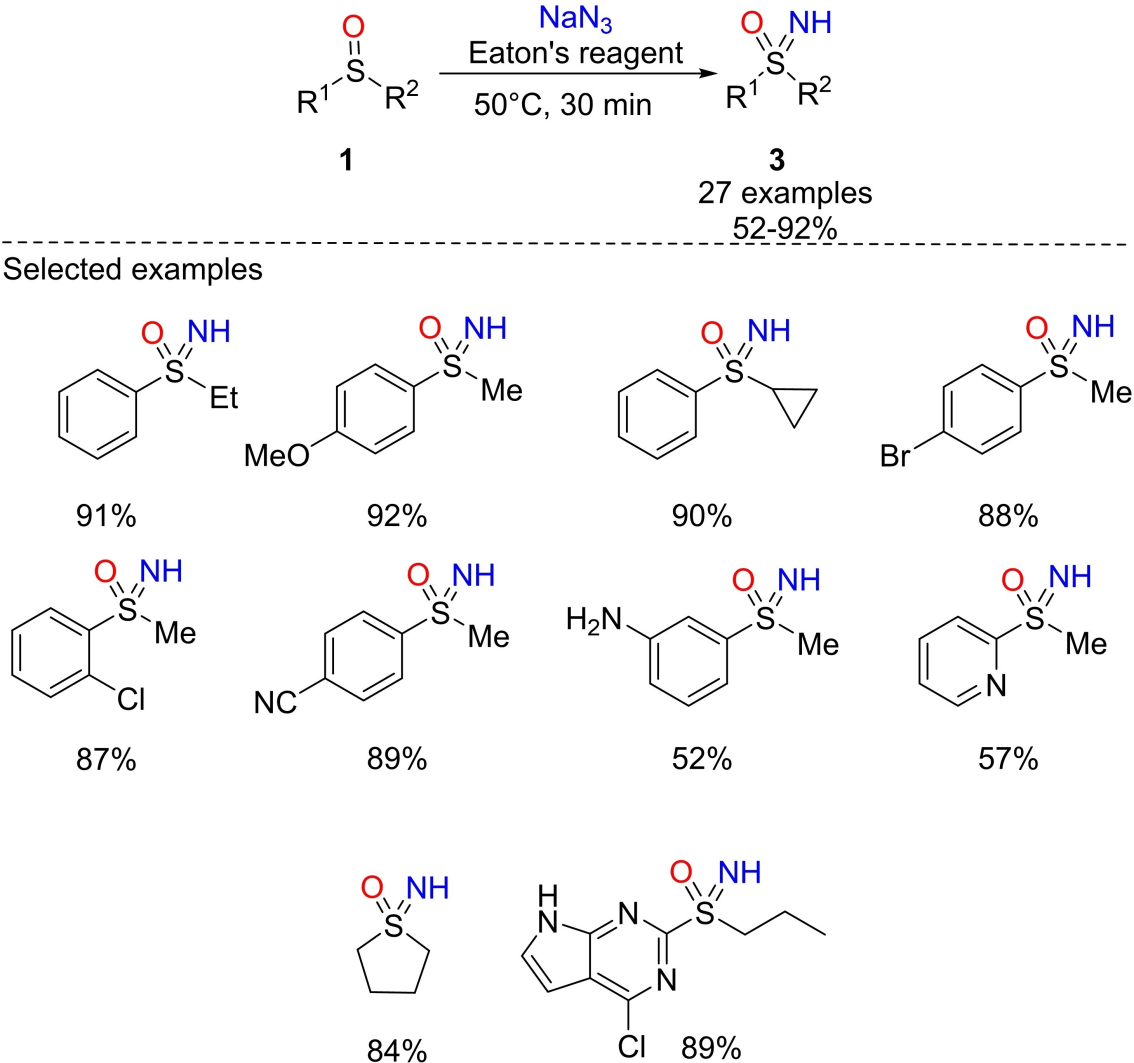
Sulfoxides reaction with Eaton's reagent towards NH‐sulfoximines.

In 2017, Liang reported the preparation of NH‐sulfoximines **3** from sulfoxides **1** using NaN_3_ and Eaton's reagent (P_2_O_5_ in methanesulfonic acid) at 50 °C (Scheme 5).[^25^, ^26^] Very good yields of the corresponding NH‐sulfoximines were obtained employing, 2 equivalents of NaN_3_. Attempts to reduce the amount of Eaton's reagent by using co‐solvents of chloroform or THF, and alternatively by running the reaction in neat methanesulfonic acid, caused a decrease of yields. The reaction was found to be efficient using alkyl‐arylsulfoxides, and good tolerance was proved toward methoxy, cyano, halogens, and other substituents of the phenyl ring. Furthermore, this imination protocol was found to be efficient with aryl, heteroaryl and carbocyclic sulfoxides (Scheme 5). However, enantiopure sulfoxides returned a racemic mixture of the corresponding sulfoximines. The proposed mechanism involved an unstable electrophilic aminodiazonium ion H_2_N_3_^+^ able to provide the electrophilic nitrogen upon release of molecular N_2_.^[27]^ However, the role of P_2_O_5_ in promoting the imination reaction remains to be clarified.

**Scheme 6:**
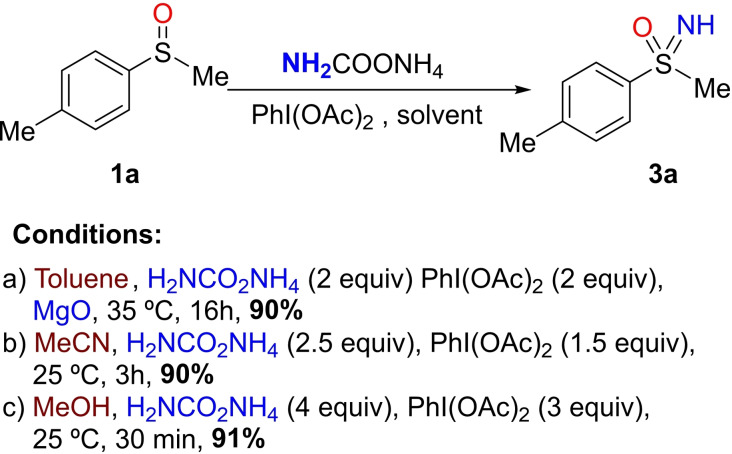
Optimized conditions for the NH‐transfer to sulfoxides.

In 2016, we (Luisi and Bull) developed a direct metal‐free method for *NH* transfer to sulfoxides using ammonium carbamate as inexpensive and easy to handle nitrogen source, in the presence of diacetoxyiodobenzene (DIB) as the oxidant.^[28]^ The reaction could be successfully conducted with different solvents under slightly different conditions (Scheme 6). The combination of ammonium carbamate and DIB in polar solvents, such as acetonitrile or methanol, as well as in nonpolar solvents such as toluene, provided excellent yields of the corresponding sulfoximine **3** **a** from sulfoxide **1** **a**. Interestingly, the method was readily scalable.

**Scheme 7:**
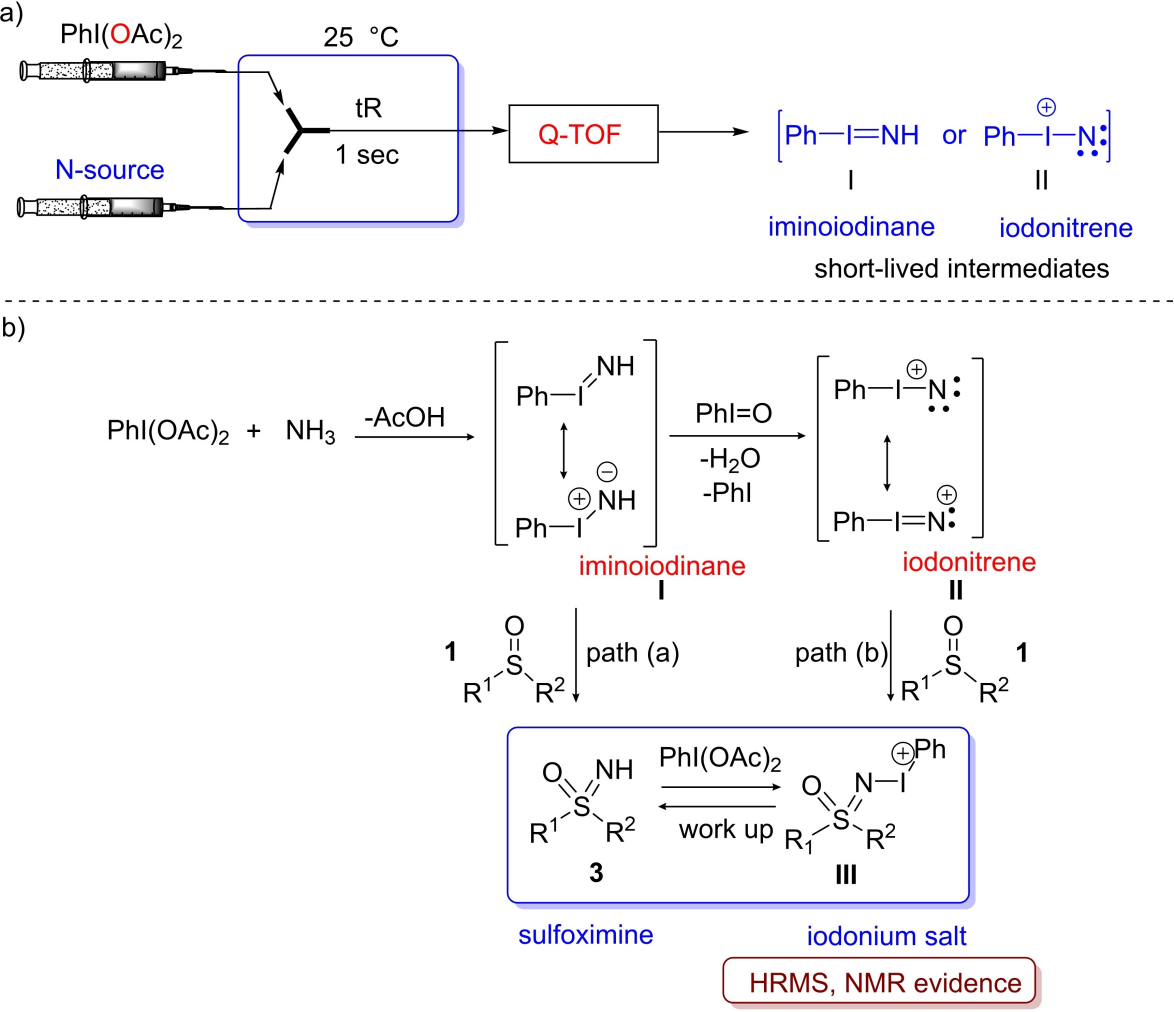
Proposed reaction mechanism for imination of sulfoxides.

The scope of the reaction was very general, working effectively with a wide range of sulfoxides, and the process proceeds with complete retention of configuration at the sulfur atom of enantioenriched sulfoxides. The functional group tolerance of the reaction was shown to be very high, and further demonstrated using Glorius’ robustness screen.^[29]^ Notably, heterocycles bearing basic nitrogen atoms (pyridine, pyrimidine, imidazole) were found to be highly compatible with the imination protocol, while electron‐rich heterocycles such as indole or furan were less tolerated. The mechanism of this NH‐transfer was thoroughly investigated, and we proposed an unprecedented iodonitrene or iminoiodinane as key electrophilic intermediates responsible for the N‐transfer to the sulfur atom.^[30]^ By using a continuous flow‐MS set‐up, mixing of PhI(OAc)_2_ and ammonium carbamate revealed the HRMS signals of the short‐lived iminoiodinane (PhI=NH) **I** and iodonitrene (PhI=N^+^) **II** (Scheme 7, a).^[31]^ Moreover, the use of ^15^N‐labeled ammonium acetate, as the N‐source, resulted into the generation of ^15^N‐labeled intermediates **I** and **II**. According to the mechanistic investigation, ammonia, deriving from ammonium carbamate, reacts with PhI(OAc)_2_ to generate the intermediates iminoiodinane **I** or iodonitrene **II** to react with the sulfoxide (Scheme 7, b). At the time, we proposed both of these as possible intermediates. The direct attack of the sulfoxide at iminoiodinane **I** would form NH‐sulfoximine **3** and iodobenzene.^[32]^ or iodonitrene **II** would furnish the iodonium salt **III**, which collapses towards NH‐sulfoximine **3** after work‐up. However, further developments of these reagents, suggest that the iodonitrene **II** is the true reagent, which is consistent with the direct and rapid formation of the iodonium salt **III** in situ.

**Scheme 8:**
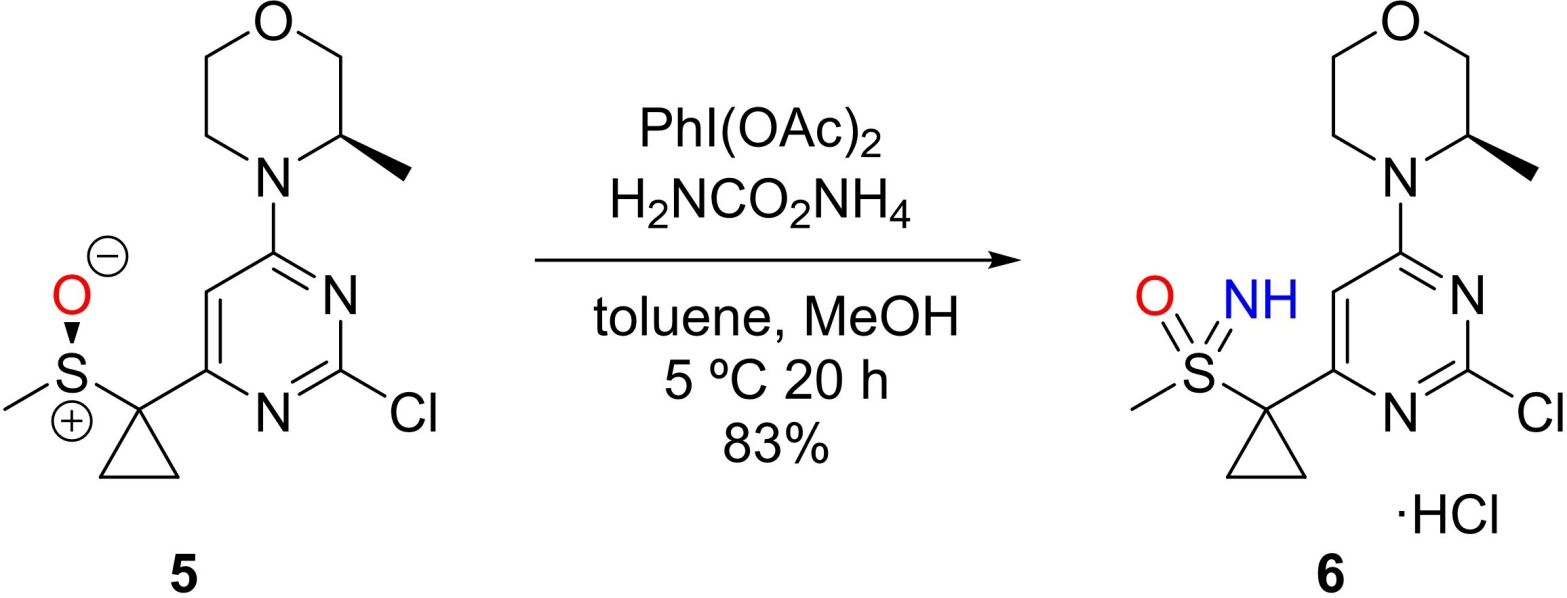
Manufacturing scale formation of Ceralasertib intermediate

This stereospecific NH transfer to sulfoxides, has been adopted into the manufacturing scale production of ATR Inhibitor AZD6738 (Ceralasertib) from AstraZeneca.[^33^, ^34^, ^35^, ^36^, ^37^] Graham et al. reported the preparation of the sulfoximine containing intermediate **6** from the corresponding sulfoxide **5** (Scheme 8).

The optimized conditions for this process used a reduced amount of PhI(OAc)_2_ (2.1 equivalents) at a reaction temperature of 5 °C in a mixed solvent system of MeOH and toluene. This enabled the preparation of 30 kg of the intermediate compound as the HCl salt at 99 % purity. This replaced the earlier development route which used Rh catalyzed NH transfer, using trifluoroacetamide with dichloromethane solvent.^[38]^

**Scheme 9:**
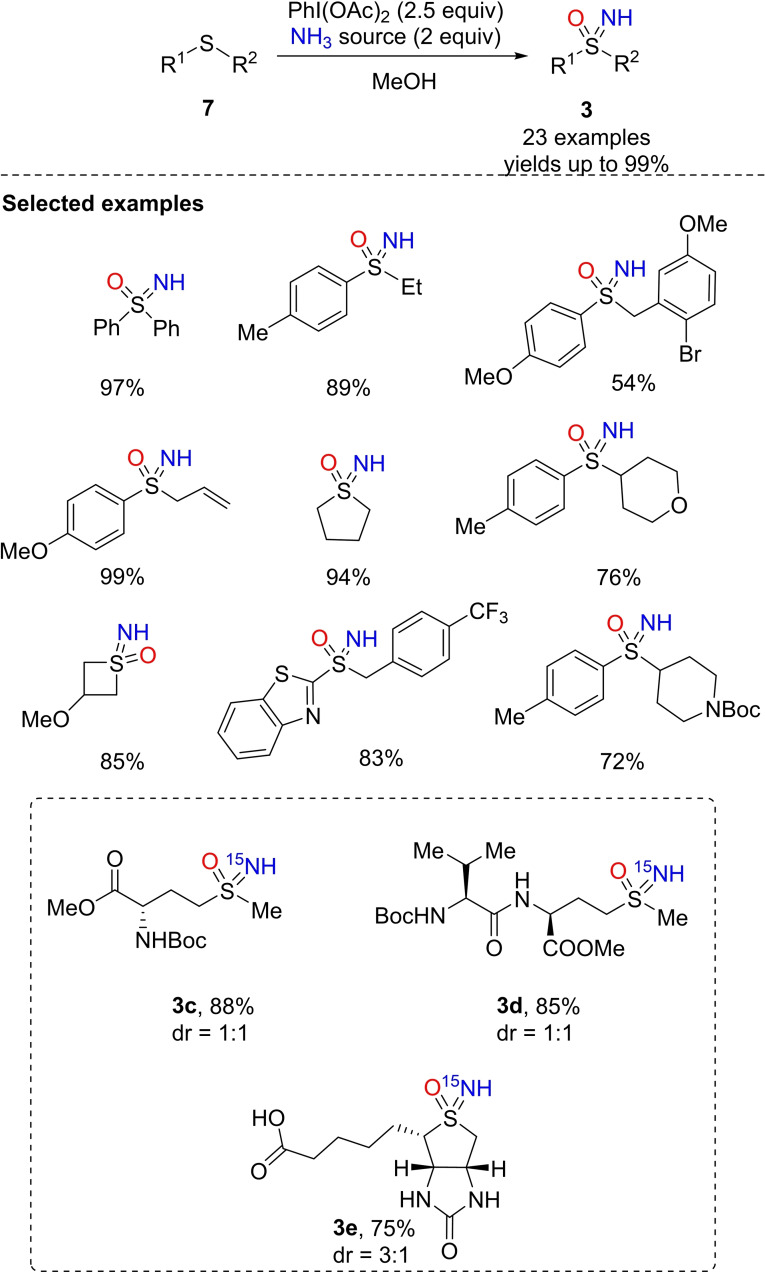
One‐pot NH and O transfer to sulfides.

**Scheme 10:**
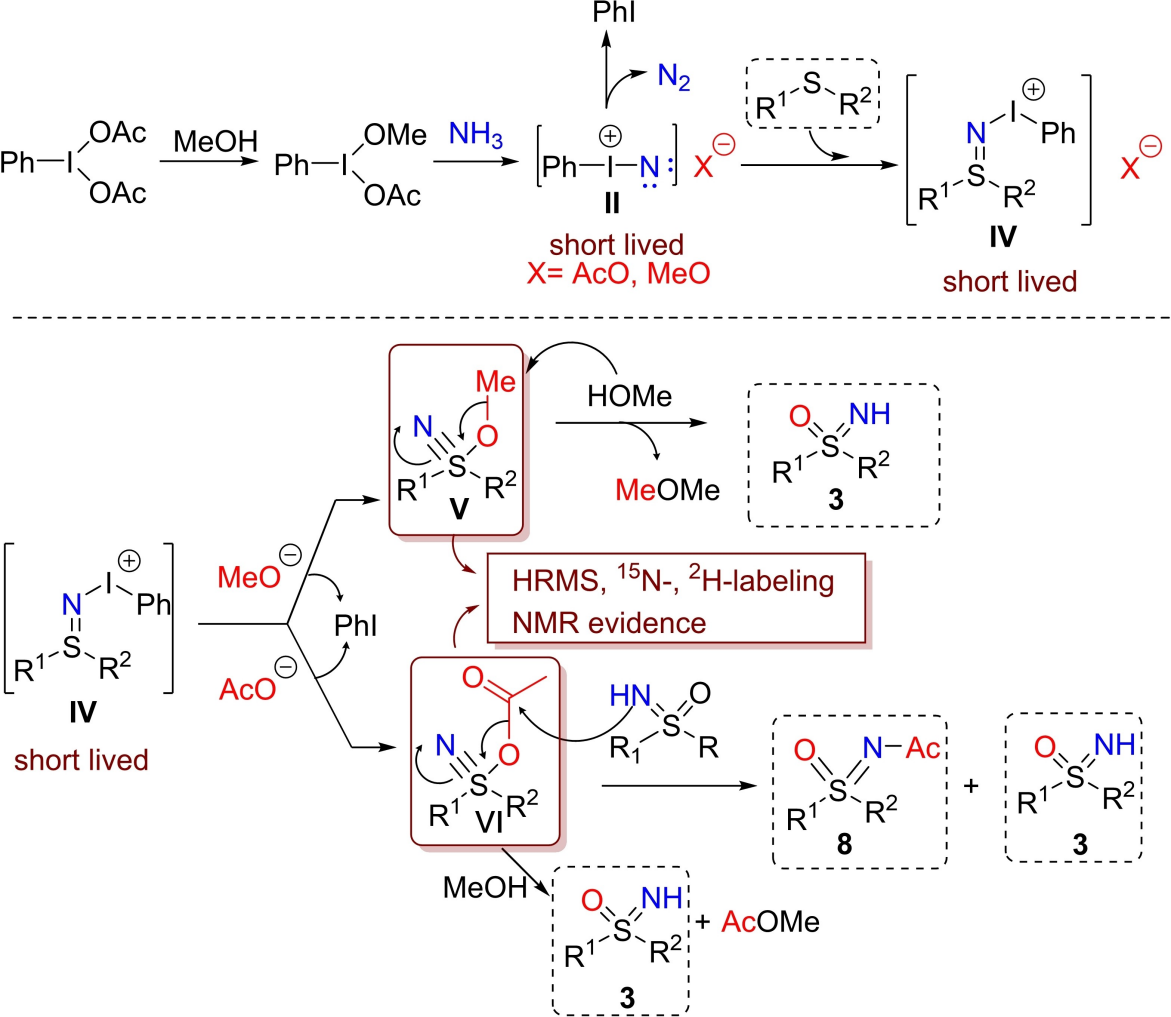
Mechanistic evidence for λ^6^‐sulfanenitrile intermediates.

Next, Luisi and Bull reported that the combination of a source of ammonia and hypervalent iodine oxidant (DIB) was effective for the direct conversion of sulfides into NH‐sulfoximines by a one‐pot NH‐ and O‐transfer.^[39]^ The remarkable transformation was achieved efficiently on several alkyl, aryl, benzyl, cycloalkyl, heteroaryl sulfides **7**, leading to the corresponding sulfoximines **3** with excellent yields (Scheme 9). The method was further validated by using several sources of ammonia (ammonium acetate, NH_3_ in methanol, ammonium carbonate) including the cheap and readily available ^15^N‐ammonium acetate, which afforded ^15^N‐labeled NH‐sulfoximines of biologically relevant compounds such as biotin (**3** **c**), methionine (**3** **d**), and a dipeptide (**3** **e**) (Scheme 9). At a similar time, Reboul reported a detailed mechanistic investigation of the one‐pot NH‐ and O‐transfer to sulfides, in an almost identical reaction developed independently.^[40]^ A detailed HRMS and NMR investigation identified sulfanenitrile species **V** and **VI** (Scheme 10) as key intermediates in the conversion of sulfides into the corresponding NH‐sulfoximines.

According to previous observations,^[28]^ the mechanism proposed by Reboul (Scheme 10) involved the short‐lived iodonitrene **II** that reacts with the sulfide to generate the sulfilimine iodonium species **IV**. Further attack of the methoxy or acetate anion to **IV** leads to methoxy or acetoxy‐λ^6^‐sulfanenitriles **V** or **VI** respectively. Sulfanenitrile **V** may undergo nucleophilic attack, operated by methanol, producing dimethylether and the corresponding NH‐sulfoximine **3**. Similarly, sulfanenitrile **VI** may behave as an acetylating agent reacting either with sulfoximine or methanol, leading to N‐acyl‐sulfoximine **8** and NH‐sulfoximine **3** (Scheme 10). The proposed mechanism highlights the roles of both methanol and acetate as oxygen donors. The progress of the reaction was monitored by HRMS analysis, detecting both methoxy‐λ^6^‐sulfanenitrile **V** and acetoxy‐λ^6^‐sulfanenitrile **VI**. Moreover, combination of ^15^N and ^2^H labelling, and multinuclear (^15^N, ^13^C, ^1^H) NMR experiments supported the proposed mechanism and the role of sulfanenitrile intermediates **V** and **VI**.^[41]^

**Scheme 11:**
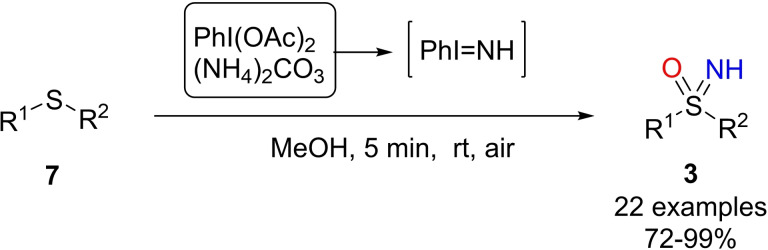
Synthesis of NH‐sulfoximines from sulfides, (diacetoxyiodo)benzene and ammonium carbonate.

Li and collaborators extended the one‐pot NH‐ and O‐ transfer methodology for accessing NH‐sulfoximines from sulfides **7** (Scheme 11).^[42]^ A detailed screening on nitrogen sources, oxidizing agents, and solvents was conducted, identifying ammonium carbonate (1.5 equiv.) and (diacetoxyiodo)benzene (2.3 equiv.) as a suitable combination, for the preparation of NH‐sulfoximines from sulfides, also using methanol as the reaction solvent (Scheme 11). Satisfactory results were also achieved by employing ammonium oxalate, ammonium fluoride, ammonium formate, and benzoate. Concerning the oxidant, (bis(trifluoroacetoxy)iodo)benzene, NCS, NBS, molecular iodine, iodosylbenzene, 2‐iodoxybenzoic acid, and 1,3‐dichloro‐5,5‐dimethylhydantoin were found to be ineffective.

**Scheme 12:**
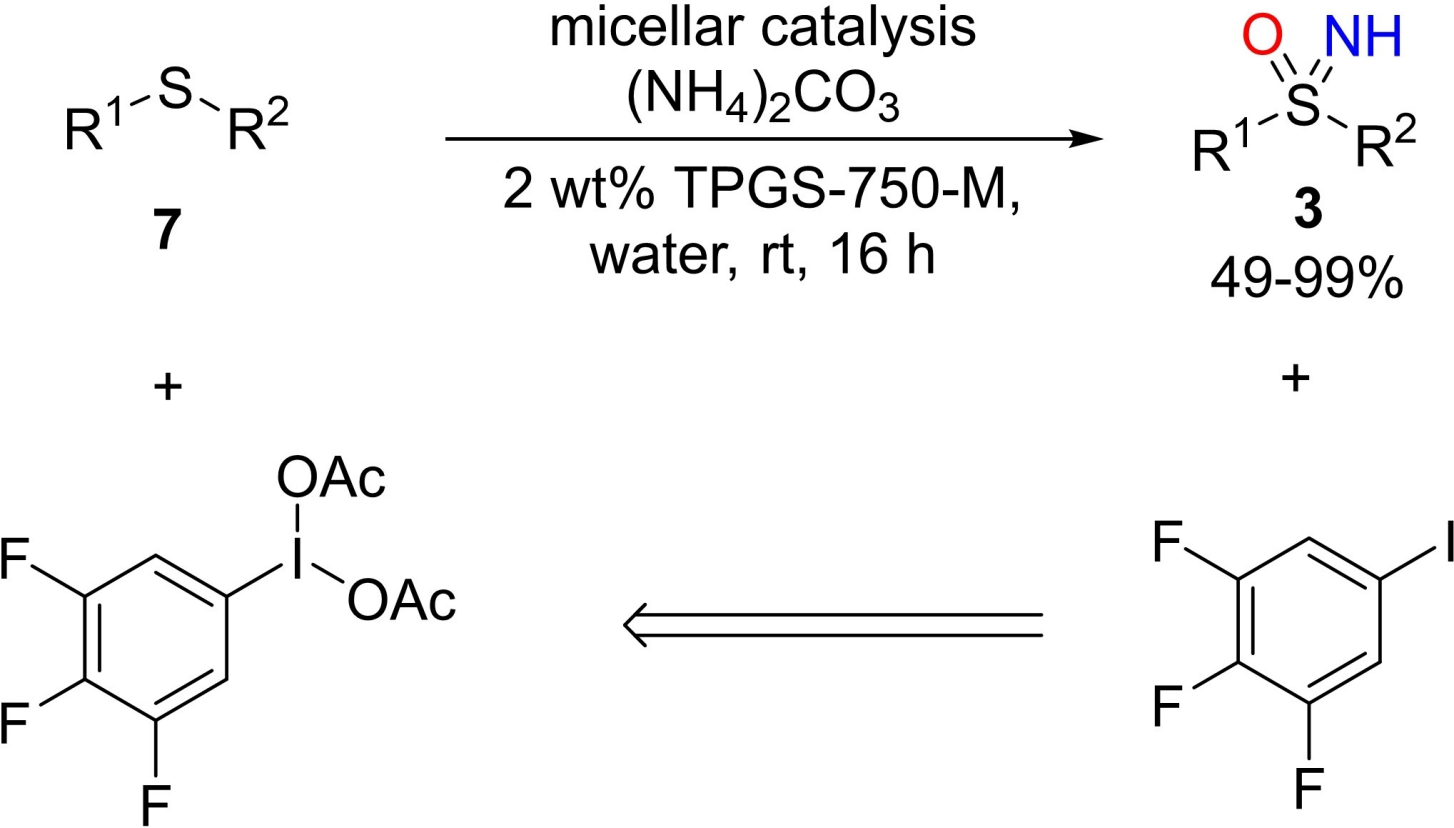
Micellar synthesis of NH‐sulfoximines by using a recyclable oxidizable reagent.

**Scheme 13:**
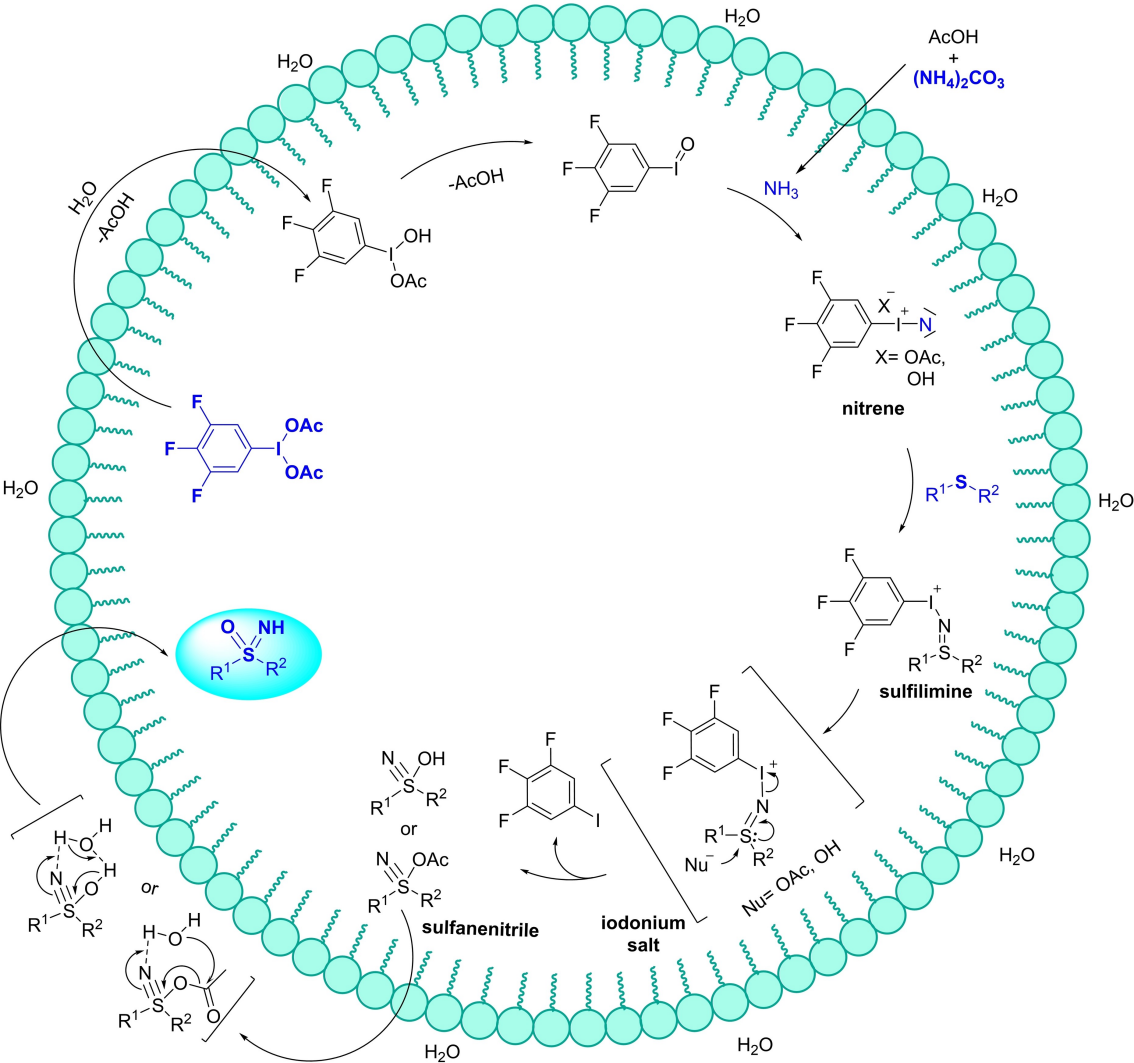
Proposed mechanism under aqueous micellar conditions for NH‐sulfoximines synthesis from sulfides.

Zheng and Xu reported a strategy for the synthesis of NH‐sulfoximines **3** starting from sulfides **7**, using the combination of hypervalent iodine (III) reagent and nitrogen source under aqueous conditions (Scheme 12).^[43]^ The one pot NH‐ and O‐transfer reaction was conducted in nanomicelles. The authors employed several surfactants (TPGS‐750‐M, PEG‐400, tween 80, Nok) observing better yields using 2 wt% TPGS‐750‐M. Ammonium carbonate was selected as nitrogen source due to the high aqueous solubility. With the aim to develop a more sustainable method, the recycling of the hypervalent iodine (III) reagent was pursued. In particular, the efficient permeation inside the micelles of lipophilic (diacetoxytrifluoro)iodobenzene provided high yields in NH‐sulfoximines. However, concentrated ammonia was required to consume the excess of oxidant promoting dissolution in the aqueous phase of the resulting sulfoximine. The extraction of the aqueous phase with organic solvents allowed recovery of the trifluoroiodobenzene, that could be re‐used upon oxidation with sodium perborate tetrahydrate and trifluoromethanesulfonic acid in acetic acid.^[44]^ This new protocol was found to be efficient with several aryl, heteroaryl, and alkyl sulfides, forming the corresponding NH‐sulfoximines with good to excellent yields. The scalability of the process and the application to biologically relevant compounds were demonstrated.^[45]^ The mechanism of the reaction was proposed to be closely related to that previously reported, forming an iodonitrene intermediate by reacting trifluoroiodosylbenzene with ammonia. Reaction of the iodonitrene intermediate with sulfide affords a sulfilimine which undergo nucleophilic attack of acetate anion or water to release a sulfanenitrile. Finally, the attack of water is expected to occur outside the micelle affording the desired sulfoximine (Scheme 13).

**Scheme 14:**
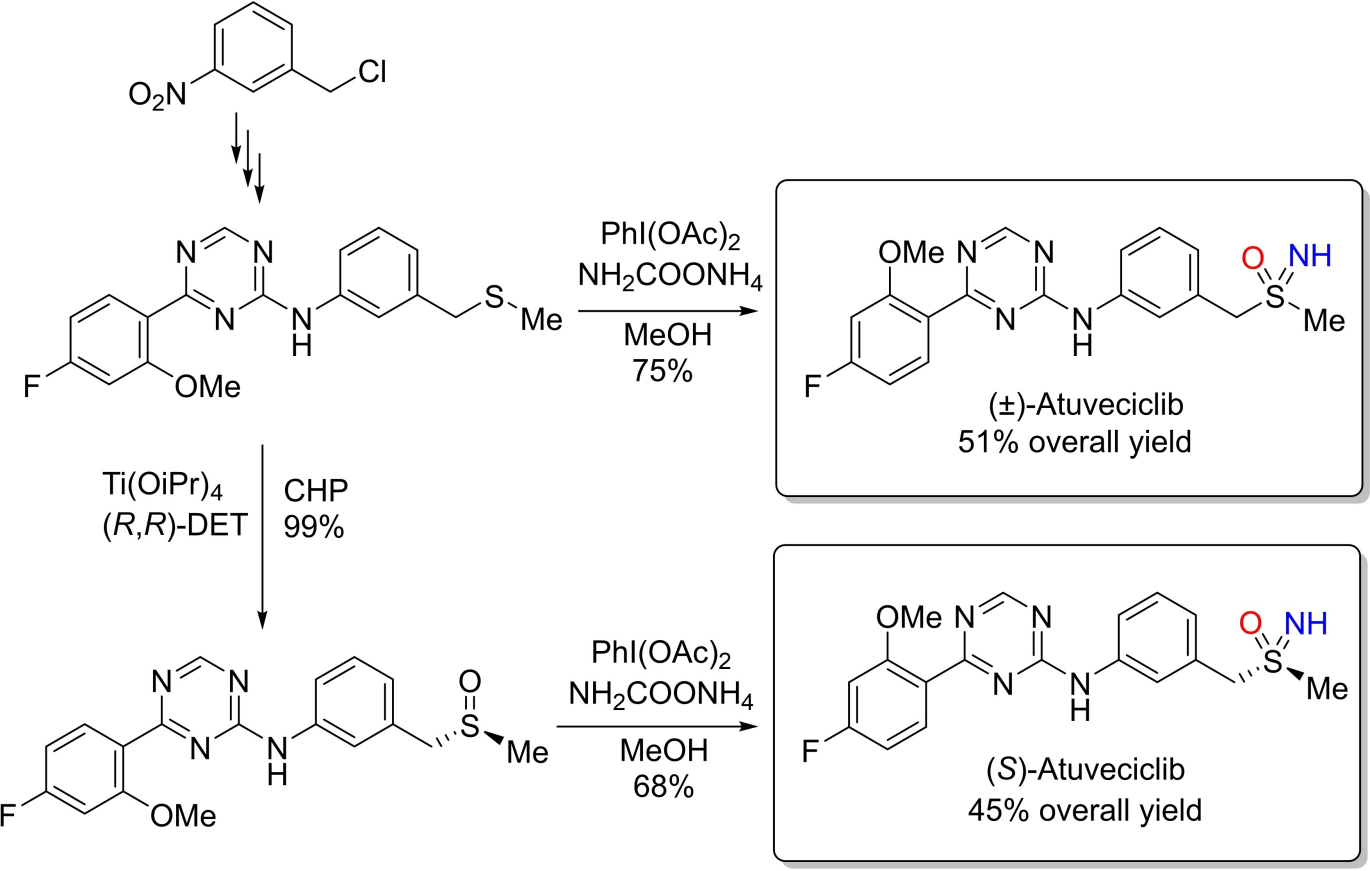
Late‐stage sulfoximination for the synthesis of Atuveciclib.

The one‐pot *NH*‐ and *O*‐transfer strategy has been rapidly adopted into the armoury of synthetic methods, and employed for the preparation of biologically relevant molecules. In 2019, Reboul reported a novel multistep strategy for the preparation of Atuveciclib, a PTEFb/CDK9 inhibitor, and a promising drug for cancer therapy[^46^, ^47^] Reboul described a synthetic approach involving a late‐stage sulfoximination of a sulfide by applying standard reaction conditions (2.1 equiv. of PhI(OAc)_2_, 1.5 equiv. of ammonium carbamate, in methanol at room temperature for 30 minutes). Interestingly, the final product was obtained in 51 % overall yield as racemic mixture (Scheme 14).^[46]^ Moreover, enantioenriched (*S*)‐Atuveciclib was obtained in a satisfactory 45 % yield using the N‐transfer conditions adopted with sulfoxides.^[28]^

**Scheme 15:**
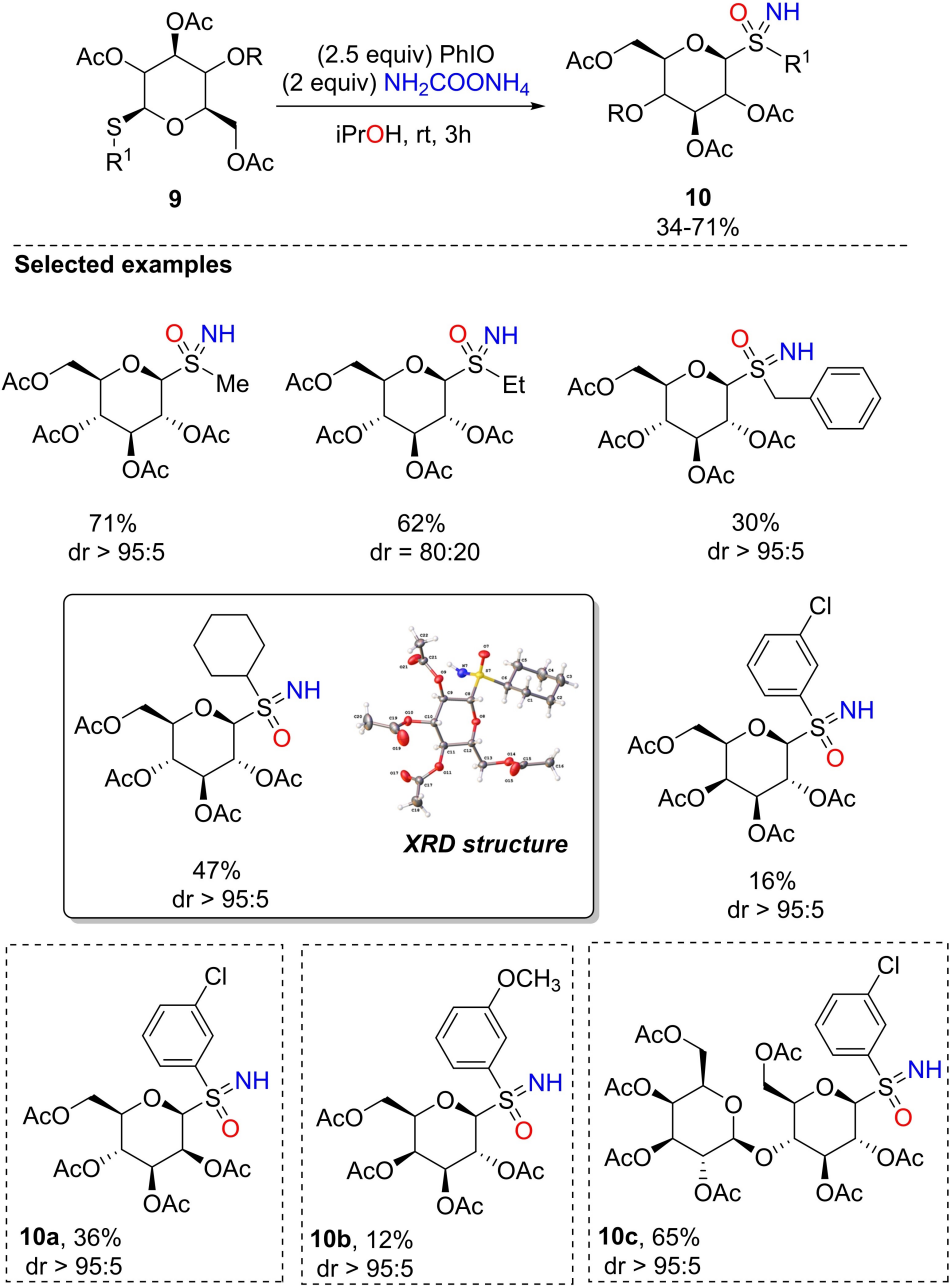
Synthesis of glycosyl *NH*‐sulfoximines.

Luisi, Bull, and Rollin developed a straightforward method to access unprecedented glycosyl sulfoximines **10**, via the one‐pot NH‐ and O‐ transfer to anomeric thioglycosides **9** (Scheme 15).^[48]^ Peracetylated *S*‐methyl‐β‐glucopyranoside, tested as a model substrate, was transformed into the corresponding NH‐sulfoximine by using 2.5 equiv. of iodosylbenzene, and 2 equiv. of ammonium carbamate, in *i*PrOH at room temperature for 3 h (Scheme 15). Methanol was an unsuitable reaction solvent due to a competitive formation of the corresponding *O*‐methyl glucopyranoside, likely resulting from displacement of sulfonimidoyl group. The scope of the reaction was explored, disclosing a good tolerance for aryl and cycloalkyl S‐substituents (Scheme 15). Remarkably, the reaction proceeds with good to excellent stereoselectivity (dr up to 95 : 5), while a slightly low stereoselectivity (dr=70 : 30) was observed when electron‐withdrawing substituents were installed on the aromatic ring S‐substituent. The stereochemistry at the sulfur atom was established by X‐ray analysis and computational models. The structural variability was additionally explored by modifying the sugar portion, as for peracetylated mannose (**10** **a**), galactose (**10** **b**) and lactose (**10** **c**) (Scheme 15).

**Scheme 16:**
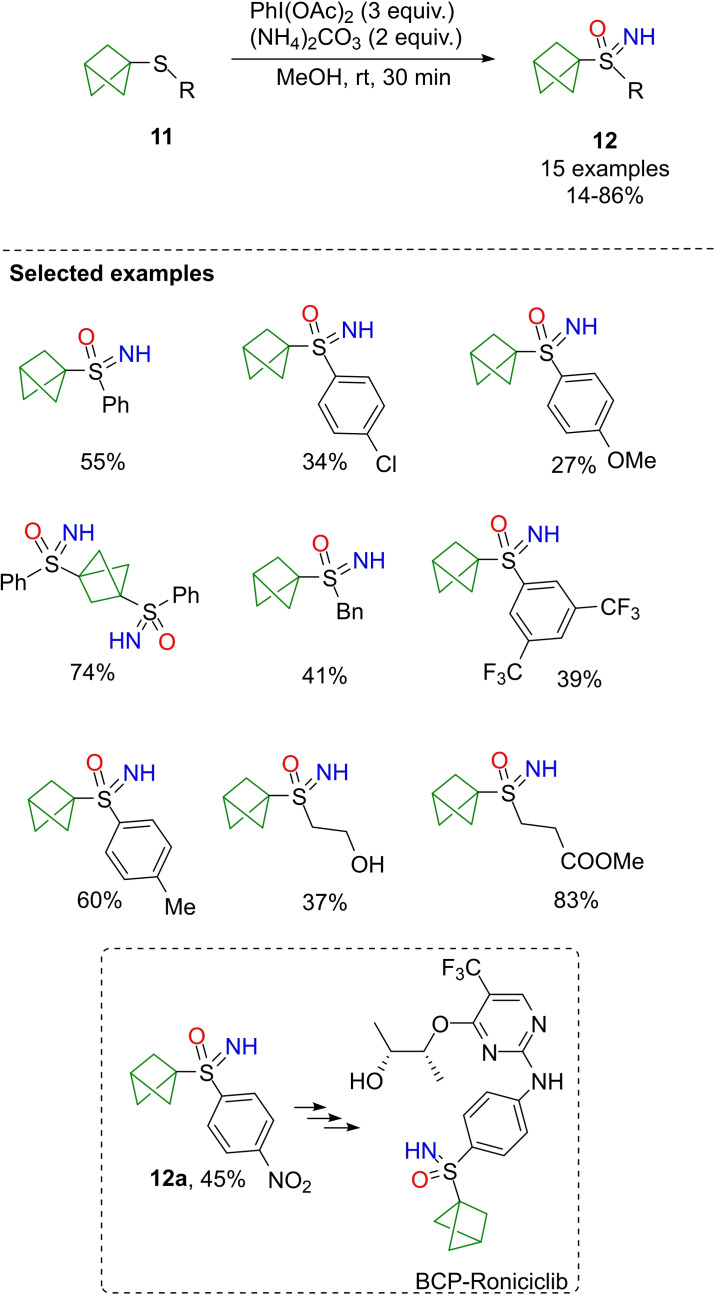
Synthesis of bicyclo[1.1.1]pentyl sulfoximines.

An interesting application of the one pot NH‐ and O‐transfer methodology has been reported by Bräse, who developed the synthesis of bicyclo[1.1.1]pentyl (BCP) sulfoximines **12** starting from the corresponding BCP sulfides **11** (Scheme 16).^[49]^ These new structural motifs are of interest in drug discovery as 3D mimics of aromatic rings.^[50]^ The optimal reaction conditions required a large excess of the oxidant (3 equiv. of PhI(OAc)_2_) and 2 equiv. of ammonium carbonate. The reaction was tolerant to several functional groups furnishing good yields of the corresponding BCP sulfoximines. However, the reaction was sensitive to steric hindrance at the sulfur atom. The protocol was applied to the preparation of *p*‐nitrophenyl substituted BCP sulfoximine **12** **a**, a precursor for the synthesis of a BCP‐analogue of Roniciclib (Scheme 16).^[51]^

**Scheme 17:**
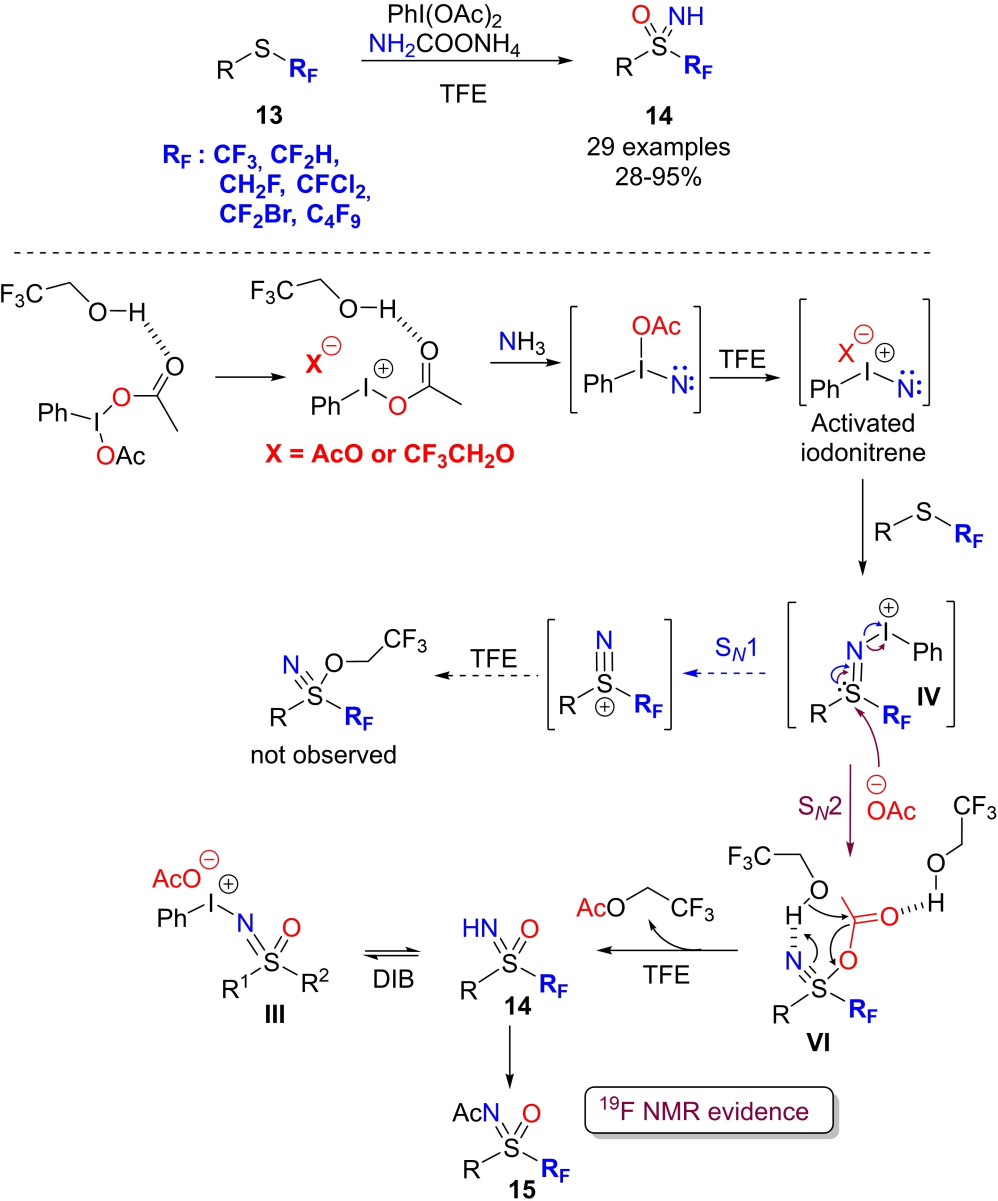
One‐pot preparation of S‐fluoroalkylated NH‐sulfoximines from sulfides.

Fluorinated sulfoximines show interesting applications in synthetic chemistry, as nucleophiles,^[52]^ radical transfer agents,^[53]^ directing groups,^[54]^ and building blocks for liquid crystals preparation.^[55]^ However, efficient methods for accessing fluorinated sulfoximines have been introduced only recently. Reboul and Magnier developed a general approach for the synthesis of S‐fluoroalkylated NH‐sulfoximines **14** from fluoroalkylsulfides **13**.^[56]^ This metal‐free strategy adopts the one pot NH‐ and O‐transfer to sulfides by using ammonium carbamate (1.5 equiv.) as nitrogen source, DIB (2.1 equiv.) as the oxidizing agent, with trifluoroethanol (TFE) as polar and hydrogen‐bond donor solvent (Scheme 16). The optimal reaction conditions achieved high conversion of the relatively poorly nucleophilic sulfides but formed a mixture of NH‐sulfoximines **3** and N‐acetyl (N−Ac) sulfoximines **15**. A final deprotection step by treatment with HCl provided the desired fluoroalkylated NH‐sulfoximines. Satisfactory results were obtained with several fluorinated alkyl and aryl sulfides, and the process was scalable up to 12 mmol. The protocol was effective with sulfides bearing a perfluorobutyl, CF_2_Br, CFCl_2_, CF_2_H, and CH_2_F groups. The reaction was subjected to a deep mechanistic investigation by ^19^F NMR and HRMS analysis. The reaction with (4‐methoxyphenyl)difluoromethyl thioether as model substrate, was monitored by ^19^F NMR, and the signals of NH‐sulfoximine **3** and and NAc‐sulfoximine **15** were observed, as well as those of sulfanenitrile **VI** and iodonium salt **III** (Scheme 17). An activated nitrene intermediate was proposed that reacted with the sulfide leading to sulfilimine **IV**. Nucleophilic attack of acetate anion to **IV** afforded the short‐lived sulfanenitrile **VI**. The trifluoroethanol solvent was proposed to play an active role in forming sulfoximine **3**, either in reaction with with DIB and/or with the sulfanenitrile giving compounds **III** and **6** respectively (Scheme 17).

**Scheme 18:**
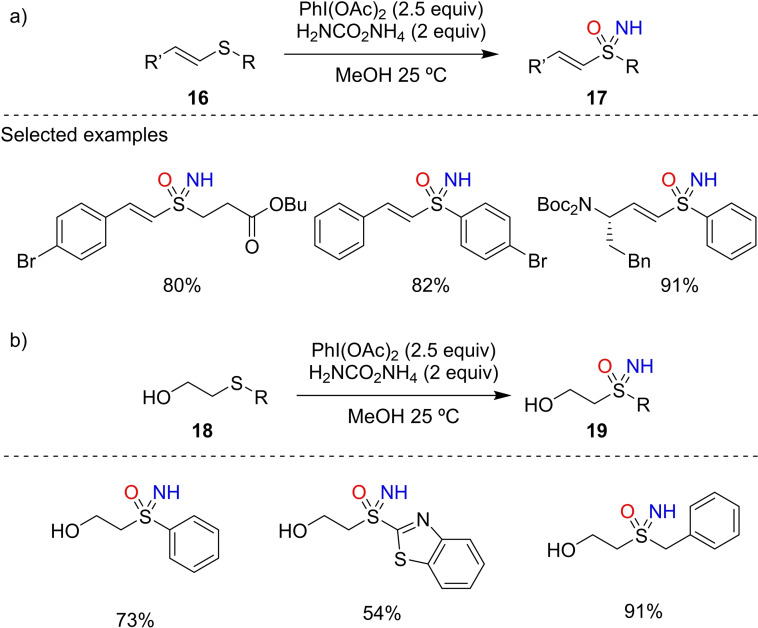
a) Synthesis of substituted vinyl sulfoximines from vinyl sulfides. b) Synthesis of β‐hydroxysulfoximined as substrates for elimination.

Very recently, Craven et al. reported several strategies for the preparation the vinyl sulfoximines.^[57]^ Vinyl sulfoximines offer interesting potential as chiral electrophilic warheads in covalent inhibitors, that can also incorporate additional functionality through the nitrogen group to provide fully functionalized probes. Substituted vinyl sulfoximines **17** were generated directly from vinyl sulfides **16**, by NH and O transfer, again indicating the very high chemoselectivity of this reaction (Scheme 18, a). To form terminal vinyl sulfoximines **19**, given the relative instability of the vinyl sulfide, the sulfoximine group was formed on β‐hydroxysulfides **18** (Scheme 18, b). Treatment of the β‐hydroxysulfoximine products with MsCl effected elimination to the terminal vinyl sulfoximines.

**Scheme 19:**
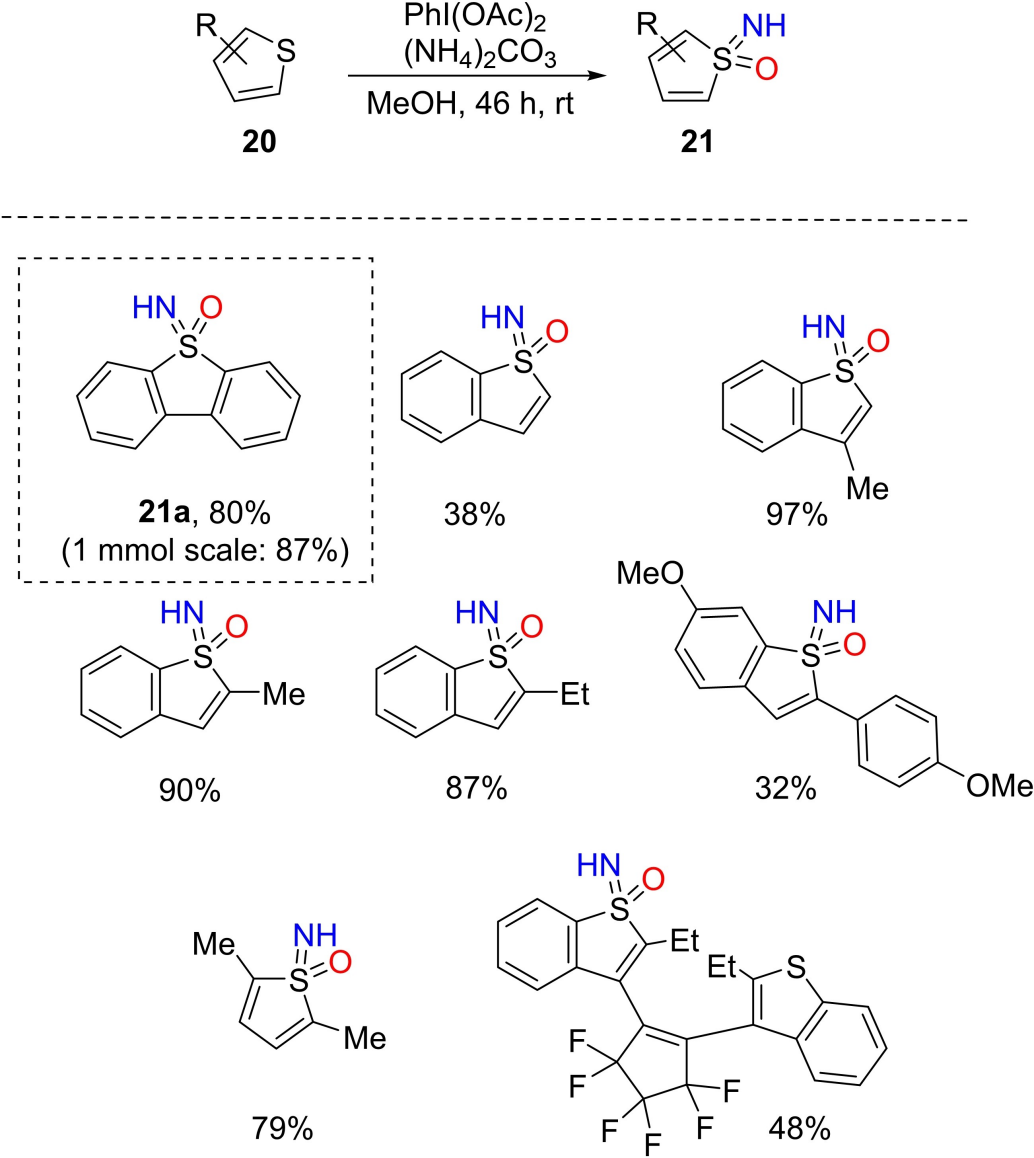
One‐pot NH‐ and O‐transfer to thiophene derivatives.

Due to the relevance of thiophene sulfones in the field of photovoltaics,^[58]^ or as fluorophores,^[59]^ and photoswitches,^[60]^ Bolm and co‐workers investigated the synthesis of thiophene NH‐sulfoximines.^[61]^ In order to achieve the contextual imination/oxidation at the sulfur atom of thiophene, the authors applied the one‐pot NH‐ and O‐transfer methodology for the preparation of the corresponding NH‐sulfoximine. By using a large excess of DIB (5 equiv.) and ammonium carbonate (3 equiv.), dibenzothiophene furnished the corresponding NH‐sulfoximine **21** **a** in 80 % of yield (Scheme 19). The reaction was further applied to thiophenes **20** substituted at C2 or C3, obtaining the corresponding NH‐sulfoximines **21** in high yields.

**Scheme 20:**
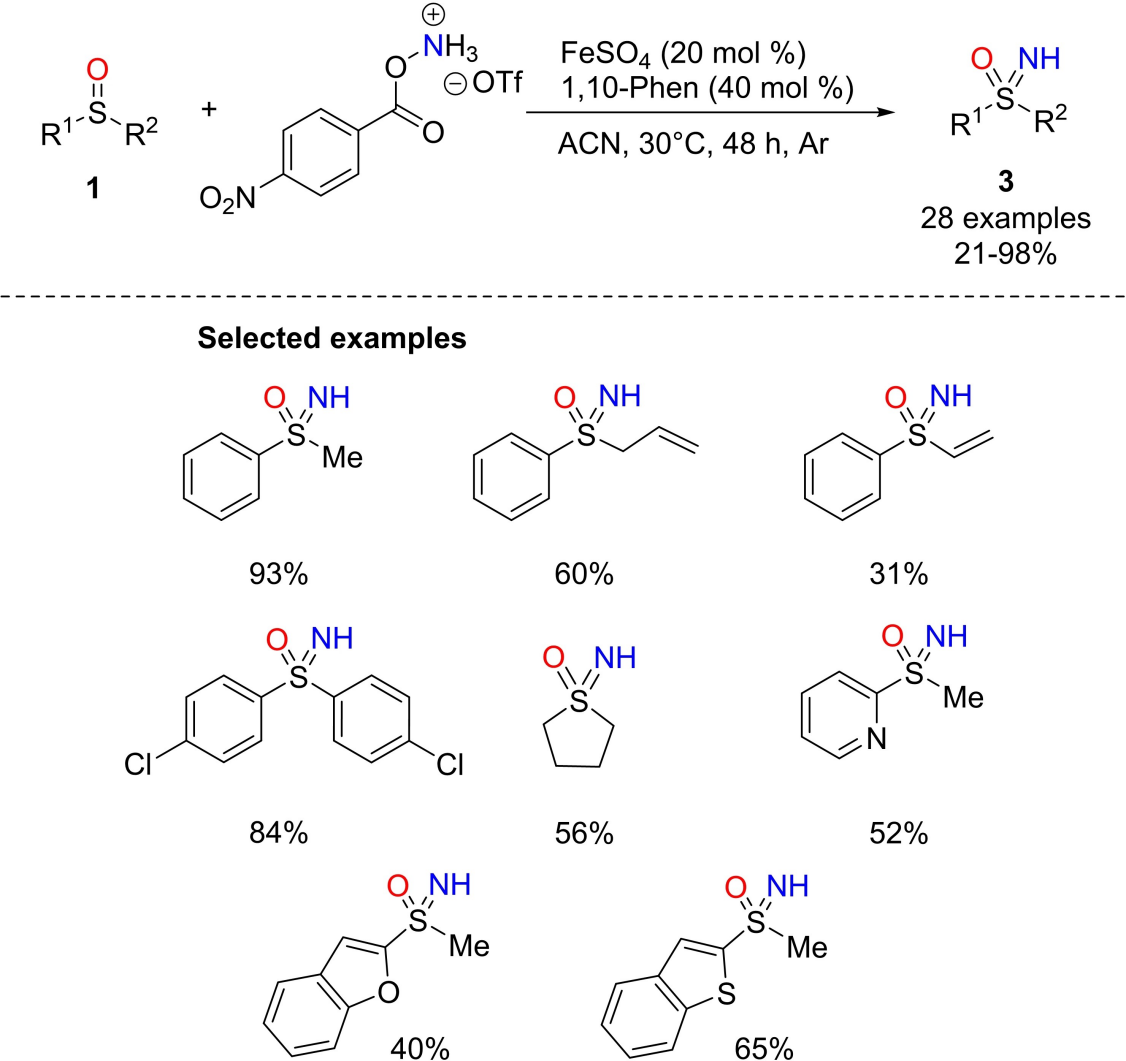
Synthesis NH‐sulfoximines from sulfoxides by iron (II) catalysis.

In 2018, Bolm and coworkers reported a Fe(II)‐catalyzed method for the direct preparation of NH‐sulfoximines from sulfoxides.^[62]^ This strategy involved the use of FeSO_4_/phenanthroline (with a loading from 20 %mol to 40 %mol), and an arylhydroxylamine derivative as the NH‐donor in acetonitrile at 30 °C under argon atmosphere (Scheme 20). In this procedure, the use of a bench‐stable aminating agent avoids the use of oxidants. The imination protocol furnished good to high yields (70‐98 %) with several S‐aryl, S‐alkyl substituted sulfoxides **1** (Scheme 20). Moreover, the protocol enables the preparation of NH‐sulfoximines **3** bearing various heterocycles (2‐pyridinyl, benzofuranyl, benzothienyl, and indolyl) as the S‐substituents.[^63^, ^64^] The authors proposed an iron nitrene complex as key reaction intermediate, to transfer the nitrogen to the sulfur atom of the sulfoxide.

**Scheme 21:**
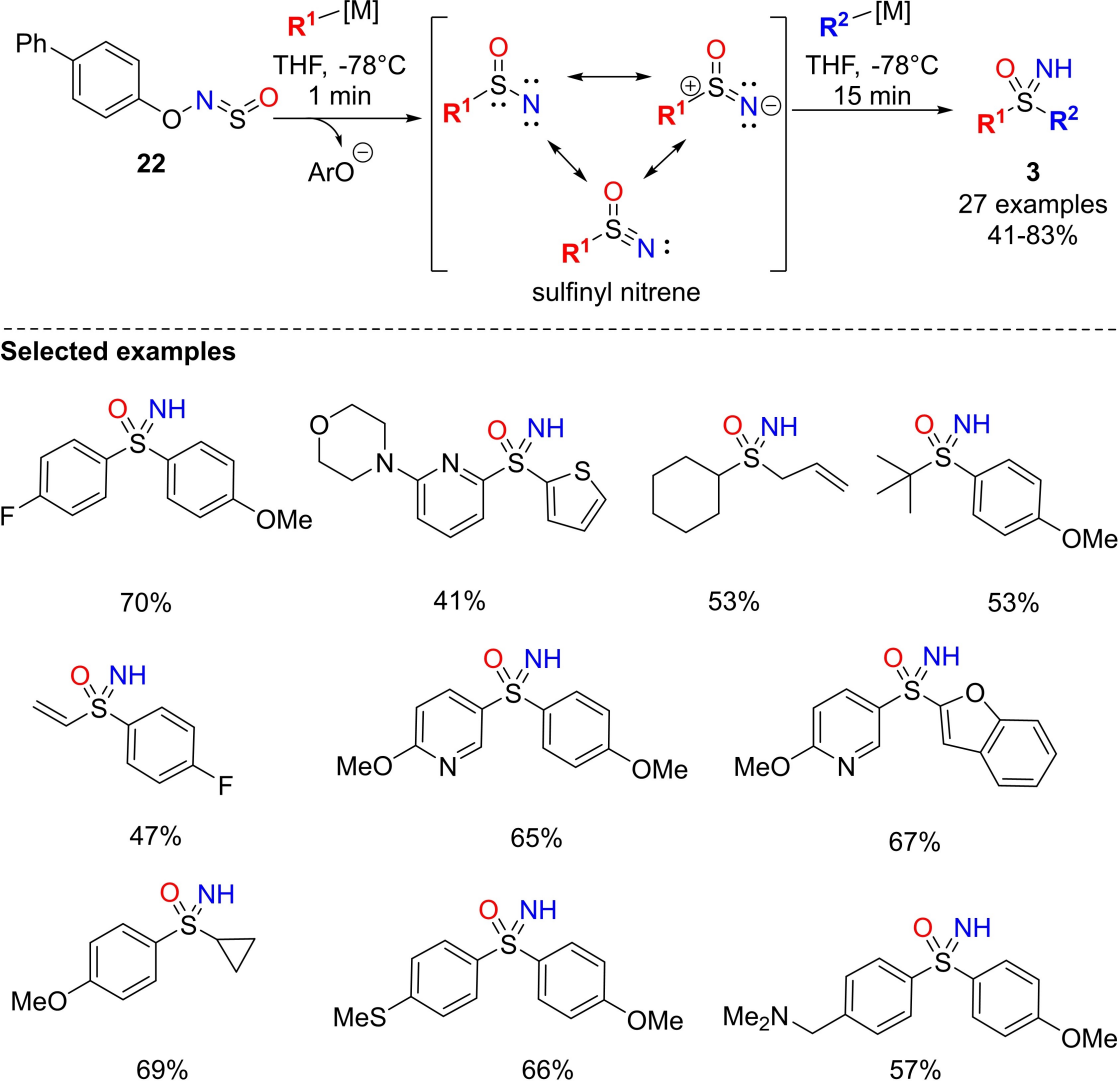
Synthesis of NH‐sulfoximines via sulfinyl nitrene generation.

Very recently, Willis and co‐workers reported the preparation of NH‐sulfoximines exploiting the generation and trapping of an unprecedented electrophilic sulfinyl nitrenes.^[65]^ The protocol involves the utilization of sulfinylhydroxylamine reagent **22** that provided the reactive sulfinyl nitrene upon treatment with organolithium or Grignard reagents through an N−O bond fragmentation process. The subsequent addition of a different carbon nucleophile enables the preparation of the corresponding sulfoximines **3** in moderate to good yields (Scheme 21). The scope of the reaction has been widely explored preparing sulfoximines bearing functionalized aryl, heteroaryl, alkyl, vinyl and allyl substituents. Interestingly, the one‐pot reaction proceeds rapidly, affording the desired products within 16 min in THF at −78 °C. Moreover, the addition of an electrophile after the reaction with the second carbon nucleophile resulted into the direct preparation of N‐functionalized sulfoximines in good yields.

**Scheme 22:**
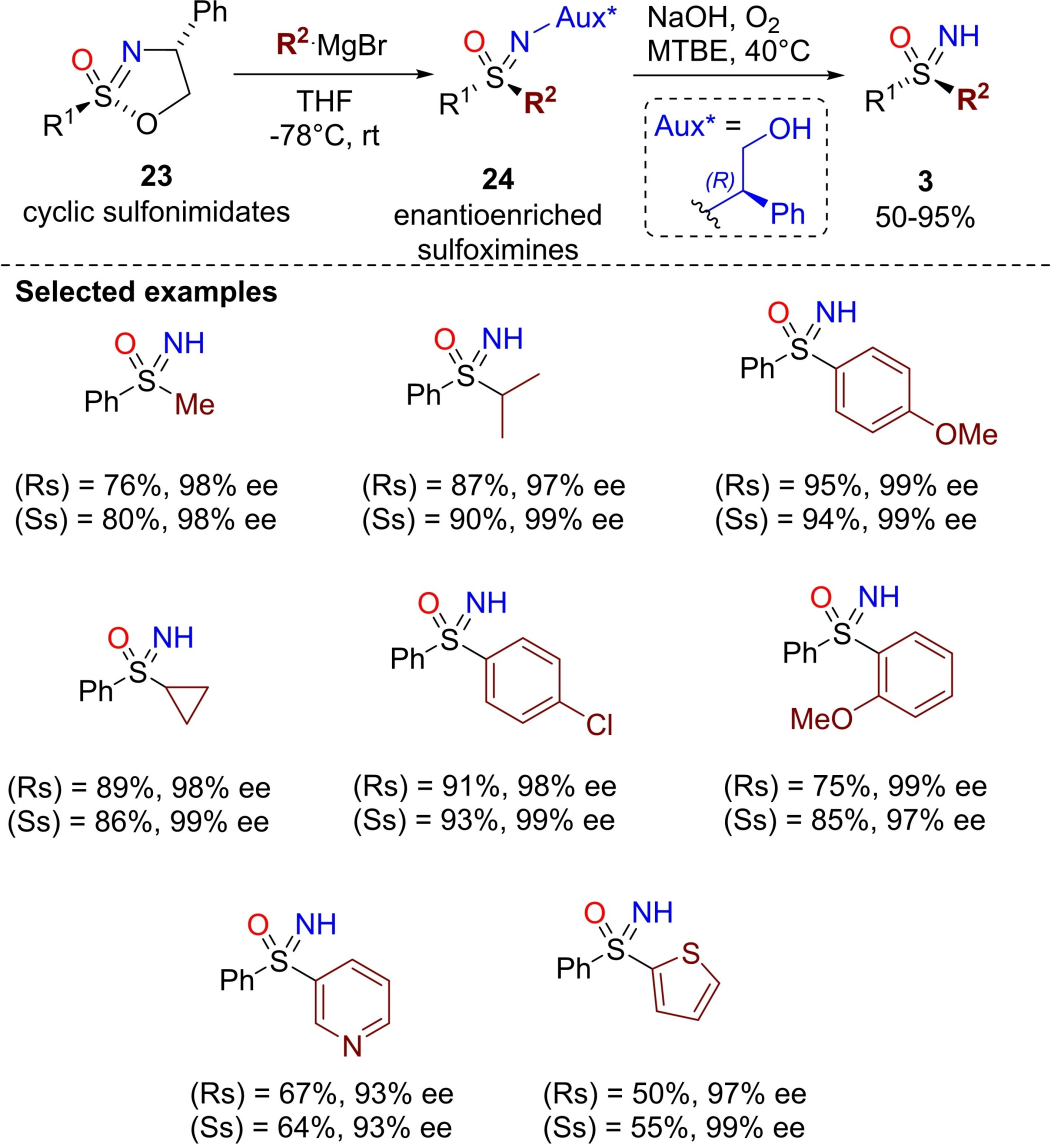
Synthesis of optically active NH‐sulfoximines from cyclic sulfonimidates.

An efficient method for the synthesis of enantioenriched NH‐sulfoximines, from optically active cyclic sulfonimidates, has been recently described by Stockman and Moore.[^66^, ^67^] The required chiral enantioenriched sulfonimidates were obtained from the corresponding sulfinamides, in turn prepared from sulfinyl chlorides and (*R*)‐phenyl glycinol. This was followed by intramolecular cyclization upon treatment with N‐chlorosuccinimide (NCS) or *tert*‐butyl hypochlorite (*t*BuOCl) and typically separation of the S‐diastereoisomers. The authors optimized the ring opening of sulfonimidates **23** with Grignard reagents en route to sulfoximines **24** (Scheme 22). S‐Methyl sulfonimidates furnished a mixture of diastereoisomers of the corresponding sulfoximines due to a competitive elimination causing ring opening and loss of S‐stereochemistry with subsequent attack to the methylene derivative, which resulted in racemization at the sulfur center. On the other hand, S‐aryl sulfonimidates reacted with high stereospecificity, affording sulfoximines as single diastereoisomers with inversion of configuration at the sulfur center. Alkyl, aryl, and heteroaryl (thienyl, pyridyl) Grignard reagents were suitable for sulfonimidate ring‐opening reactions. Removal of the chiral auxiliary upon treatment with oxygen and NaOH in methyl *tert*‐butyl ether (MTBE) afforded highly enantioenriched NH‐sulfoximines **3** in good yields (Scheme 22). ^[68]^

**Scheme 23:**
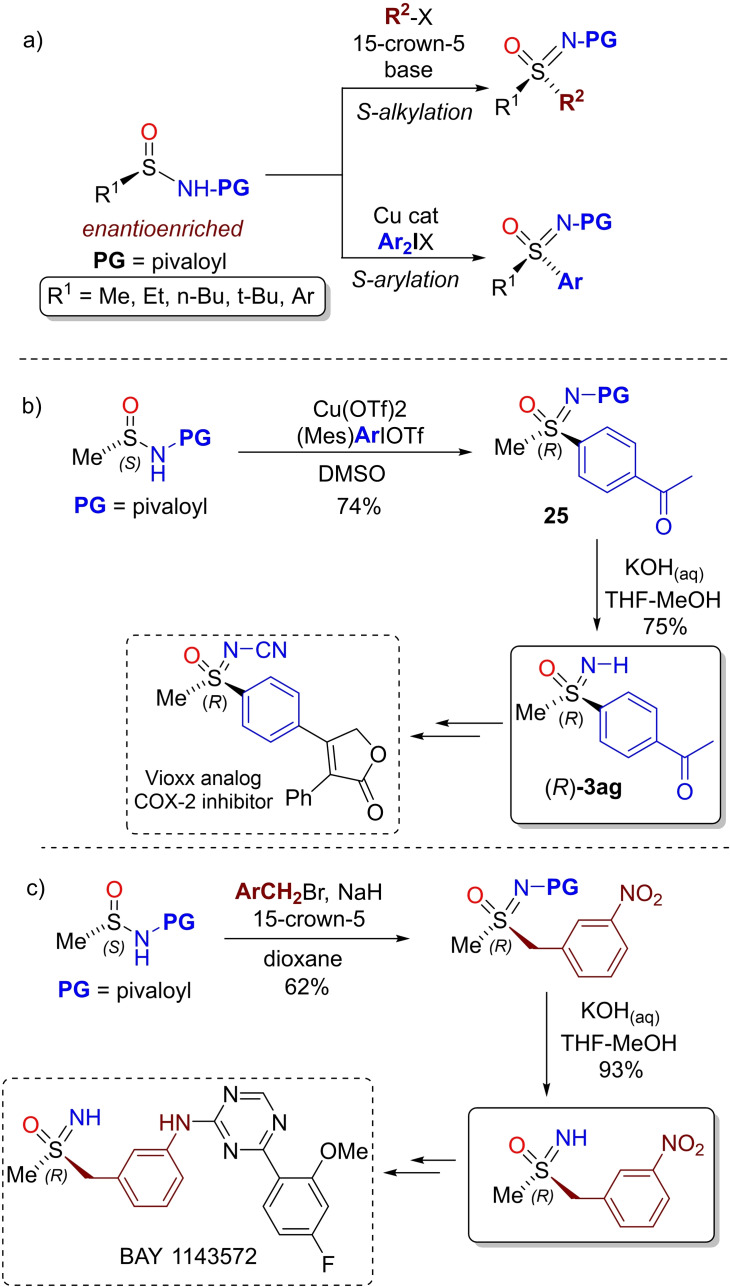
Synthesis of chiral optically active NH‐sulfoximines from sulfinamides via S‐alkylation and S‐arylation.

Maruoka and Kano reported a powerful alternative approach, based on the S‐arylation and S‐alkylation of sulfinamides, for the asymmetric synthesis of chiral *N*‐pivaloyl sulfoximines (Scheme 23, a).^[69]^ The sulfur‐chemoselective alkylation was achieved under basic conditions in dioxane using alkyl iodides and bromides, chiral enantioenriched sulfinamides, and in the presence of 15‐crown‐5 ligand. The process allowed the preparation of N‐acylated sulfoximines in good yields and high enantioselectivity.^[70]^ A different approach was needed for the sulfur‐chemoselective arylation of chiral enantioenriched sulfinamides. In this case the S‐aryl substituent was introduced by using a suitable diaryliodonium salt in the presence of a copper catalyst. Once again chiral enantioenriched N‐acylated sulfoximines were obtained in good yields and optical purity. Interestingly, the availability of two protocols for S‐alkylation and arylation allowed access to both enantiomers of a given chiral sulfoximine by the judicious ordering of steps. Moreover, the authors developed effective protocols for the N‐deprotection for preparing highly enantioenriched NH‐sulfoximines **3** (Scheme 23, b). The potential of this synthetic strategy (S‐alkylation/arylation and deprotection) was demonstrated by the synthesis of an optically active analogue of the COX‐2 inhibitor Vioxx from sulfoximine **25**, and a precursor of the lead compound BAY 1143572 (Scheme 22, b,c).^[71]^

**Scheme 24:**
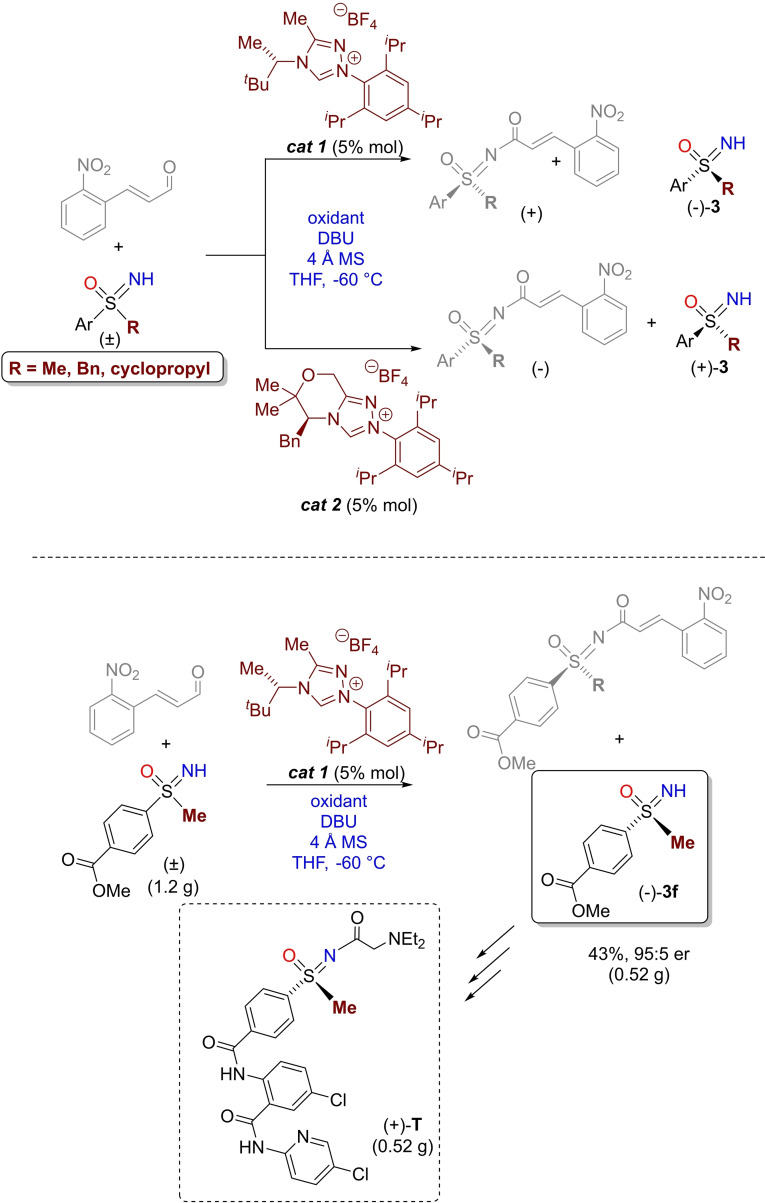
Synthesis of chiral optically active NH‐sulfoximines by NHC‐catalyzed kinetic resolution.

A highly selective kinetic resolution of racemic sulfoximines was recently developed by Bolm.^[72]^ The protocol employed racemic NH‐sulfoximines, an enal and a suitable chiral N‐heterocyclic carbene (NHC) catalyst. Two NHC catalysts, able to provide both enantiomers of chiral NH‐sulfoximines **3**, were identified for highly selective resolutions. The stereoselective amidation did not require additional acyl transfer agents, and the process could be run on gram scale. The usefulness of the methodology was demonstrated with the preparation of a human Factor Xa inhibitor (+)‐**T** (Scheme 24).

**Scheme 25:**
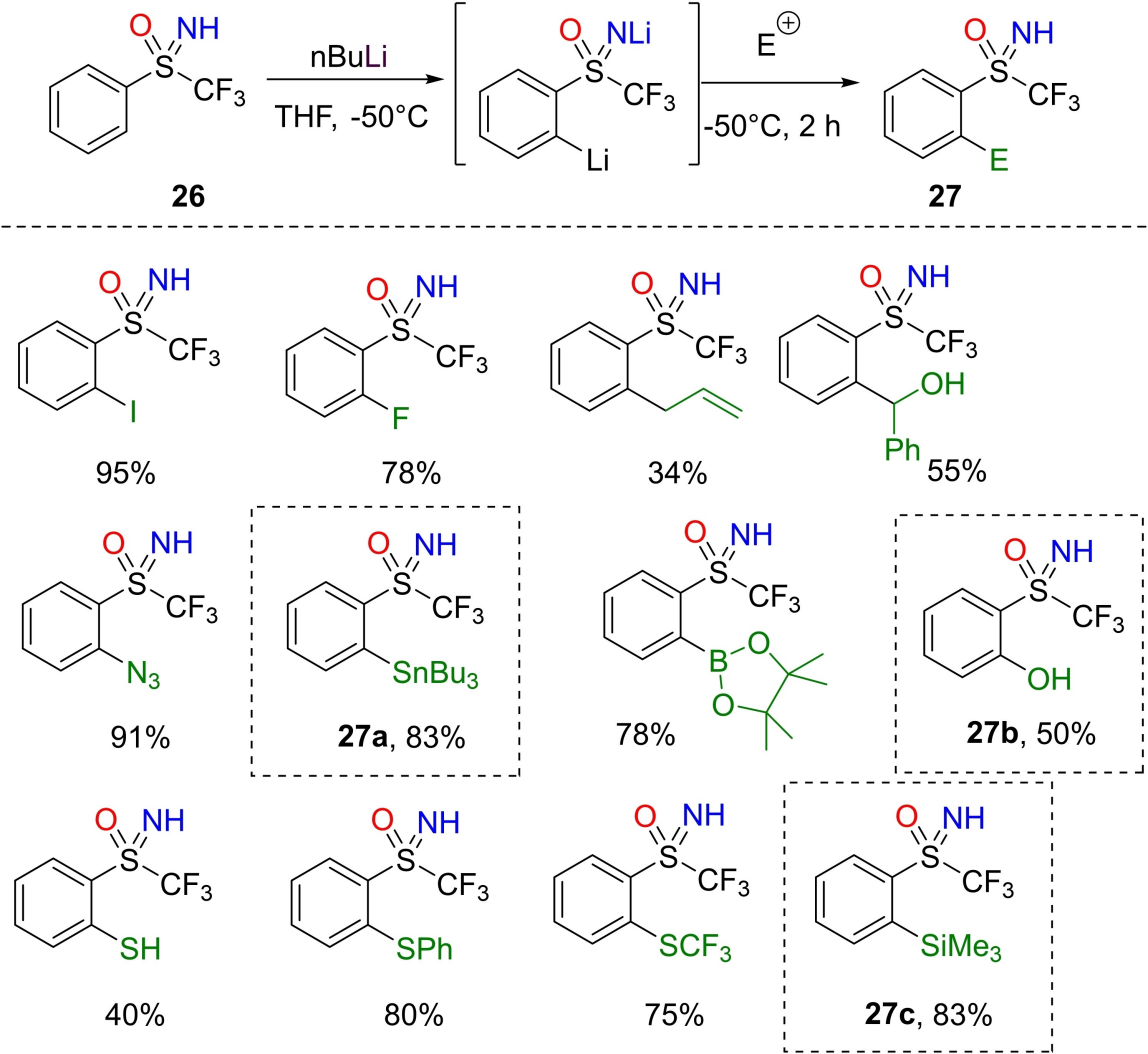
*ortho*‐Lithiation of S‐trifluoromethyl NH‐sulfoximines.

In 2016, Magnier and Vo‐Thanh reported that a perfluoroalkylated sulfoximidoyl moiety could behave as an *ortho*‐directing group in the lithiation‐trapping sequence of (hetero)arenes.^[73]^ Similarly to sulfones and sulfonamides, fluorinated sulfoximines exhibited directing metalation capability participating in the coordination of the lithium ion at the *ortho*‐position of aryllithium complexes. Under optimized conditions, *ortho*‐lithiation of NH‐sulfoximine **26** occurred by using 2 equivalents of *n*BuLi, in THF at −50 °C. Reasonably, the first equivalent of base removed the nitrogen proton, likely affecting the kinetics of the *ortho*‐lithiation step by the second equivalent of base.^[74]^ Therefore, upon reaction with electrophiles, *ortho*‐functionalized sulfoximines **27** were obtained in modest to excellent yields (Scheme 25). Several electrophiles including halogens (bromide, fluorine and iodine), azido, and pinacol borane moieties have been introduced with satisfactory results. The lithiation‐trapping sequence with B(OMe)_3_ and subsequent reaction with H_2_O_2_, led to the formation of interesting phenolic compound **27** **b**. Moreover, the stannylation with Bu_3_SnCl and silylation with TMSCl afforded the corresponding sulfoximines **27** **a** and **27** **c** in very good yields (Scheme 25).

**Scheme 26:**
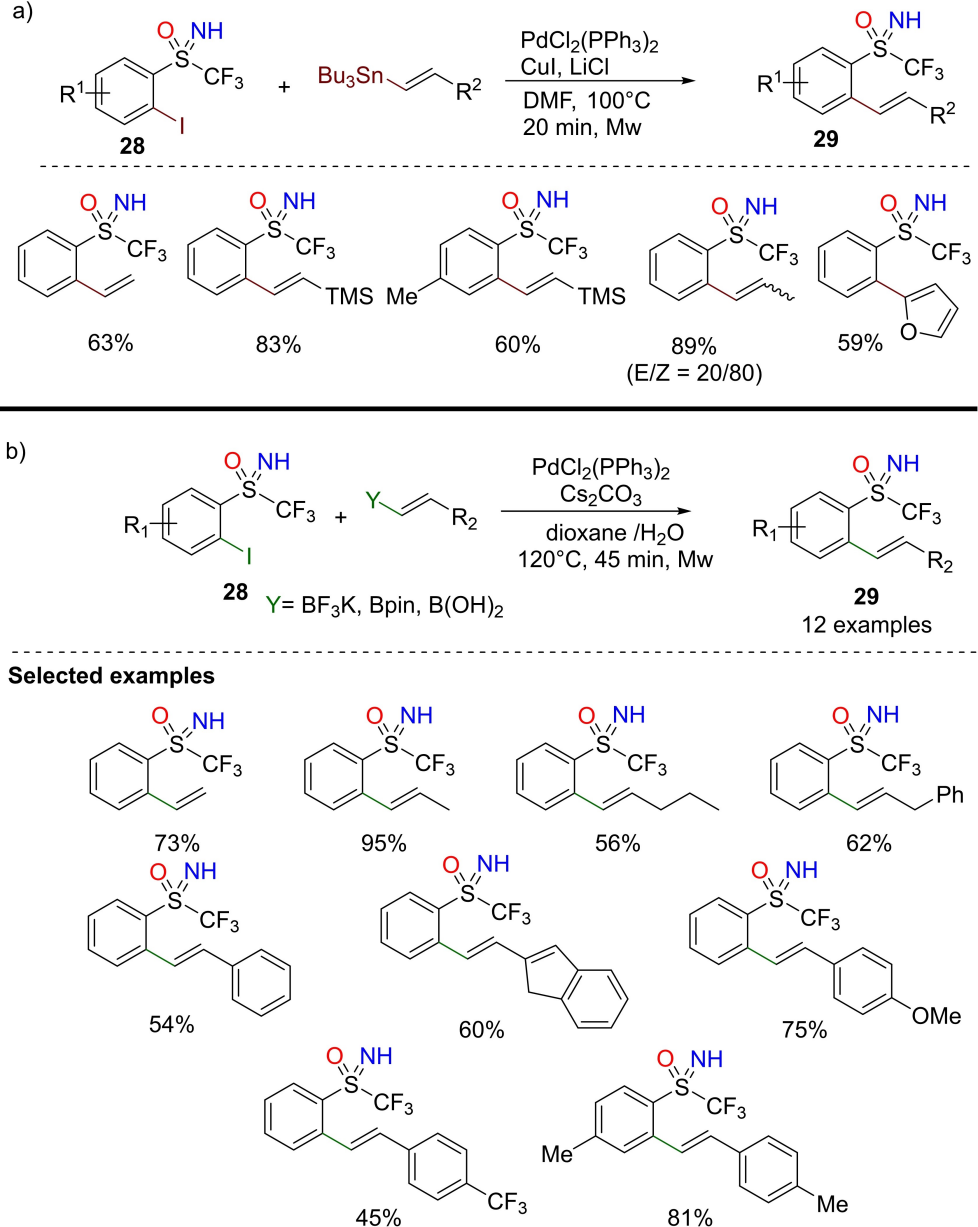
Synthesis of 2‐phenylvinyl‐S‐trifluoromethyl‐NH‐sulfoximines by cross‐coupling reactions.

Magnier and Anselmi described a modified Stille reaction under microwave conditions for the preparation of *ortho*‐vinylaryl‐trifluoromethylated NH‐sulfoximines.^[75]^ Several *ortho*‐vinylaryl sulfoximines **29** were obtained via the Pd‐catalyzed reaction of *ortho*‐iodoaryl sulfoximines **28** with vinylstannanes (Scheme 26, a). Similarly, the Pd‐catalyzed Sukuzi‐Miyaura cross coupling of vinylboron compounds with trifluoromethyl *ortho*‐iodoaryl NH‐sulfoximines was optimized under microwave conditions. Several functionalized trifluoromethyl‐ aryl‐substituted NH‐sulfoximines **29** were prepared in good yields (Scheme 26, b). The method represents a robust and effective alternative to access trifluorosubstituted NH‐sulfoximines. The robustness of the protocol was further demonstrated by gram‐scale preparations of trifluoromethylated NH‐sulfoximines without any substantial loss of yield.

**Scheme 27:**
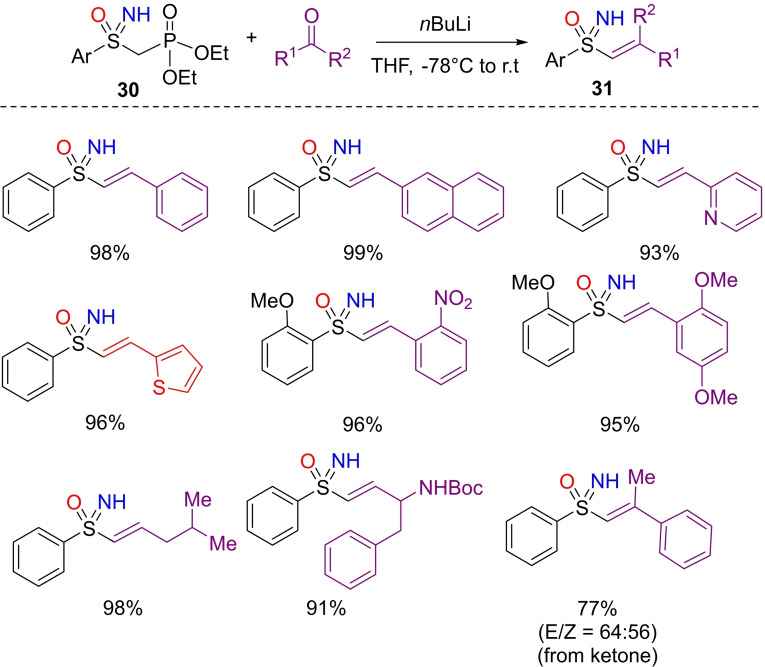
Synthesis of vinylic NH‐sulfoximines under HWE conditions.

Vinylation reactions represent an important tactic in organic synthesis. Vinyl sulfoximines have been widely exploited as chiral auxiliaries,^[76]^ ligands,^[77]^ Michael acceptors,^[78]^ as dienophiles in pericyclic reactions,^[79]^ and as precursors for the synthesis of allylic sulfoximines.^[80]^ The main approaches for the preparation of vinyl sulfoximines involve the hydroxyalkylation‐elimination of metalated alkyl sulfoximines^[81]^ and the carbometalation of alkynyl sulfoximines.^[82]^ In 2016, a new route to access vinyl NH‐sulfoximines was developed by Arvidsson and Naicker, who explored the reaction of diethyl(arylsulfonimidoylmethyl)phosphonates **30** with aldehydes, under Horner‐Wadsworth‐Emmons (HWE) conditions (Scheme 27).[^56^, ^83^, ^84^] Performing the reaction at −78 °C with *n‐*BuLi, the desired vinyl‐NH‐sulfoximines **31** can be obtained with complete *E*‐selectivity. Several functionalized aromatic, aliphatic aldehydes subjected to the HWE protocol, afforded the desired products in excellent yields. Interestingly, this approach is directly applicable to NH‐sulfoximines, avoiding additional protection/deprotection steps. As reported by Bharatam et al., the nature of the S=N double bond consists into a single covalent bond and a strong ionic interaction without any substantial *π*‐overlap.^[85]^ Consequently, *n*BuLi is expected to abstract the proton of the more acidic activated methylene group without reacting with the NH group.

## 3 Flow Technology Applications in the Synthesis of NH‐Sulfoximines

**Scheme 28:**
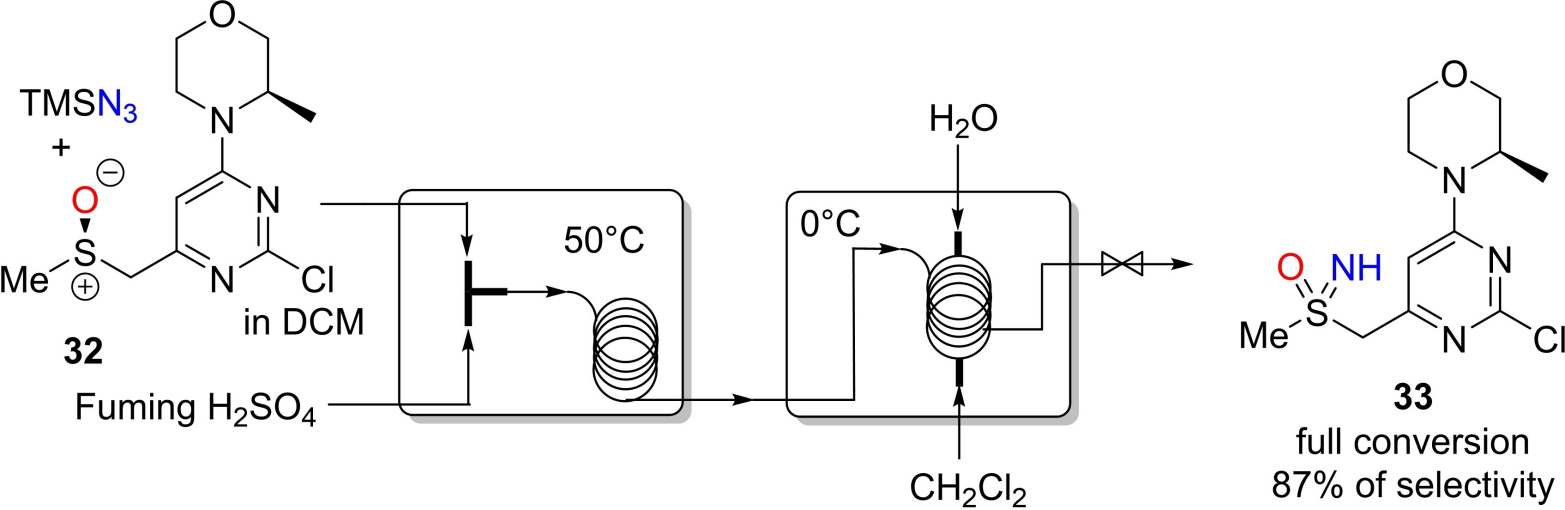
Continuous flow set‐up for sulfoxide imination with azidotrimethylsilane and fuming sulfuric acid.

The use of flow technology in the development of safer, cleaner, and more sustainable synthetic methodologies encompass procedures for the preparation of NH‐sulfoximines. In 2015, Kappe and coworkers reported the development of a continuous flow protocol for the direct synthesis of NH‐sulfoximine **23**, an intermediate in the early process routes for the synthesis of ATR kinase inhibitor AZD6738.[^34^, ^35^, ^36^] The low yields, poor selectivity, the formation of different side‐products and the safety concerns encountered using the conventional batch approach, led the authors to explore a continuous flow protocol. A mixture of sulfoxide and azidotrimethylsilane (TMSN_3_) and fuming sulfuric acid were introduced through two different feeds into a coil reactor at 50 °C (Scheme 28). For the in‐line quenching and extraction, water and dichloromethane were used. In striking contrast to batch protocol, the flow reaction using fuming sulfuric acid afforded the corresponding sulfoximine with 90 % selectivity after only 10 to 15 min of reaction time at 50 °C. However, racemization of the resulting NH‐sulfoximine **33** occurred under the strongly acidic conditions. As described by Olah, the protonation of hydrazoic acid in superacids afforded the H_2_N_3_^+^ species that acts as a strong electrophilic agent in the reaction with a sulfoxide.^[86]^

**Scheme 29:**
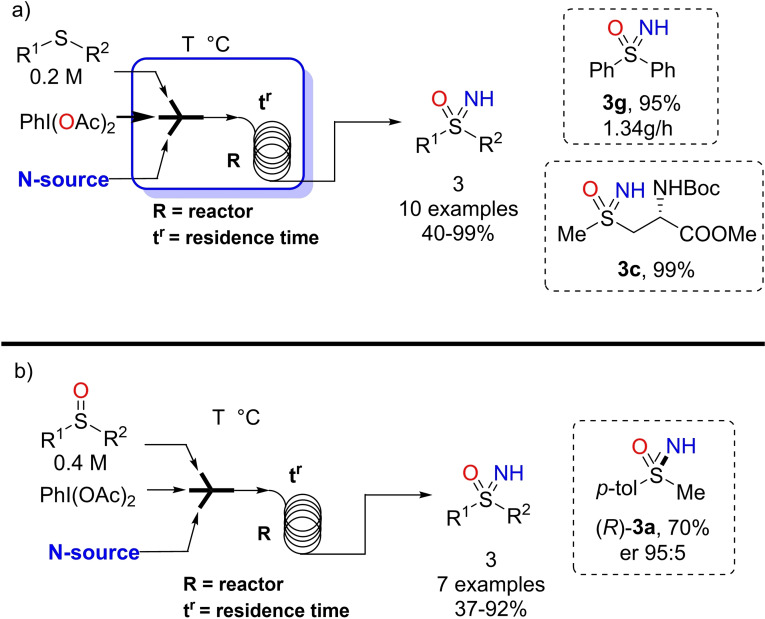
Mild synthesis of NH‐sulfoximines in flow devices.

Luisi optimized the one pot O‐ and NH‐transfer protocol on sulfides and the NH‐transfer to sulfoxides by using flow devices.^[87]^ The optimization study on methylphenyl sulfide was carried out into a Vapourtec R2 system equipped with a 10 mL PTFE reactor and 2 mL PTFE loops (Scheme 29, a). In order to avoid the risk of precipitation, an adapted concentration of 0.2 M of sulfide in methanol was employed. The initial screening of the solubility of nitrogen source and oxidant in different solvents was needed to avoid clogging. Under flow conditions, ammonium carbamate was difficult to handle, due to its high tendency to decompose, while ammonium carbonate dissolved slowly in methanol and the resulting solution needed to be filtered. Ammonium acetate and aqueous ammonia were found as the suitable ammonia sources. In the presence of 2 equivalents of PhI(OAc)_2_, 2 equivalents of NH_3(aq)_, with 15 minutes of residence time at 0 °C, the desired NH‐sulfoximine was obtained in 95 % yield. The use of sulfoxides as substrate required a concentration of 0.4 M. To manage the higher concentrations of PhI(OAc)_2_, and N‐source, a different flow set‐up, consisting in 10 mL PTFE coil reactor and syringe pumps, was realized (Scheme 29, b). The optimal flow conditions used 2 equivalents of PhI(OAc)_2_, 2 equivalents of N‐source (ammonium acetate or aqueous ammonia), at 0 °C with 30 minutes of residence time. In comparison to batch approach, the use of the flow technology allowed to reduce the equivalents of both oxidant and ammonia source. Moreover, the scope of the flow method was investigated considering the nature of the S‐substituent as well as the functional group tolerance. The continuous flow synthesis of biologically relevant methionine sulfoximine (MTO) and enantioenriched sulfoximine (*R*)‐**3** **a** was reported. It is worth mentioning that the flow protocol was tested in a long run continuous flow synthesis observing a productivity of 1.34 g/h for phenyl methyl sulfoximine **3** **g**.

## 4 Recent Developments in the Functionalization of NH‐Sulfoximines

The availability of new robust and effective methods to access NH‐sulfoximines boosted the development of methods for their functionalization. Recent advances in the field will be highlighted in this section, focusing on selected examples. In particular, the recently developed protocols for N‐sulfonylation, sulfenylation, phosphorylation, acylation, vinylation, arylation, cross‐coupling, and cyclization will be covered. Moreover, recent progresses in the use of NH‐sulfoximines for the synthesis of heterocycles and for the preparation of new hypervalent iodine reagents will be discussed.

### 4.1 N‐Sulfonylation, sulfenylation, and phosphorylation

**Scheme 30:**
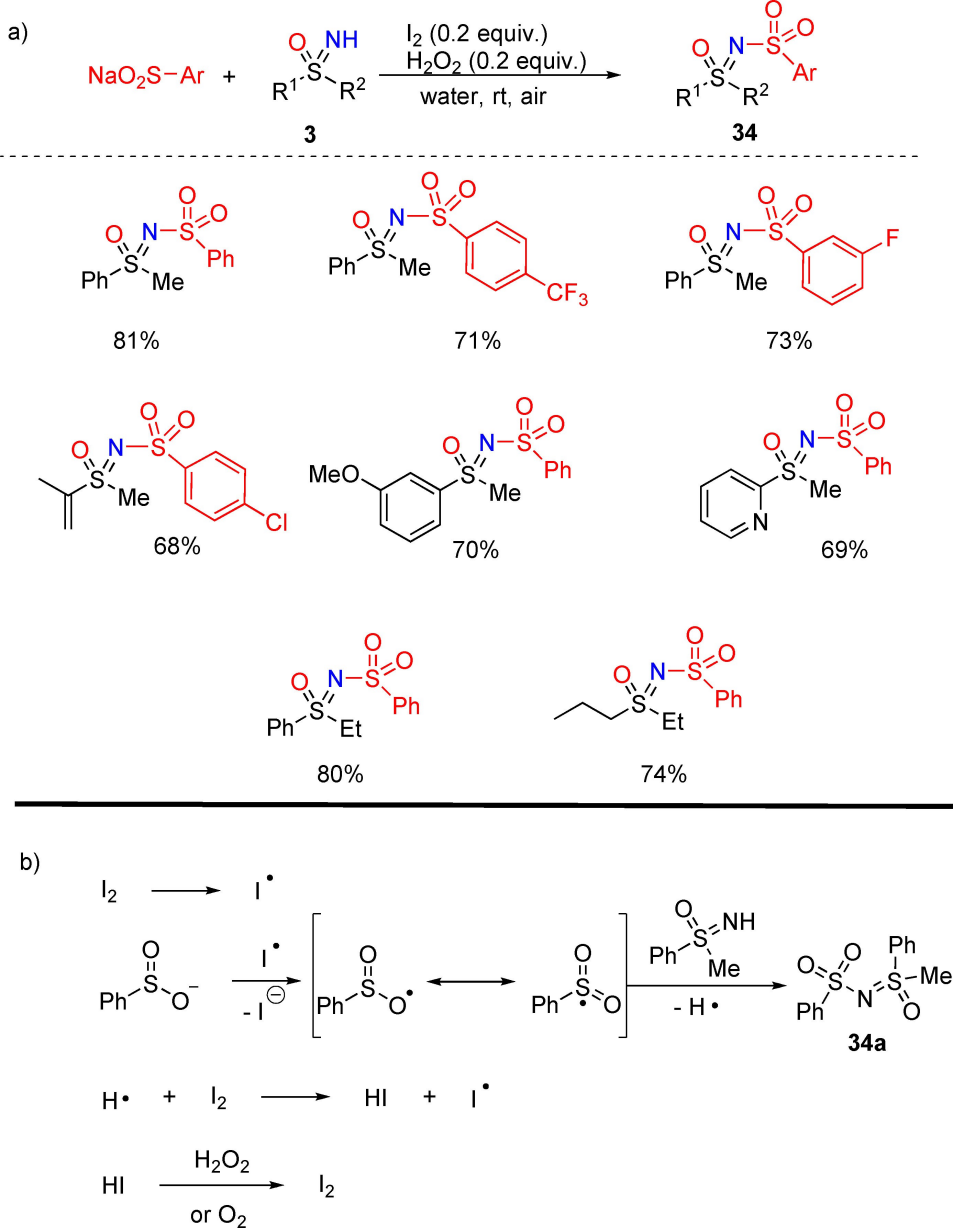
N‐sulfonylation of NH‐sulfoximines upon iodine catalysis.

The development of synthetic strategies for the preparation of N‐sulfonyl sulfoximines is desirable, as these compounds have been disclosed as efficient chiral auxilaries.^[15(b)]^ Zeng and coworkers described the synthesis of N‐sulfonyl sulfoximines **34** via oxidative N−S bond formation by coupling of NH‐sulfoximines and sodium alkyl‐sulfinates.^[88]^ The protocol required I_2_ (0.2 equiv.) as the catalyst, H_2_O_2_ as the oxidant in water at room temperature (Scheme 30, a). The reaction furnished good yields using varied aryl sulfonates coupled with substituted alkyl, aryl, and dialkyl sulfoximines **3**. The authors supposed that radical species might be involved in the process, as the addition of an excess of TEMPO, as the radical scavenger, inhibited the reaction. The proposed mechanism starts with the reaction of phenylsulfinate with radical iodine to form a S‐centered radical, which subsequently reacts with NH‐sulfoximine to give the desired N‐sulfonyl sulfoximine **34** **a** (Scheme 30, b). Molecular iodine (I_2_) is restored from HI by oxidation with H_2_O_2_ or molecular oxygen.

**Scheme 31:**
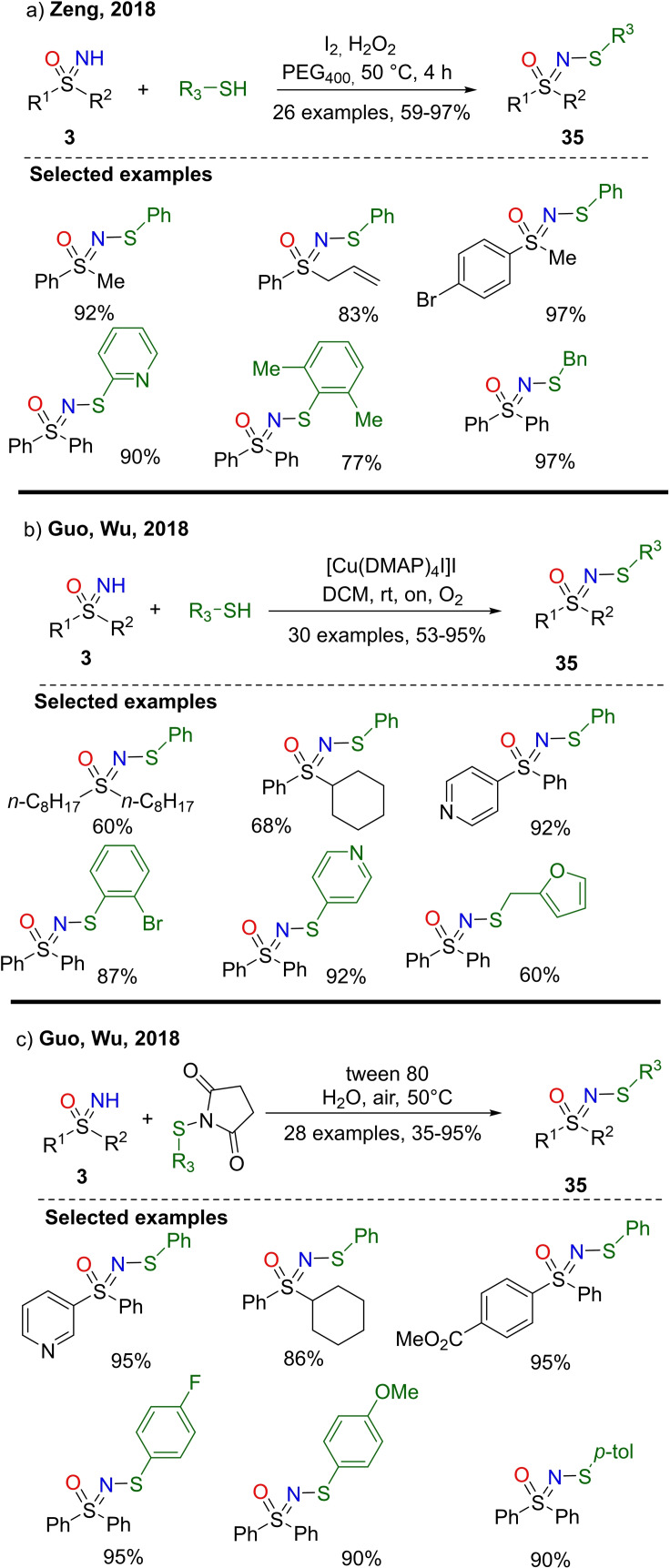
Recent N‐sulfenylation reactions.

In contrast to N‐sulfonylation, N‐sulfenylation of NH‐sulfoximine has been poorly explored, and the conventional routes had limitations due to the use of hazardous reagents. However, only recently, elegant and efficient methods have been developed. In 2018, Zeng and coworkers reported a metal‐free, iodine catalyzed N−H/S−H dehydrocoupling reaction between NH‐sulfoximines **3** and thiols to afford N‐sulfenylsulfoximines **35** (Scheme 31, a).^[89]^ The reaction occurred with high yields in the presence of I_2_ as the catalyst, and H_2_O_2_ as the oxidant in PEG400 at 50 °C. Non‐toxic reaction medium, high atom‐economy, and functional group tolerance characterized this methodology. Wu and Guo described a metal‐catalyzed synthesis of N‐sulfenylsulfoximines by reacting NH‐sulfoximines and thiophenols.^[90]^ In this process, the reaction proceeded with 2 equivalents of thiophenol and 20 mol% of [Cu(DMAP)_4_I]I as the catalyst at room temperature. A variety of NH‐sulfoximines and thiols were tested, and the methodology exhibited a very good functional group tolerance, providing moderate to good yields of the desired products **35** (Scheme 31, b). The same authors developed a sustainable preparation of N‐sulfenylsulfoximines **35** by reacting NH‐sulfoximines **3** and N‐(phenylthiol)succinimides in water (Scheme 31, c).^[91]^ The presence of the commercial additive tween 80 in the reaction media allowed the preparation of the desired products in good to excellent yields and the reaction exhibited a good functional group tolerance.

**Scheme 32:**
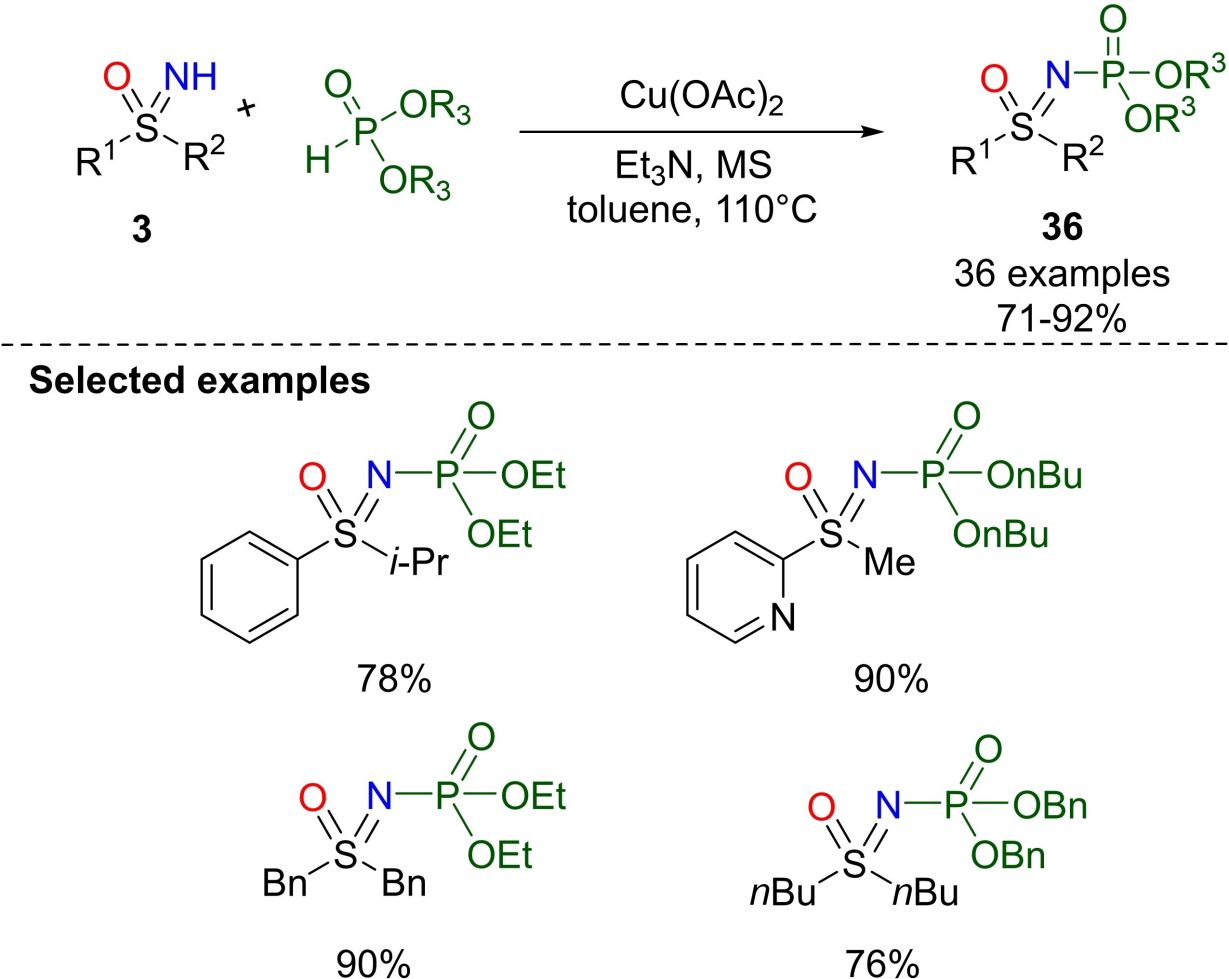
N−P coupling reaction of NH‐sulfoximines with dialkyl phosphites.

An efficient route to access N‐phosphorylated sulfoximines under mild conditions was recently reported by Kandasamy and coworkers.^[92]^ NH‐sulfoximines underwent N−P coupling with dialkyl phosphites in the presence of Cu(OAc)_2_ as the catalyst, triethylamine as the base in toluene at 110 °C, and in the presence of molecular sieves (Scheme 32). High yields of N‐phosphorylated sulfoximines **36** could be obtained from heteroaromatics and dialkyl sulfoximines **3**. Moreover, the reaction was not inhibited by the addition of a radical scavenger, suggesting that the reaction did not proceed by a radical pathway.

### 4.2 N‐Acylation

Over the last few decades, the renewed interest in N‐acylated sulfoximines, prompted several research groups to develop novel and efficient N‐aroylation strategies. In fact, N‐acylated sulfoximines have been recently used as directing groups for C−H bond activation,[^81^, ^93^] and introduced as a structural motif in bioactive pseudopeptides.^[94]^ Sulfoximine‐promoted C−H activation and annulation strategies have enabled the construction of interesting structural motif as π‐conjugated polycyclic amides,^[95]^ spiro‐isoquinolones,^[96]^ pyranoisoquinolines,^[97]^ and oxepino‐pyridines,^[98]^ among others.^[99]^ From a synthetic point of view, the most traditional approach for N‐acylation of sulfoximines involved the use of activated acyl chlorides.[^21^, ^70^, ^92^, ^100^]

**Scheme 33:**
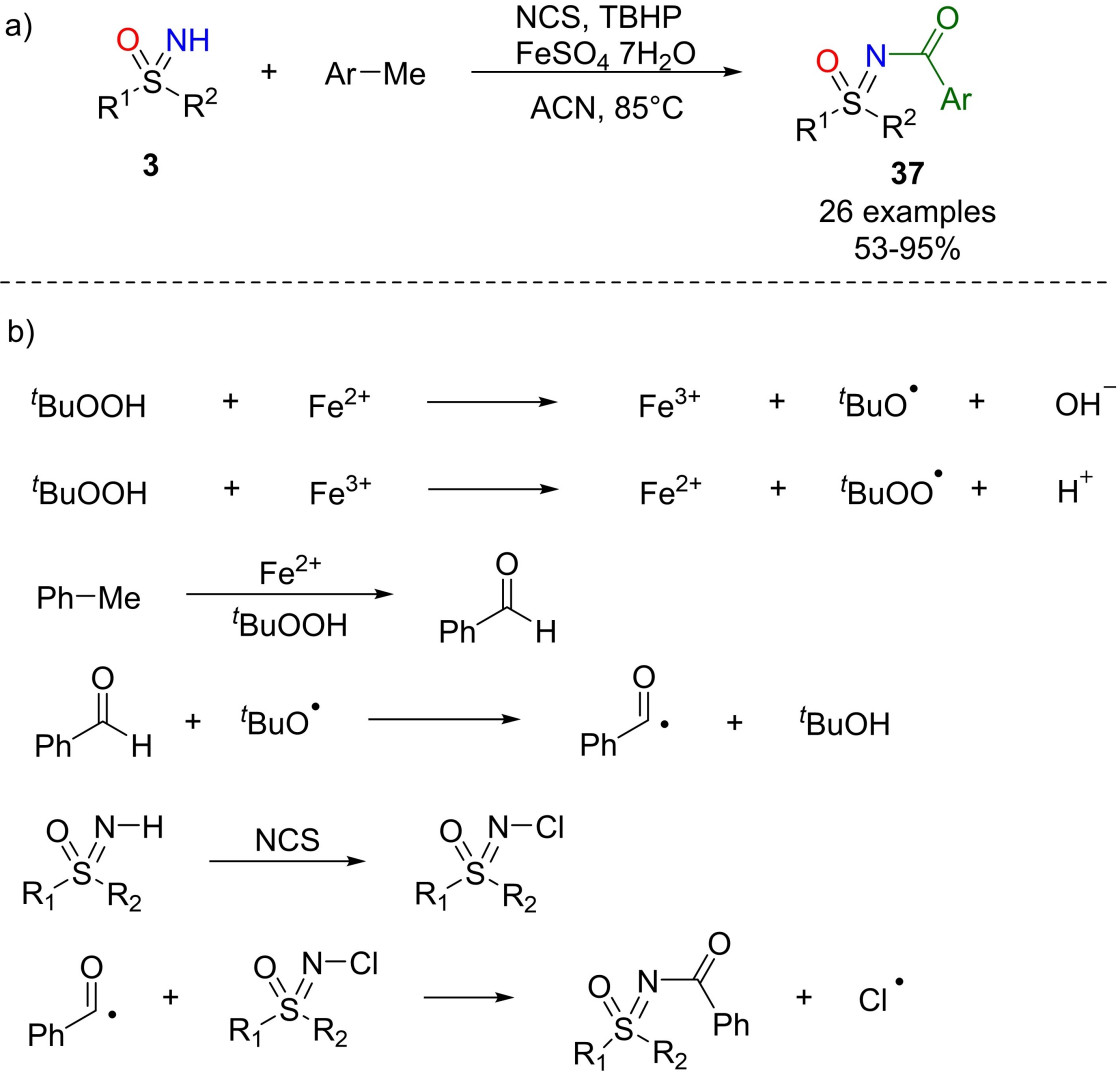
N‐aroylation of NH‐sulfoximines through iron catalysis.

In 2016, Sekar reported the synthesis of N‐aroylated sulfoximines from methylarenes as aroyl sources and NH‐sulfoximines under iron(II) catalysis.^[101]^ The optimal conditions required FeSO_4_•7H_2_O as the catalyst, TBHP as the oxidant, NCS in acetonitrile and a temperature of 85 °C (Scheme 33, a). Methylarenes bearing methyl, methoxy, and nitro groups, also in *ortho*‐position, gave the desired N‐aroylated sulfoximines **37** in good yields. No traces of the corresponding products were detected in the presence of methylfuran, methylthiophene, and methylpyridine. Moreover, the scope of the NH‐sulfoximines was investigated, leading to the N‐aroylated products in good to high yields. No product was detected running the reaction in the presence of radical scavengers, demonstrating the radical pathway of the process. Interestingly, the reaction occurred in the presence of N‐chlorosulfoximine, instead of sulfoximine and NCS, indicating its possible involvement in the reaction mechanism. According to the proposed mechanism, the sequence of events begins with the oxidation of toluene to benzaldehyde, the formation of aroyl radical from the latter aldehyde with Fe/TBHP followed by generation of amino radical from N‐chlorosulfoximine. Finally, the formation of the desired product is expected to arise from the reaction of aroyl and amino radicals as shown in Scheme 33 (b).

**Scheme 34:**
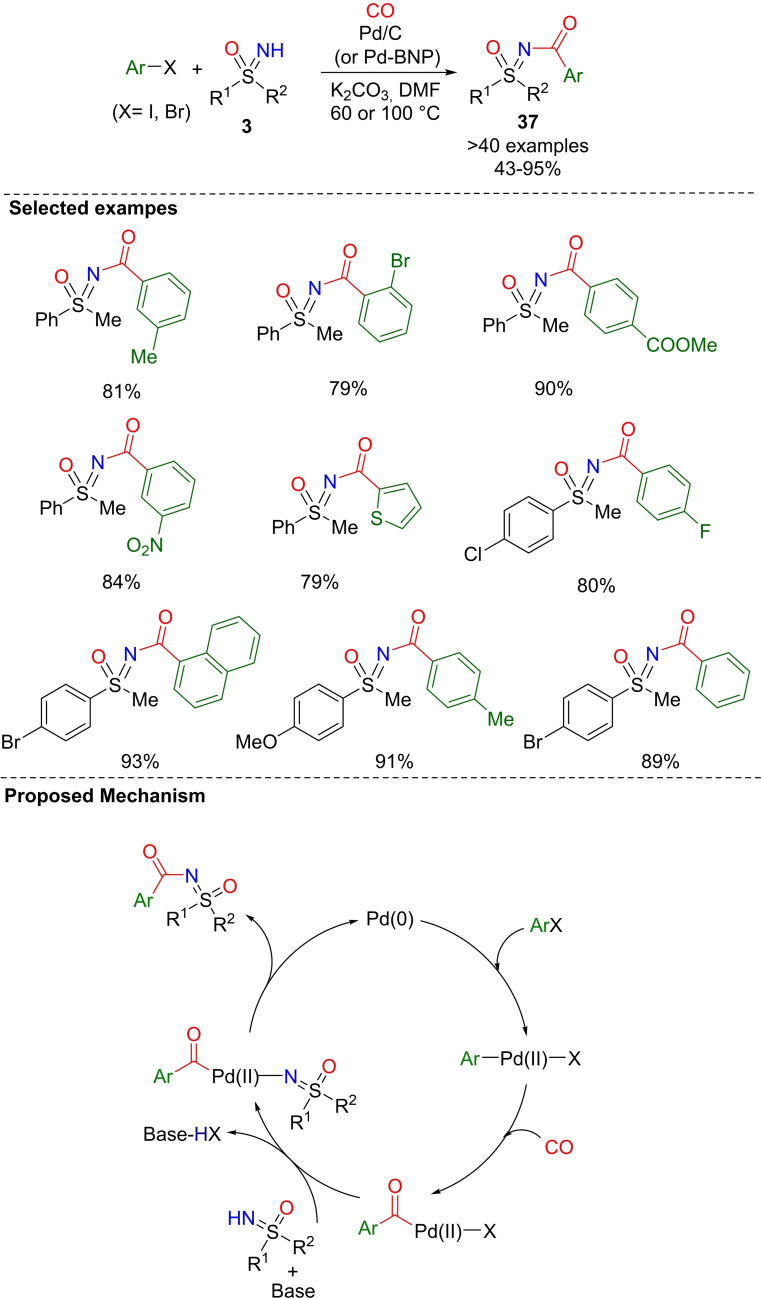
Sulfoximinocarbonylation of aryl halides upon Pd/C catalysis.

Another strategy for the N‐aroylation of sulfoximines from aryl iodides and bromides was recently reported by Sekar and coworkers.^[102]^ Two protocols were developed: one employed Pd/C catalyst (1 mol%), K_2_CO_3_ as the base and proceeds under CO atmosphere, using DMF as the solvent (Scheme 33). Alternatively, N‐aroylation was conducted using palladium nanoparticles (Pd‐BNP) as the catalyst, K_2_CO_3_ as the base, under CO atmosphere in DMF at 80 °C (Scheme 34).^[103]^ Several substituted iodoarenes and NH‐sulfoximines **3** were coupled delivering the desired N‐aroylsulfoximines **37** in good to excellent yields. The proposed mechanism involves the Pd(0) oxidative addition to the aryl halide, followed by CO insertion, nucleophilic attack of sulfoximine, and the final reductive elimination. Moreover, one of the main advantages of these procedures is represented by the recyclability, up to six times, of Pd/C or Pd‐BNP catalysts without significant loss of efficiency, and without leaching or residual metal contamination in the final product.

**Scheme 35:**
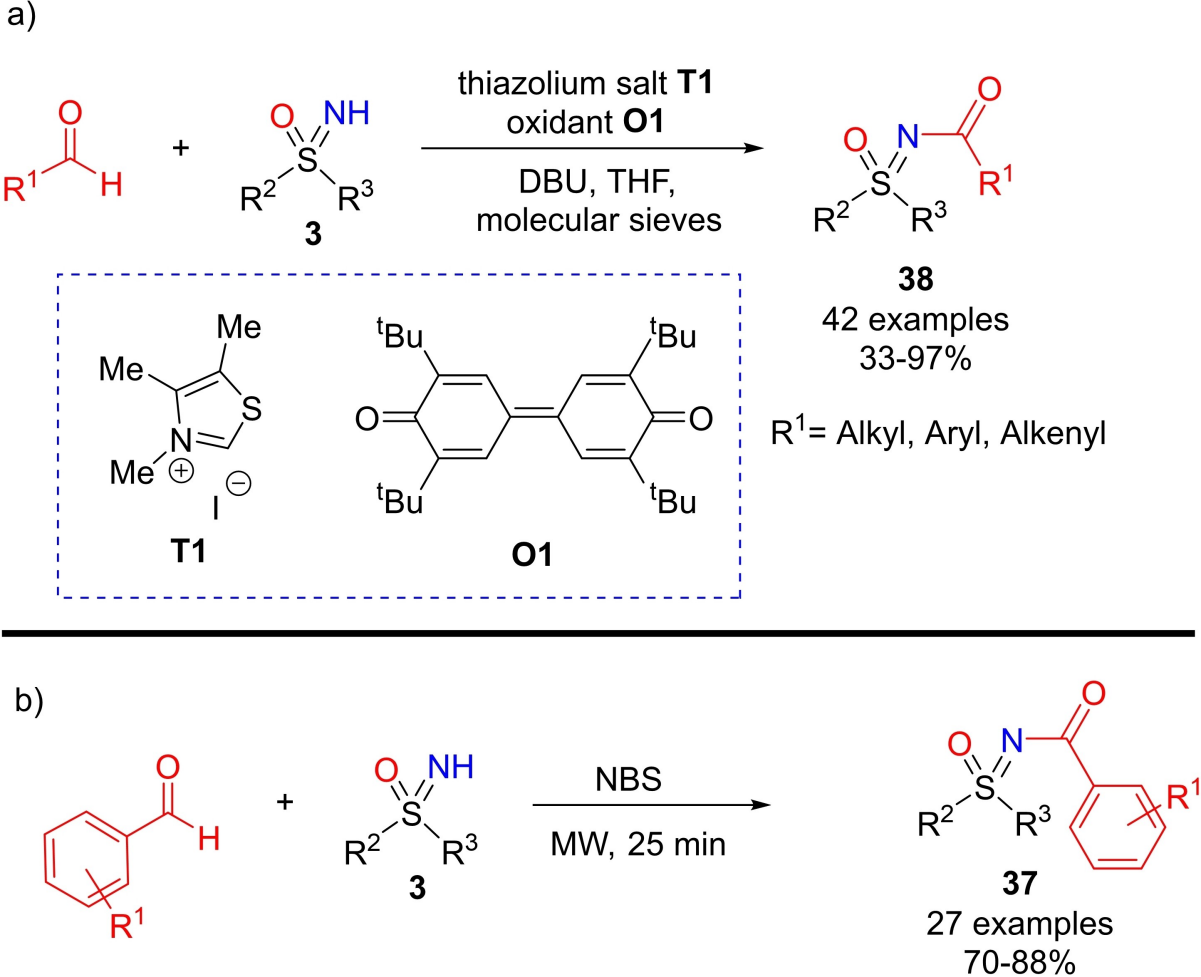
N‐Acylation of NH‐sulfoximines with aldehydes.

The direct acylation of NH‐sulfoximines can be also performed with aldehydes under N‐heterocyclic carbene (NHC)‐catalysis, as reported by Guin.^[104]^ Good to excellent yields of N‐acyl sulfoximines **38** were obtained using thiazolium salt **T1**, DBU as the base, bisquinone **O1** as the oxidant, in the presence of molecular sieves (Scheme 35, a). The reaction performed well using substituted NH‐sulfoximines and aromatic, heteroaromatic, aliphatic and α,β‐unsaturated aldehydes. The mechanism may involve the catalytic generation of a redox‐active acyl donor intermediate from aldehyde, which reacted with NH‐sulfoximine to furnish the expected N‐acyl derivative. Interestingly, the acylation reaction on NH‐sulfoximines with arylaldehydes can be otherwise performed upon microwave irradiation and in the presence of NBS, as recently reported by Naicker, Arvidsson and coworkers (Scheme 35, b).^[105]^

**Scheme 36:**
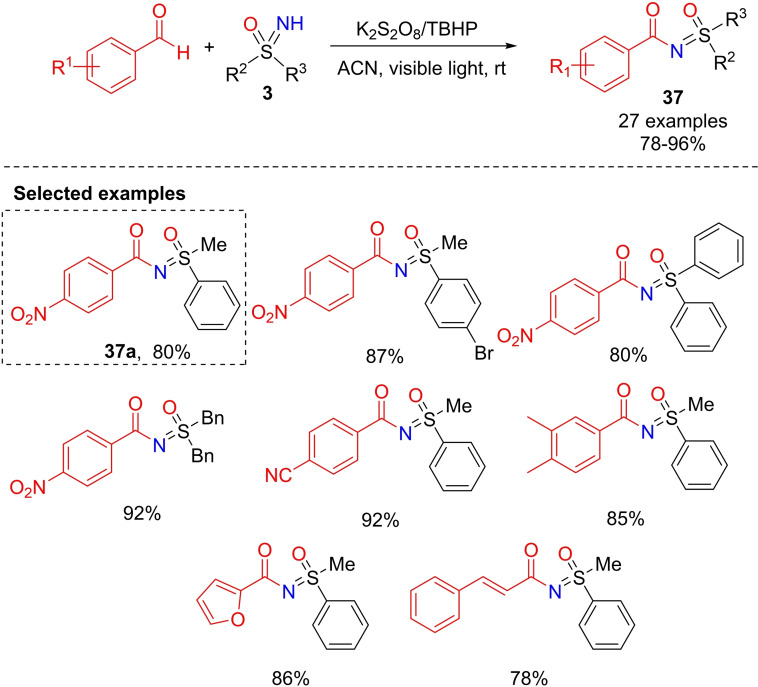
Visible light promoted N‐aroylation of NH‐sulfoximines.

A visible‐light promoted method for the synthesis of N‐aroylsulfoximines **37** from aldehydes has been developed by Zeng.^[106]^ First, S‐methyl‐S‐phenylsulfoximine and *p*‐nitrobenzaldehyde reacted in the presence of a mixture of oxidants TBHP/K_2_S_2_O_8_ under air at room temperature and upon irradiation with simulated sunlight (xenon arc lamp), affording the desired N‐aroylsulfoximine **37** **a** in 80 % yield (Scheme 36). The scope in aldehydes and NH‐sulfoximines was subsequently investigated, and the method demonstrated a good tolerance toward several functional groups. Moreover, no racemization occurred under the reaction conditions used for N‐acylation, preserving the chirality of enantiomerically enriched sulfoximines.

**Scheme 37:**
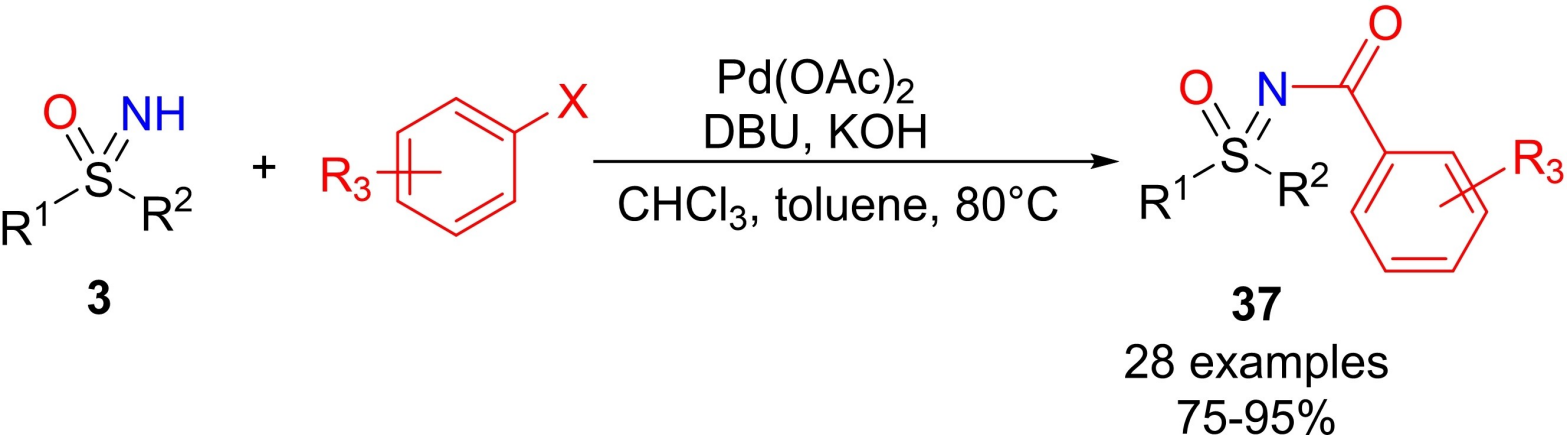
N‐aroylation of NH‐sulfoximines with aryl halides and chloroform as the CO source.

An alternative approach for the palladium‐catalyzed aroylation of aryl halides with sulfoximines has been reported by Yuan and Kumar, and employed chloroform as the CO precursor.^[107]^ The reaction required Pd(OAc)_2_ as the catalyst, DBU, KOH, CHCl_3_ for the in situ generation of CO, and proceeds in toluene at 80 °C (Scheme 37). The scope of the reaction was investigated by varying NH‐sulfoximines and aryl halides, obtaining the desired products **37** usually in good yields. Reasonably, the reaction mechanism may follow the typical palladium‐catalyzed carbonylative coupling pathway.

**Scheme 38:**
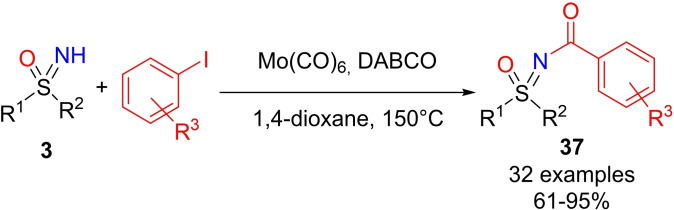
N‐acylsulfoximines preparation via Mo(CO)_6_ catalyzed imino‐carbonylation.

An interesting method for accessing a wide range of N‐acyl sulfoximines, has been developed by Kandasamy.^[108]^ The imino‐carbonylative acylation of NH‐sulfoximines occurred with aryl iodides in the presence of Mo(CO)_6_ as the CO donor, 1,4‐diazabicyclo[2.2.2]octane (DBCO) in 1,4 dioxane at 150 °C. The method showed good tolerance of functional groups, furnishing N‐acylsulfoximines **37** in 61–95 % yield (Scheme 38).

**Scheme 39:**
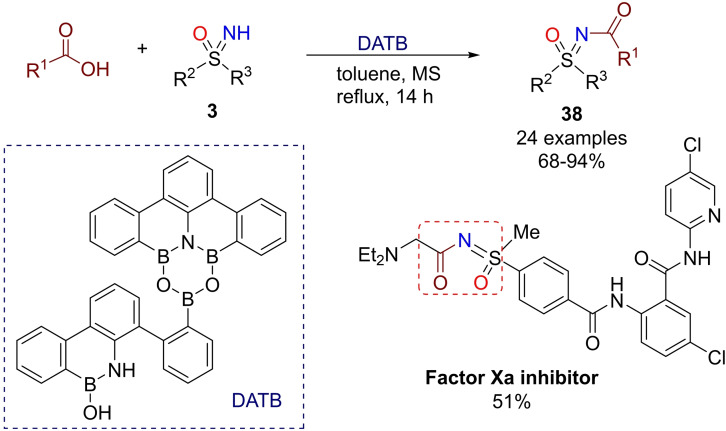
N‐Acylation of sulfoximines with carboxylic acids.

In 2017, Kumagai reported the direct acylation of NH‐sulfoximines with carboxylic acids.^[109]^ An efficient screening of different parameters led to the identification of 1,3‐dioxa‐5‐aza‐2,4,6‐triborinane (DATB) as the best catalyst for this transformation (Scheme 39). The method allowed the preparation of N‐acylsulfoximines **38** in high yields, by employing several functionalized carboxylic acids. In addition, the method was applied to a favorable synthesis of a biologically active compound (Factor Xa inhibitor).

**Scheme 40:**
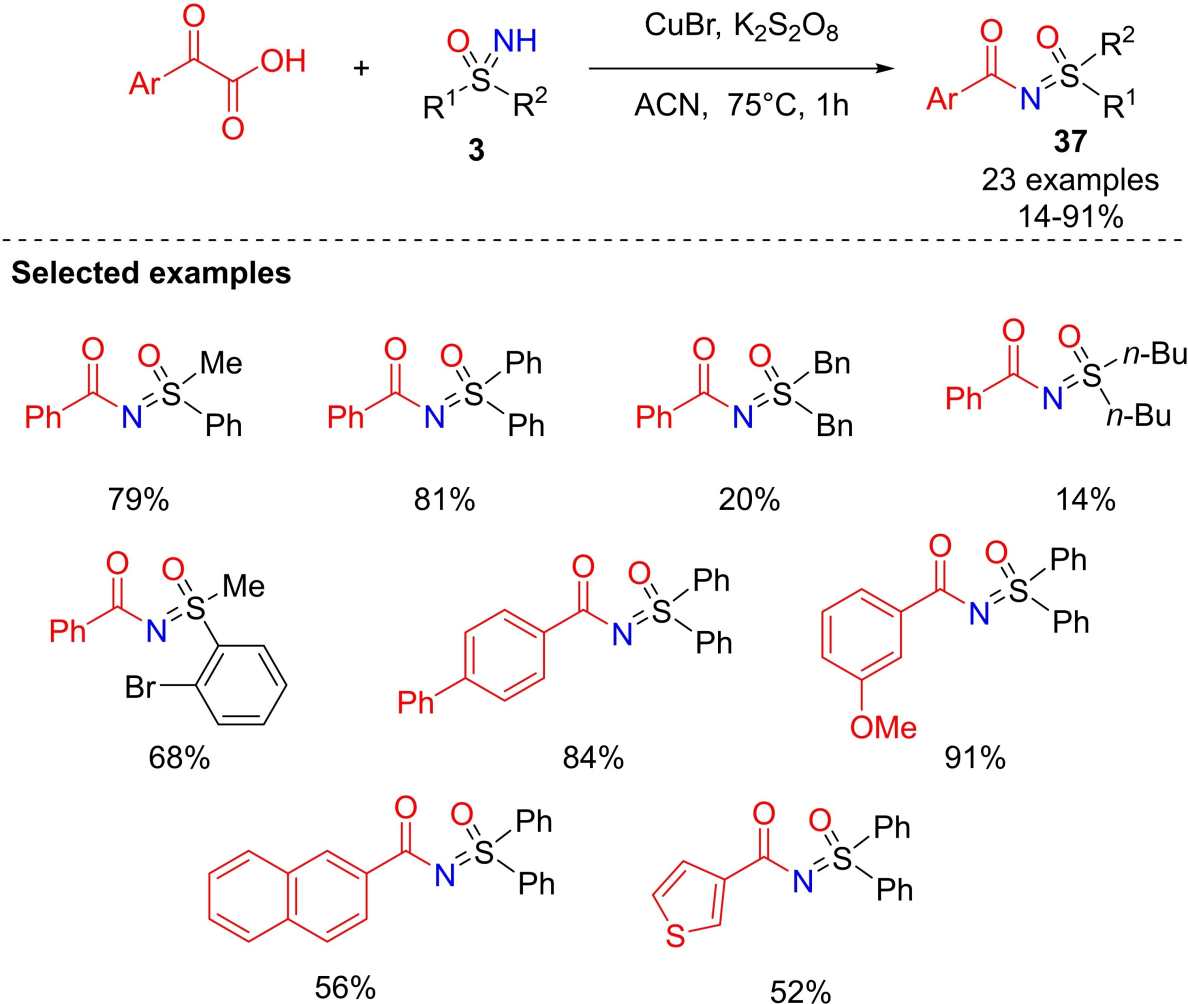
N‐Aroyl sulfoximines from α‐ketoacids and NH‐sulfoximines by copper‐catalysis.

Yotphan reported a copper‐catalyzed aroylation of NH‐sulfoximines by using α‐ketoacids as arylating agents.^[110]^ This strategy involved aryl and heteroarylglyoxylic acid derivatives and NH‐sulfoximines in the presence of potassium persulfate (K_2_S_2_O_8_) as the oxidant in acetonitrile at 75 °C (Scheme 40). The reaction performed very well, returning several functionalized N‐acylated sulfoximines **37** in good to excellent yields. Mechanistic investigations in the presence of radical scavengers such as 2,6‐bis(1,1‐dimethylethyl)‐4‐methyl‐phenol (BHT), TEMPO, and hydroquinone, supported the involvement in this process of radical species. Interestingly, the Cu(II) catalysis was mandatory for a successful decarboxylative coupling.

**Scheme 41:**
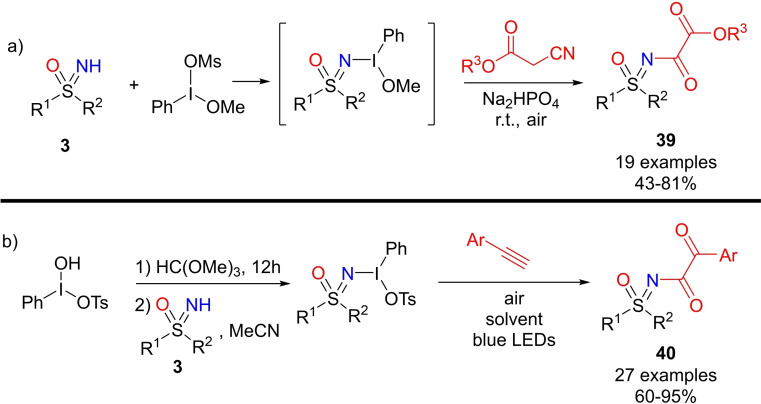
Synthesis of N‐α‐ketoacylated and N‐α‐ketoesters sulfoximines.

Bolm reported recently the synthesis of sulfoximines bearing a α‐ketoester functionality at the nitrogen atom.^[111]^ The strategy involved a one‐pot reaction of NH‐sulfoximines and methoxy(mesyloxy)iodobenzene to afford hypervalent iodine reagents that underwent reaction with cyanoacetates, furnishing the desired products **39** in good yields (Scheme 41, a). The scope of the reaction was thoroughly explored by structural variation at both sulfoximines and cyanoacetates. In general, the protocol was effective with several aryl and alkyl sulfoximines, and the authors developed a sustainable visible light‐promoted synthesis of N‐α‐ketoacylated sulfoximines **40** under air.^[112]^ In this case, methoxy(phenyl)‐λ^3^‐iodanyl methanesulfonate was employed as the sulfoximidoyl donor, and reacted with arylalkynes to afford the desired products in very good yields (Scheme 41, b).

**Scheme 42:**
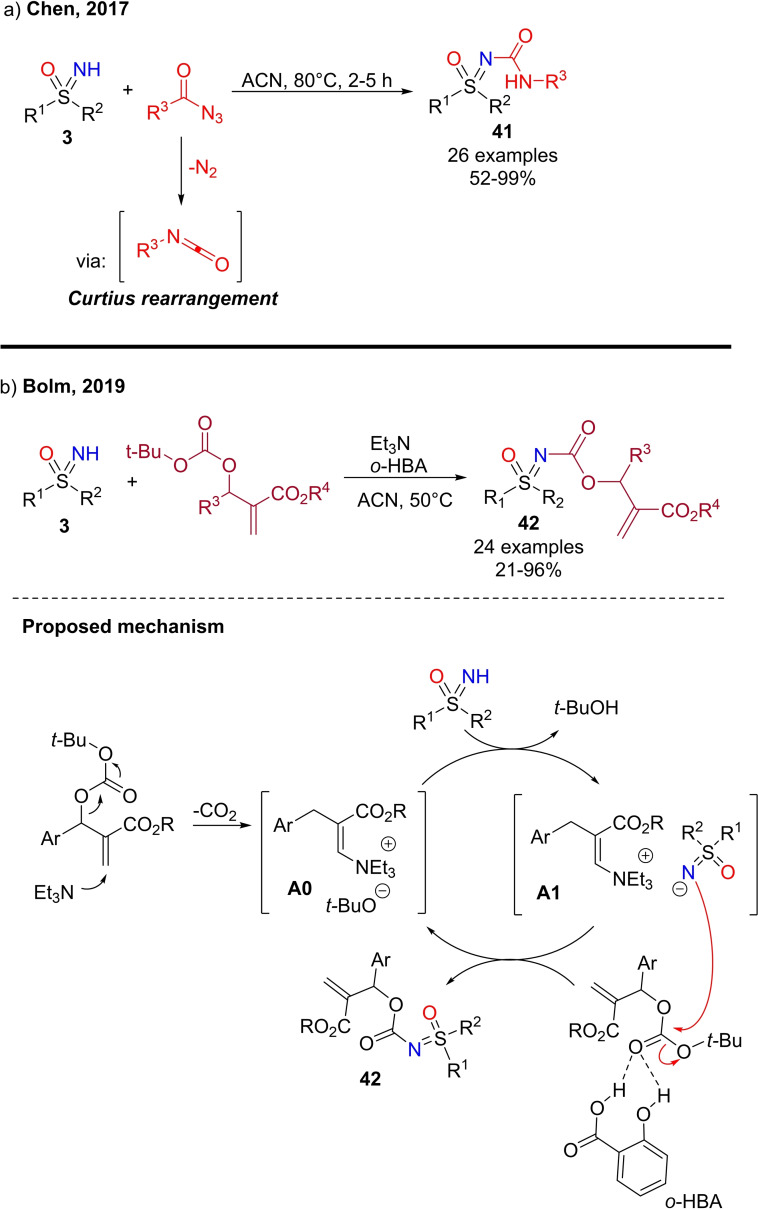
Synthesis of sulfonimidoyl ureas and carbamates.

A different approach for the N‐functionalization of NH‐sulfoximines, was developed by Chen and coworkers.^[113]^ The authors reported a Curtius rearrangement‐based approach for the synthesis of sulfonimidoyl ureas **41** under metal‐free conditions (Scheme 42, a). The reaction enabled a straightforward preparation of sulfonimidoyl ureas by mixing NH‐sulfoximines **3** and acyl azides in acetonitrile at 80 °C. In a similar way, Bolm disclosed the synthesis of sulfoximidoyl carbamates **42** through the reaction of NH‐sulfoximines **3** with Morita‐Baylis‐Hillman carbonates in the presence of triethylamine and *o*‐hydroxybenzoic acid in acetonitrile at 50 °C (Scheme 42, b).^[114]^ The proposed mechanism involves the base promoted decarboxylation of the starting carbonate followed by the deprotonation of NH sulfoximine from *tert*‐butylate leading to the ionic couple **A1** (scheme 42, b). The anionic sulfoximine is supposed to attack a second molecule of carbonate, activated by the coordination of *o*‐HBA, affording the product and restoring the initial *tert*‐butylate ammonium salt **A0**.

### 4.3 Preparation of N‐halogen sulfoximines

**Scheme 43:**
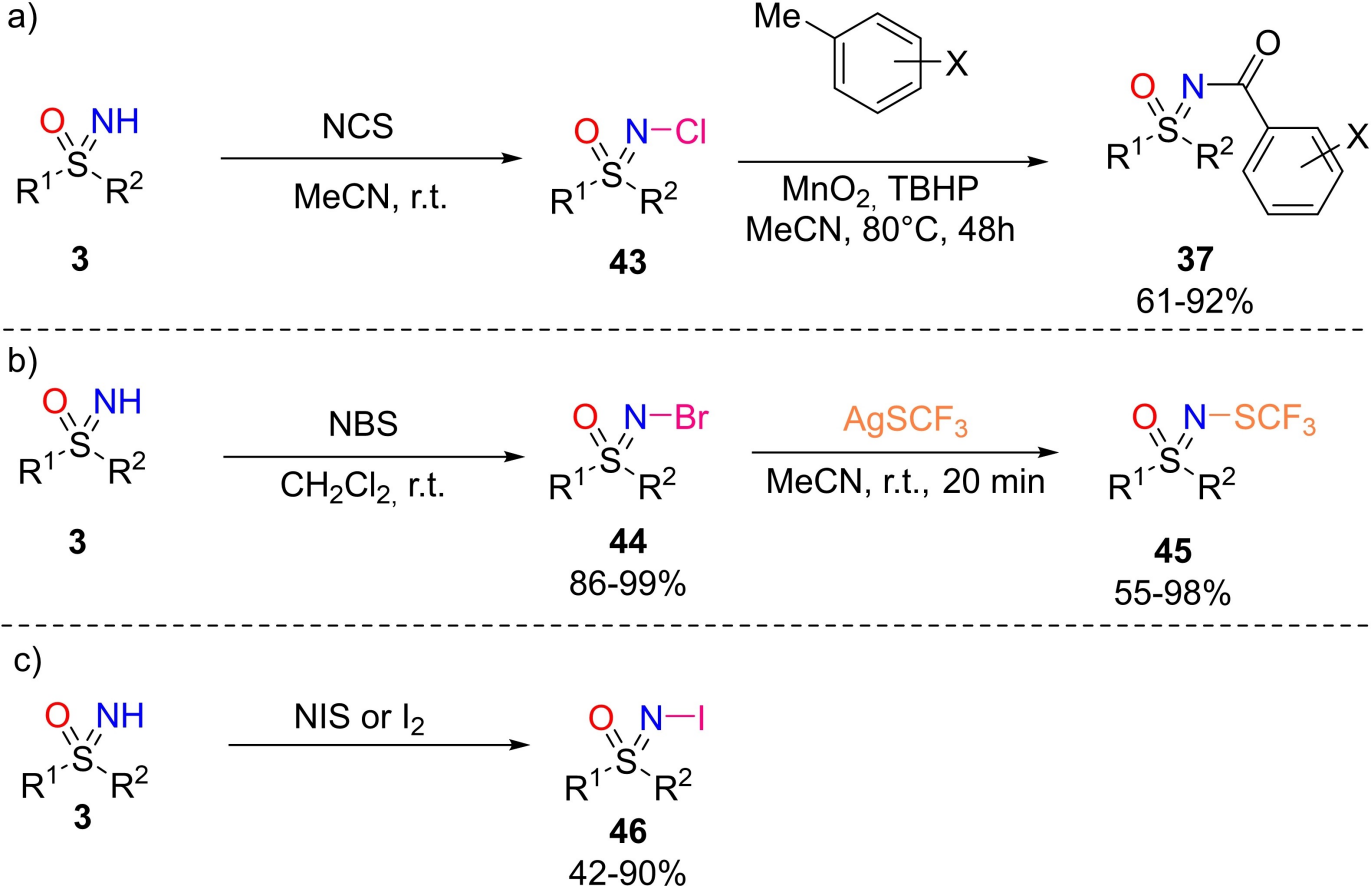
Synthesis of N‐halogen sulfoximines from NH‐sulfoximines and related transformations.

N‐halogen sulfoximines are useful reagents for functionalizations of the nitrogen atom. Some efficient strategies for the synthesis of N‐halogen sulfoximines have been recently developed. In 2014, Bolm and coworkers described the preparation of N‐chloro sulfoximines **43** from NH‐sulfoximines **3** upon treatment with N‐chloro succinimide (Scheme 43, a).^[115]^ Similarly, N‐bromination can be performed with N‐bromo succinimide (Scheme 43, b), ^[116]^ and N‐iodo sulfoximines **46** can be prepared with N‐iodo succinimide or molecular iodine (Scheme 43, c).^[117]^

Moreover, the transformation of N‐chloro and N‐bromo sulfoximines towards N‐aroylated sulfoximines **37** and N‐trifluoromethylthiolated sulfoximines **45**, respectively, have been reported (Scheme 43, a and b).

**Scheme 44:**
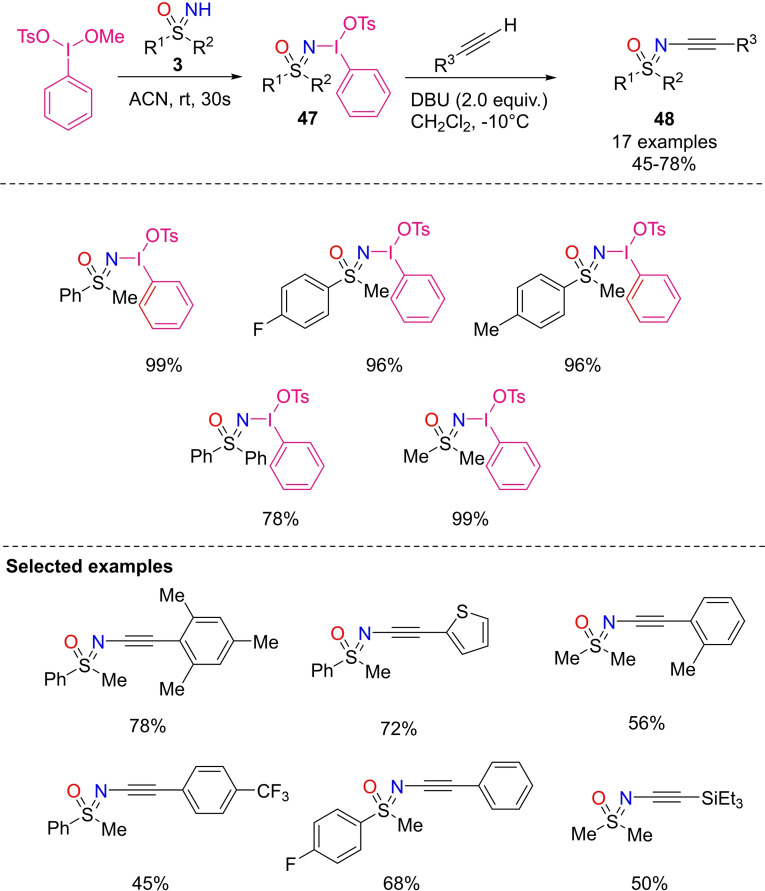
Sulfoximidoyl hypervalent iodine(III) reagents synthesis.

The preparation of novel hypervalent iodine (III) reagents through ligand exchange of NH‐sulfoximines with methoxy(tosyloxy)iodobenzene (MTIB) in acetonitrile has been recently documented by Bolm and coworkers.^[18(g),28,118]^ The iodonium salts **47** were achieved in excellent yields by reacting different NH‐sulfoximines (Scheme 44). These compounds exhibit satisfactory stability at room temperature in the solid state, and in solution over an extended reaction time. Moreover, the hypervalent iodine (III) reagents **47** have been subsequently transformed with alkynes in the presence of DBU, affording N‐ alkynylated sulfoximines **48** in moderate to good yields.

**Scheme 45:**
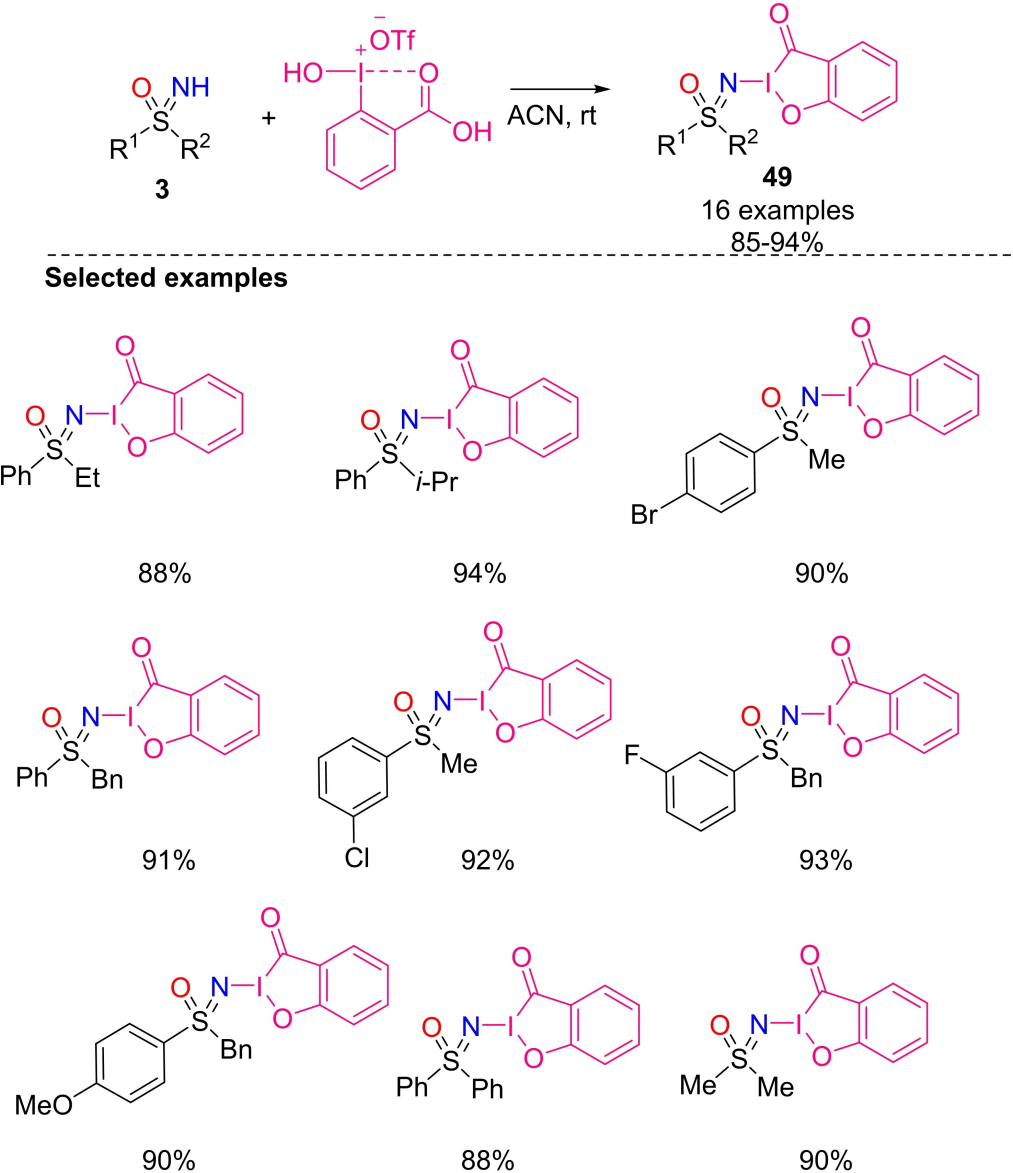
Synthesis of 1‐sulfoximidoyl‐1,2‐benziodoxoles.

In 2017, Bolm et al. reported the preparation of 1‐sulfoximidoyl‐1,2‐benziodoxoles **49** from NH‐sulfoximines **3** and benziodoxole triflate.^[119]^ The reaction proceeds in acetonitrile with 3 equivalents of sulfoximines at room temperature, and several S,S‐dialkyl, S,S‐diaryl and S‐alkyl‐S‐aryl sulfoximines have been successfully transformed in high yields (Scheme 45). Interestingly, the hypervalent iodine reagents exhibit a satisfactory stability. In fact, no decomposition was observed storing a solid sample hypervalent iodine (III) reagents at room temperature for five days and at 50 °C for 12 h. Similarly, the products remained stable when dissolved in halogenated and alcoholic deuterated solvents, in deuterated DMSO and heavy water.

**Scheme 46:**
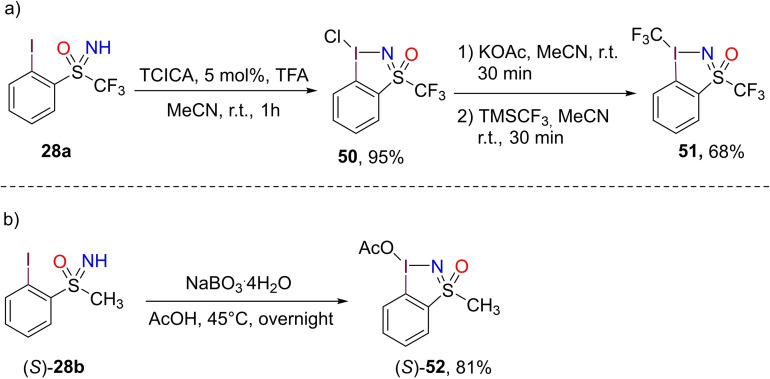
Preparation of incorporated sulfoximine hypervalent iodine reagents.

Recently, some examples of sulfoximines incorporated into hypervalent iodine reagents have been reported. In 2019 Togni and Magnier described the synthesis of hypervalent iodosulfoximine reagent **51** from S‐2‐iodophenyl‐S‐trifluorimethyl NH‐sulfoximine **28** **a** (Scheme 46, a).^[120]^ The transformation proceeds in three steps through an isolable chloroiodane **50** which could be crystalized as enantiopure form (*S*)‐**50**. Notably, hypervalent reagent **51** acts as an efficient trifluormethyl transfer reagent. In a similar fashion, Wirth reported the synthesis of optically active hypervalent iodine reagent (*S*)‐**52** by reacting (*S*)‐S‐2‐iodophenyl‐S‐methyl NH‐sulfoximine **28** **b** with sodium perborate (Scheme 45, b).^[121]^

### 4.4 N‐β‐Fluoroalkylation

**Scheme 47:**
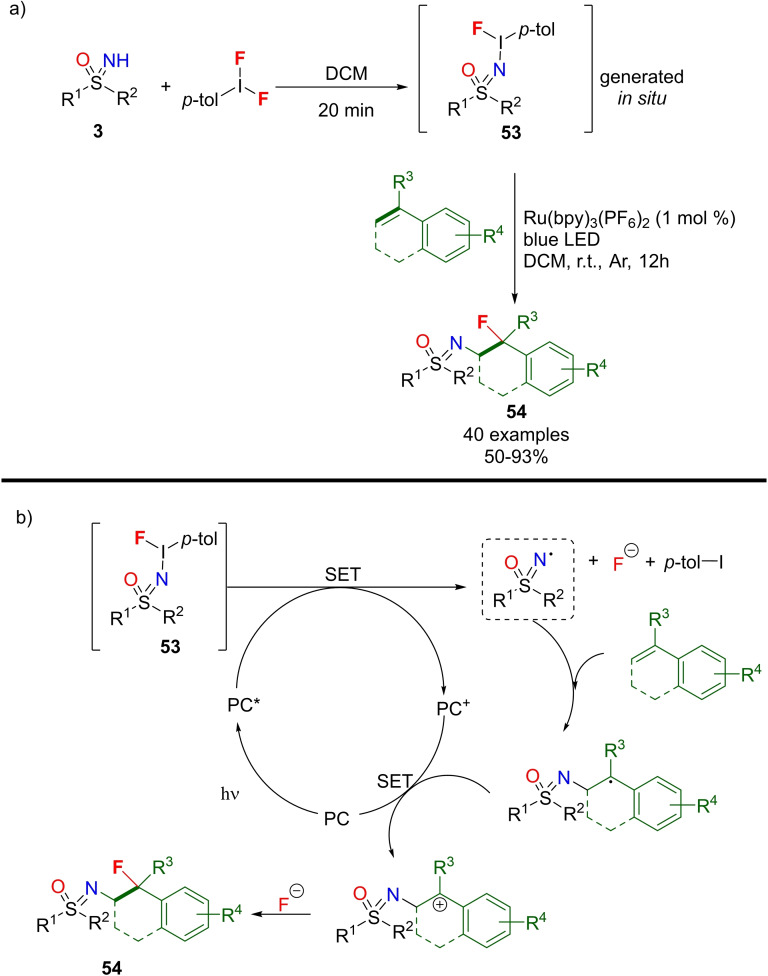
Ru‐catalyzed coupling of hypervalent iodine reagents with styrenes towards monofluoroalkylated sulfoximines.

Very recently, Bolm reported the in situ preparation of fluorinated sulfonimidoyl hypervalent iodine (III) reagents **53**, that reacted under photocatalytic conditions with styrenes to form N‐fluoroalkyl sulfoximines (Scheme 47, a).^[122]^ Diverse N‐fluoroalkyl sulfoximines **54** were prepared with high yields and regioselectivity under mild reaction conditions. The optimized one‐pot protocol used a ruthenium photocatalyst, and the scope of the reaction was widely explored by using several functionalized NH‐sulfoximines **3** and styrene derivatives (Scheme 47, a). The proposed mechanism involved the in situ generation of **53** which underwent N−I bond cleavage by a single electron transfer (SET) operated by the excited photocatalyst (PC*) (Scheme 47, b). Subsequently, the N‐centered sulfoximidoyl radical undergoes the regioselective addition to the double bond of the styrene reagent, forming a benzylic radical. Further oxidation, promoted by the photocatalyst (PC+), leads to the corresponding benzyl cation able to react with fluorine anion to furnish the final product, regenerating the ground state of the photocatalyst (PC).

### 4.5 N‐Arylation

**Scheme 48:**
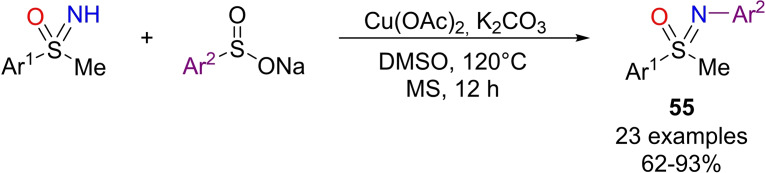
Arylation of NH‐sulfoximines by sodium arylsulfinates under Cu(II) catalysis.

The importance of N‐aryl sulfoximines and their derivatives relies in their use as potent chiral ligands.^[16]^ Several methodologies for the N‐arylation of NH‐sulfoximines have been reported in the last decade. In particular, this N‐functionalization can be achieved using different arylating agents as aryl halides,^[123]^ aryl triflates,^[124]^ aryl boronic acids,^[125]^ aryl siloxanes,^[126]^ diaryl iodonium salts,^[127]^ and arynes.^[128]^ An and Zhang reported a general method for the N‐arylation of NH‐sulfoximines using sodium arylsulfinates as efficient arylating agent.^[129]^ The optimal reaction conditions used Cu(OAc)_2_ as inexpensive catalyst, K_2_CO_3_ as the base in DMSO at 120 °C (Scheme 48). The protocol was applied to several aryl NH‐sulfoximines **3** and arylsulfinates combinations, affording the desired N‐arylated sulfoximines **55** in good to excellent yields. Interestingly, the reaction proceeds with the same efficiency under both O_2_ or Ar atmosphere, and the yield is not affected by the presence of TEMPO, demonstrating that the reaction is unlikely to proceed through a radical pathway.

**Scheme 49:**
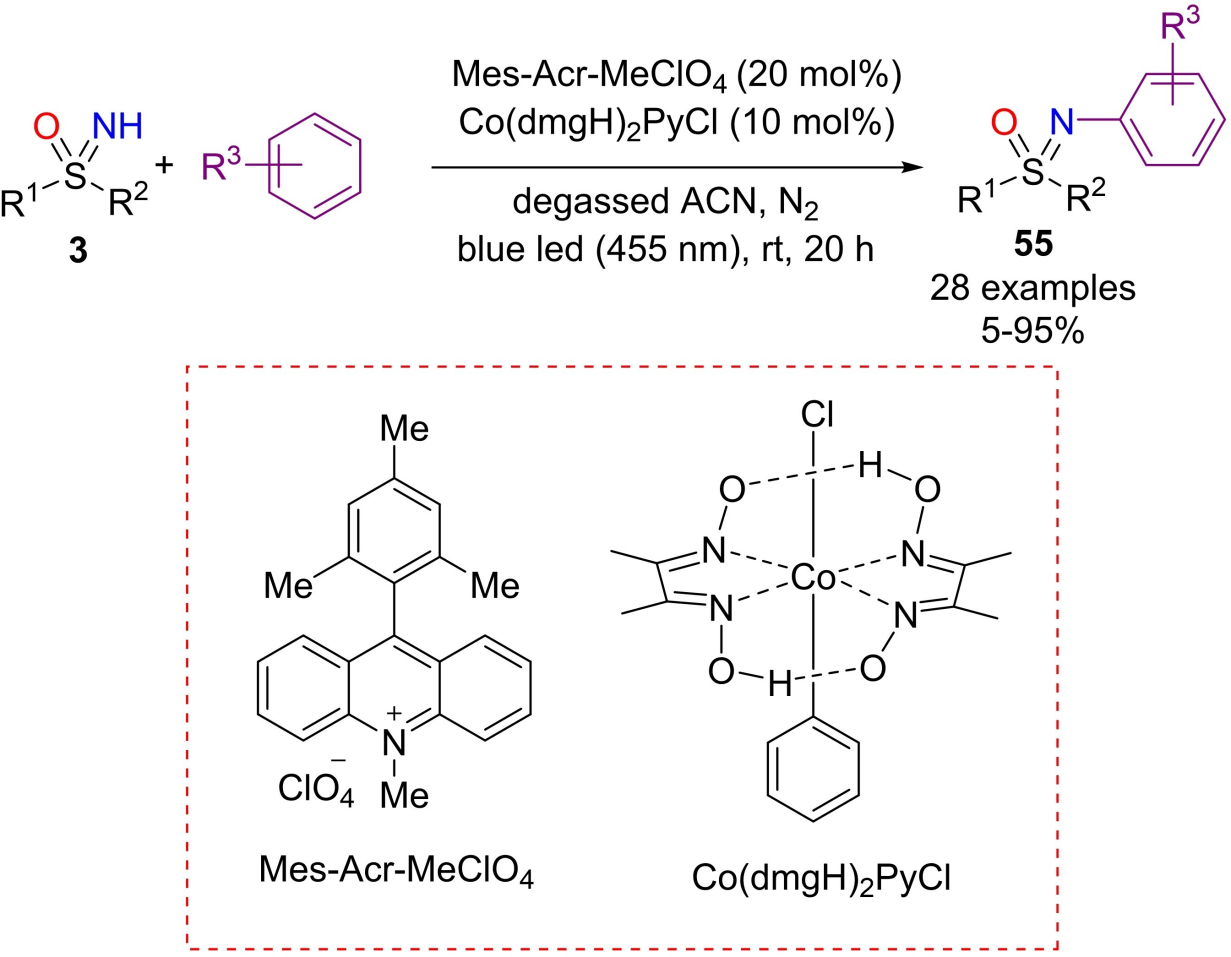
N‐arylation of NH‐sulfoximines by visible‐light oxidative photoredox catalysis.

König and Wimmerer developed the N‐arylation of NH‐sulfoximines with electron‐rich arenes under visible‐light oxidative photoredox catalysis.^[130]^ The reaction proceeds with 9‐mesityl‐10‐methylacridinium perchlorate as the organic photocatalyst, Co(dmgH)_2_PyCl as catalyst in degassed acetonitrile under N_2_ atmosphere and upon irradiation with blue light at 455 nm for 20 h at 25 °C (Scheme 49). A series of mono‐ and multi‐alkylated and halogenated arenes reacted with a broad range of aromatic and aliphatic electron‐rich and electron‐poor NH‐sulfoximines **3** with satisfactory yields. Moreover, the mechanistic investigation showed that both arenes and NH‐sulfoximines were photo‐oxidized to their corresponding radical intermediates, that underwent radical‐radical cross‐coupling reactions, leading to N‐arylated sulfoximines **55**.

**Scheme 50:**
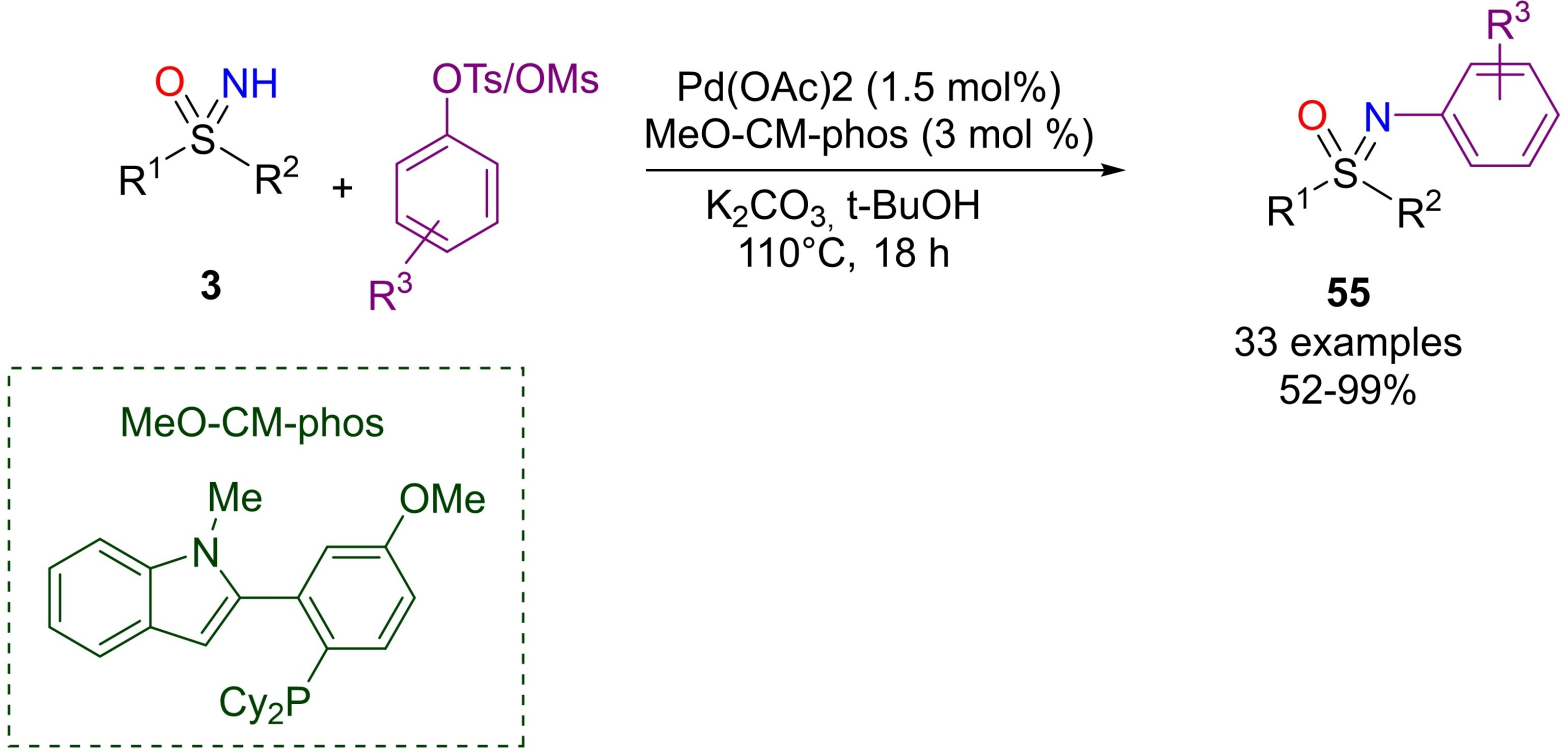
Preparation of N‐arylated sulfoximines by Pd‐catalysis.

In 2018, Kwong reported a palladium catalyzed N‐arylation of NH‐sulfoximines by using aryl sulfonates.^[131]^ The reaction involves Pd(OAc)_2_ as the catalyst, MeO‐CM‐phos as the ligand, K_2_CO_3_ as the base in *t‐*BuOH as the solvent (Scheme 50). Several aryl and alkenyl tosylates or mesylates were found to be suitable partners, and the reaction tolerated several functional groups as sulfoximine substituents giving N‐aroylated sulfoximines **55** in moderate to excellent yield.

**Scheme 51:**
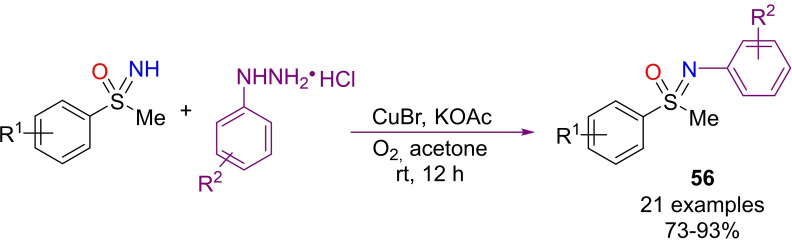
Copper‐catalyzed synthesis of N‐aryl sulfoximines with arylhydrazines.

An and Dong, developed a N‐arylation method that involved the use of arylhydrazine hydrochlorides under copper(I) catalysis.^[132]^ The strategy requires CuBr as the catalyst, KOAc as the base, acetone as the solvent, under O_2_ atmosphere (Scheme 51). Under optimized conditions, several S‐methyl‐S‐tolylsulfoximines could be N‐arylated furnishing products **56** in good yields. Moreover, a wide array of *ortho*‐, *meta*‐ and *para*‐substituted arylhydrazines with electron‐donating or withdrawing groups were compatible with this method. Mechanistic experiments suggested a radical pathway for this N‐arylation process.

**Scheme 52:**
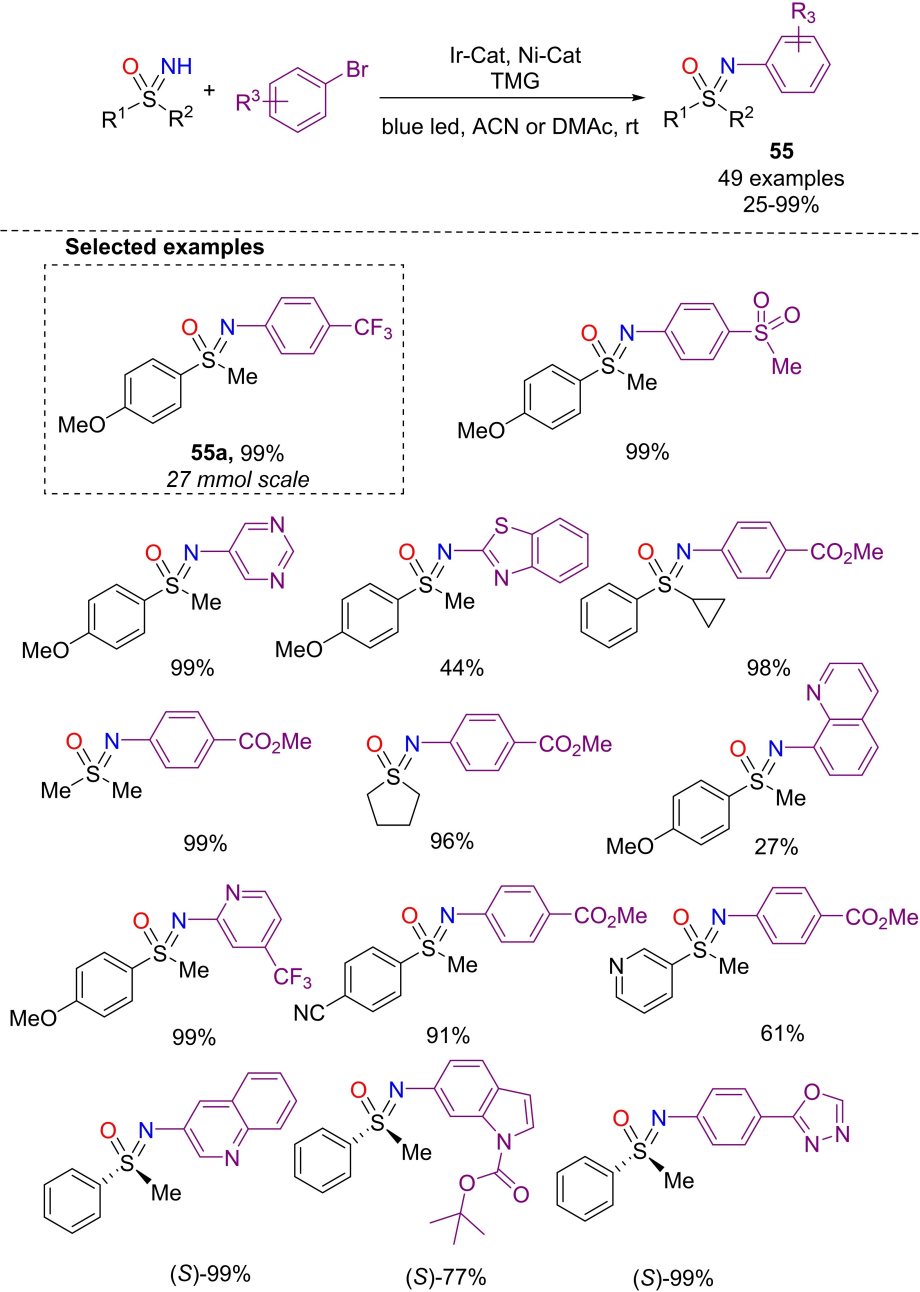
N‐Arylation of NH‐sulfoximines by dual Ir−Ni photocatalysis.

Very recently, König and Wimmer developed the N‐arylation of sulfoximines via dual nickel photocatalysis.^[133]^ The optimized protocol used an iridium photocatalyst ([Ir‐(ppy)_2_(dtbbpy)]PF_6_), NiBr_2_ as the second metal catalyst, and dtbbpy as ligand, TMG (1,1,3,3‐tetramethylguanidine) as the base, and irradiation at 455 nm (Scheme 52). Bromo arenes bearing different functional groups such as thioethers, amides, carbamates, as well as brominated pyrimidines, pyrazines, and quinolines were competent reaction partners, affording the desired products **55** in moderate to excellent yields. Alkyl as well as aryl NH‐sulfoximines **3** were found to be suitable for this N‐arylation reaction. No racemization was observed when the reaction was performed on enantiopure NH‐sulfoximines. Moreover, a scalability test in a custom‐made reactor was carried out on a preparative scale of 27 mmol, obtaining sulfoximine **55** **a** without any loss of yield.

**Scheme 53:**
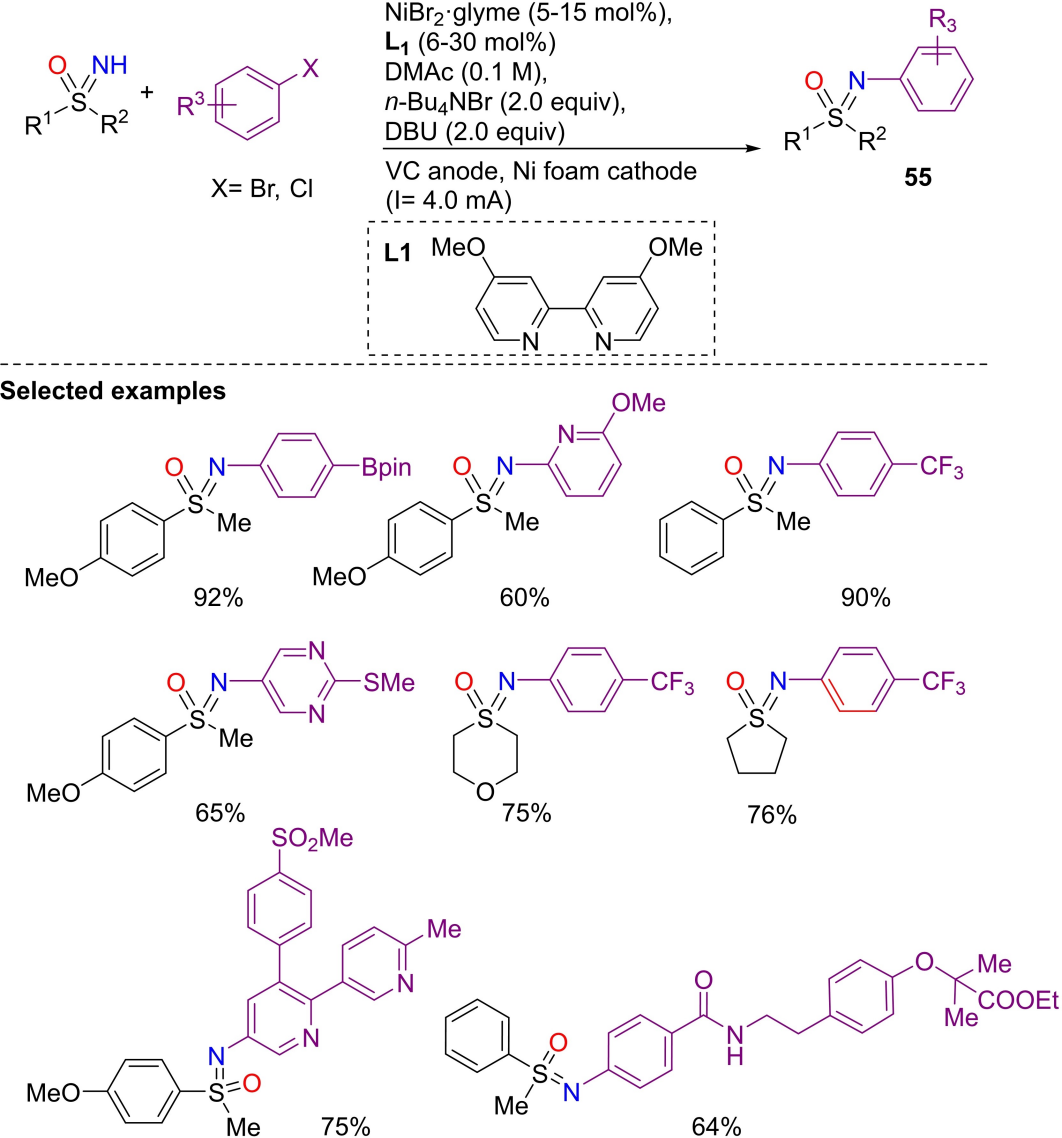
Ni‐catalyzed N‐arylation of NH‐sulfoximines via paired electrolysis.

The nickel‐catalyzed N‐arylation of NH‐sulfoximines with aryl halides via paired electrolysis has been reported recently by Mey and co‐workers.^[134]^ The reaction proceeds with aryl bromides and chlorides, and affords the products **55** in good to excellent yields (Scheme 53). Moreover, the mild reaction conditions are compatible with various functional groups, and the protocol is reported to be robust and operationally simple. In fact, several pharmaceutical agents have been transformed, enabling the preparation of the corresponding sulfoximines, and giving examples of efficient late stage functionalization reaction on complex substrates.

**Scheme 54:**
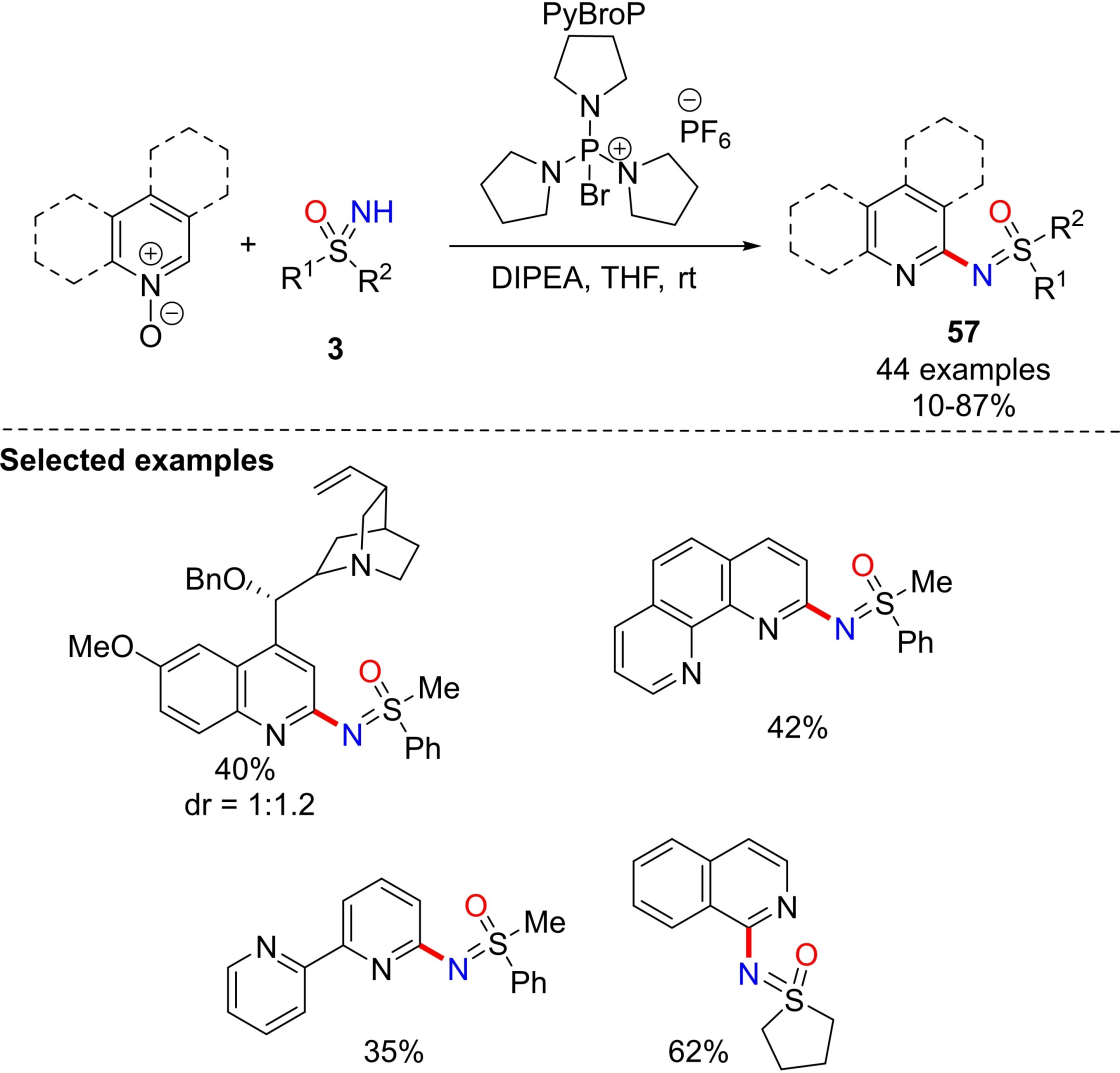
C−N coupling reaction for the sulfoximination of azine N‐oxides.

In 2016, Singh and co‐workers developed a sulfoximination of electron‐deficient heteroarenes.^[135]^ The strategy involves the use of isoquinoline‐N‐oxide and different NH‐sulfoximines in the presence of PyBroP (bromo tripyrrolidinophosphonium hexafluorophosphate) as the N−O bond activating agent, and diisopropylethylamine (DIPEA) as the base (Scheme 54).^[136]^ Good to high yields of corresponding N‐arylated products **57** were obtained using several substituted sulfoximines. This reaction is also efficient using various quinolines and pyridines, as well as with 1,10‐phenanthroline, 2,2’‐bipyridine, and quinine. In addition, the reaction with chiral optically active sulfoximines afforded the corresponding products with high stereocontrol (ee >99 %).

**Scheme 55:**
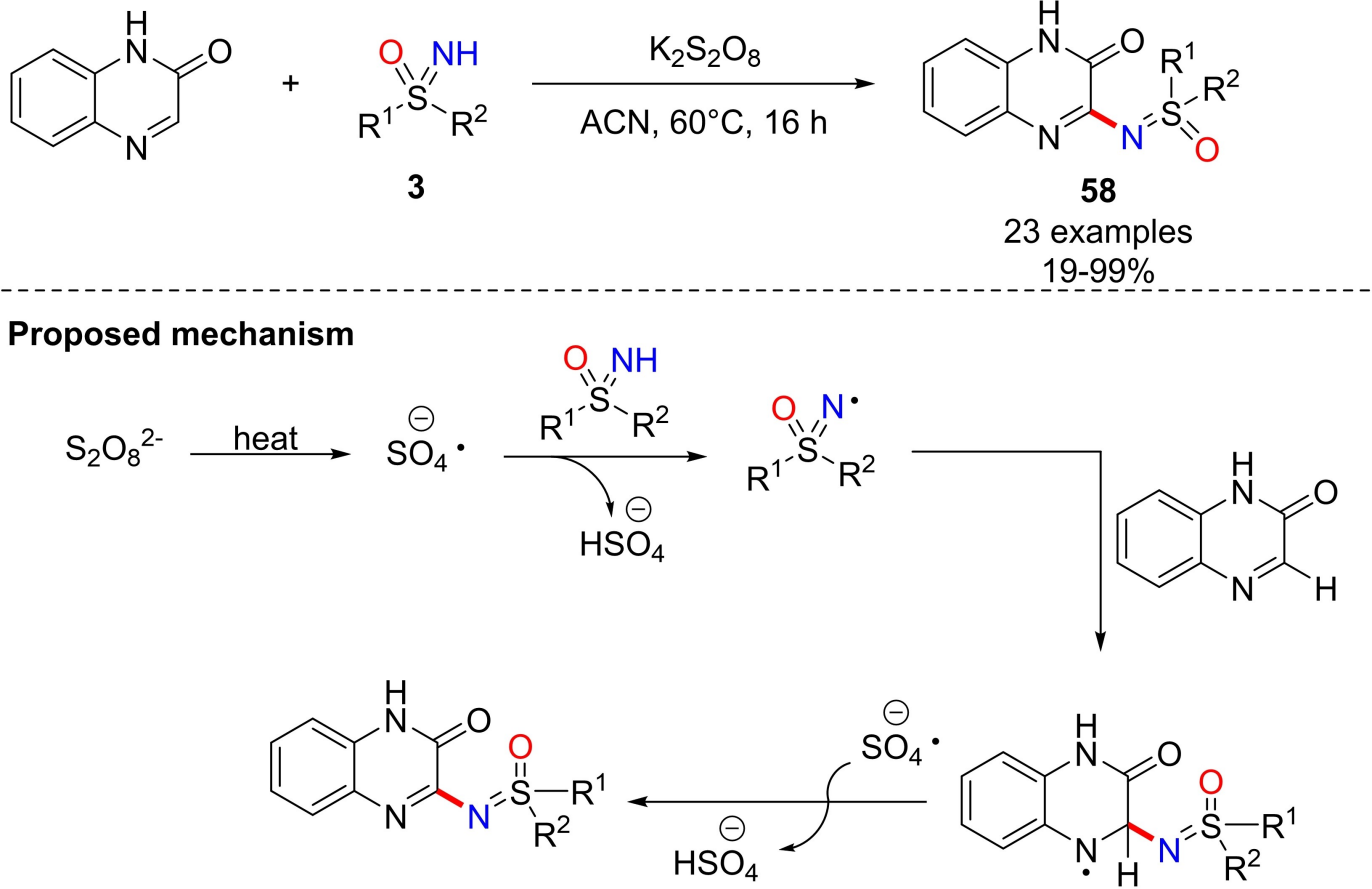
C−N bond formation from quinoxalinones and NH‐sulfoximines.

In 2018, Yotphan developed a methodology for the direct installation of the sulfoximine group at C3 position of quinoxalinone substrates.^[137]^ The method required the use of 1 equiv. of quinoxalinone, 2 equiv. of NH‐sulfoximine, K_2_S_2_O_8_ as the oxidant in acetonitrile at 60 °C (Scheme 55). The coupling products **58** were prepared in moderate to high yields, and preliminary studies on the reaction mechanism suggested a radical pathway.

**Scheme 56:**
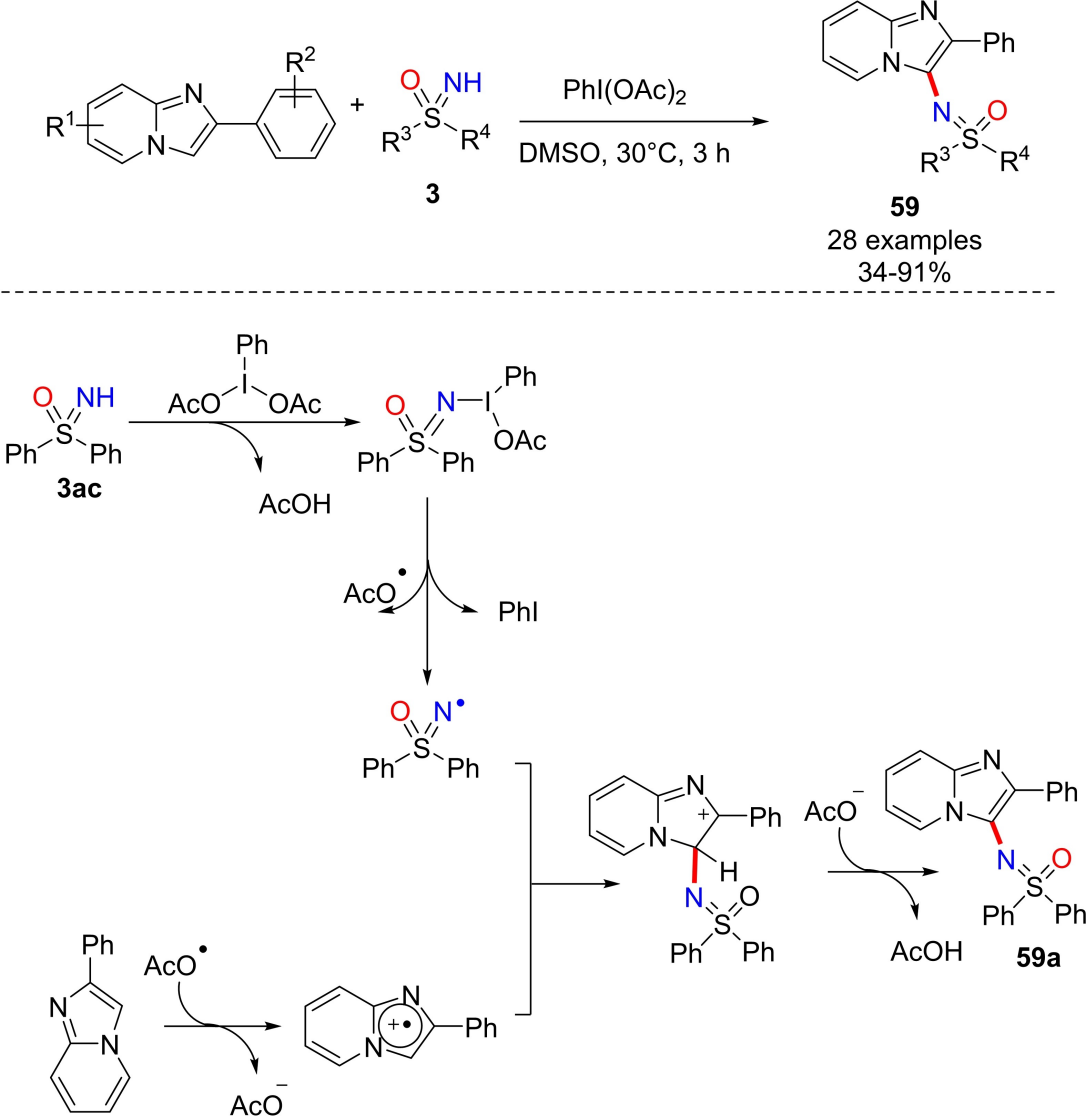
Sulfoximination of imidazopyridines by PhI(OAc)_2_.

Due to the increasing interest in imidazo[1,2‐a]pyridines, a structural unit found in many natural and pharmaceutical products,^[138]^ Wu disclosed an oxidative strategy for the C−H sulfoximination of imidazopyridines.^[139]^ The reaction occurred in the presence of functionalized imidazopyridines and NH‐sulfoximines, using PhI(OAc)_2_ in DMSO at 30 °C for 3 h and afforded the desired products **59** in poor to high yield (Scheme 56). The reaction mechanism is supposed to involve a radical pathway as described for the preparation of compound **59** **a** from NH‐sulfoximine **3** **ac** (Scheme 56).

**Scheme 57:**
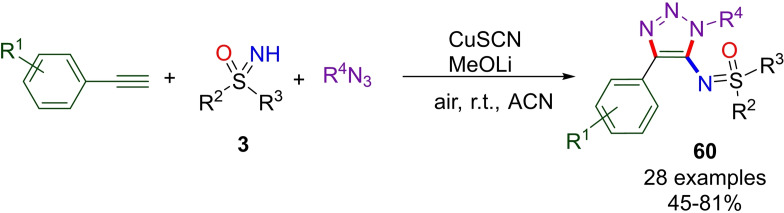
Three‐component synthesis of trisubstituted triazolyl sulfoximines.

Multicomponent reactions represent desirable strategies in organic chemistry, due to their atom economy, multiple‐bond forming efficiency, and the utilization of generally available starting materials. On this path, Song and Xu developed a three‐component reaction which employed NH‐sulfoximines **3** with alkynes, and azides for the direct synthesis of trisubstituted triazolyl sulfoximines **60** (Scheme 57).^[140]^ The transformation can be achieved under air and requires CuSCN as the catalyst and MeOLi as the base. The scope of the reaction was explored, highlighting that the electronic properties of the sulfoximine moiety have no significant effect on the reaction yield. On the contrary, electron rich and unsubstituted aryl acetyenes are generally best performing substrates. In addition, satisfactory yields were observed with a broad variety of benzyl azides bearing different functional groups.

### 4.6 Cyclization reactions

**Scheme 58:**
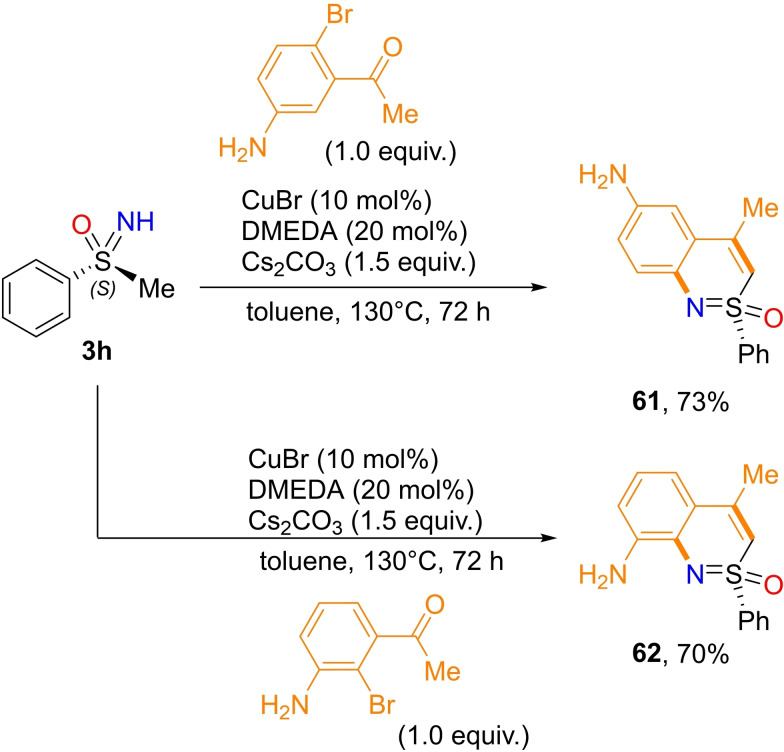
Synthesis of 1,2‐benzothiazines.

NH‐sulfoximines can undergo several inter‐ and intramolecular reactions leading to heterocyclic scaffolds. Most of the intramolecular transformations that allow the preparation of endocyclic S−N heterocycles involve the formation of both a new C−C bond, via C−H activation of S‐aryl sulfoximines, and N−C bond. As a result, the S‐oxides of 1,2‐benzothiazines, dihydro isothiazoles, tetrahydro‐1,2‐thiazines, 1,2‐benzothiazepines, 1,2,4‐thiadiazines and benzoisothiazoles are accessible from NH‐sulfoximines. Moreover, five, six and seven‐membered endocyclic sulfoximines can be afforded through various inter‐ and intramolecular cyclization reactions. In 2015, Bolm and coworkers disclosed the preparation of optically active 1,2‐benzothiazines **61** and **62** from (*S*)‐S‐methyl‐S‐phenylsulfoximine **3** **h** and brominated 3‐aminobenzophenones (Scheme 58). The reaction requires copper (I) bromide, 1,2‐dimethylethylenediamine and cesium carbonate, and affords the products **61** and **62** in good yield.^[141]^

**Scheme 59:**
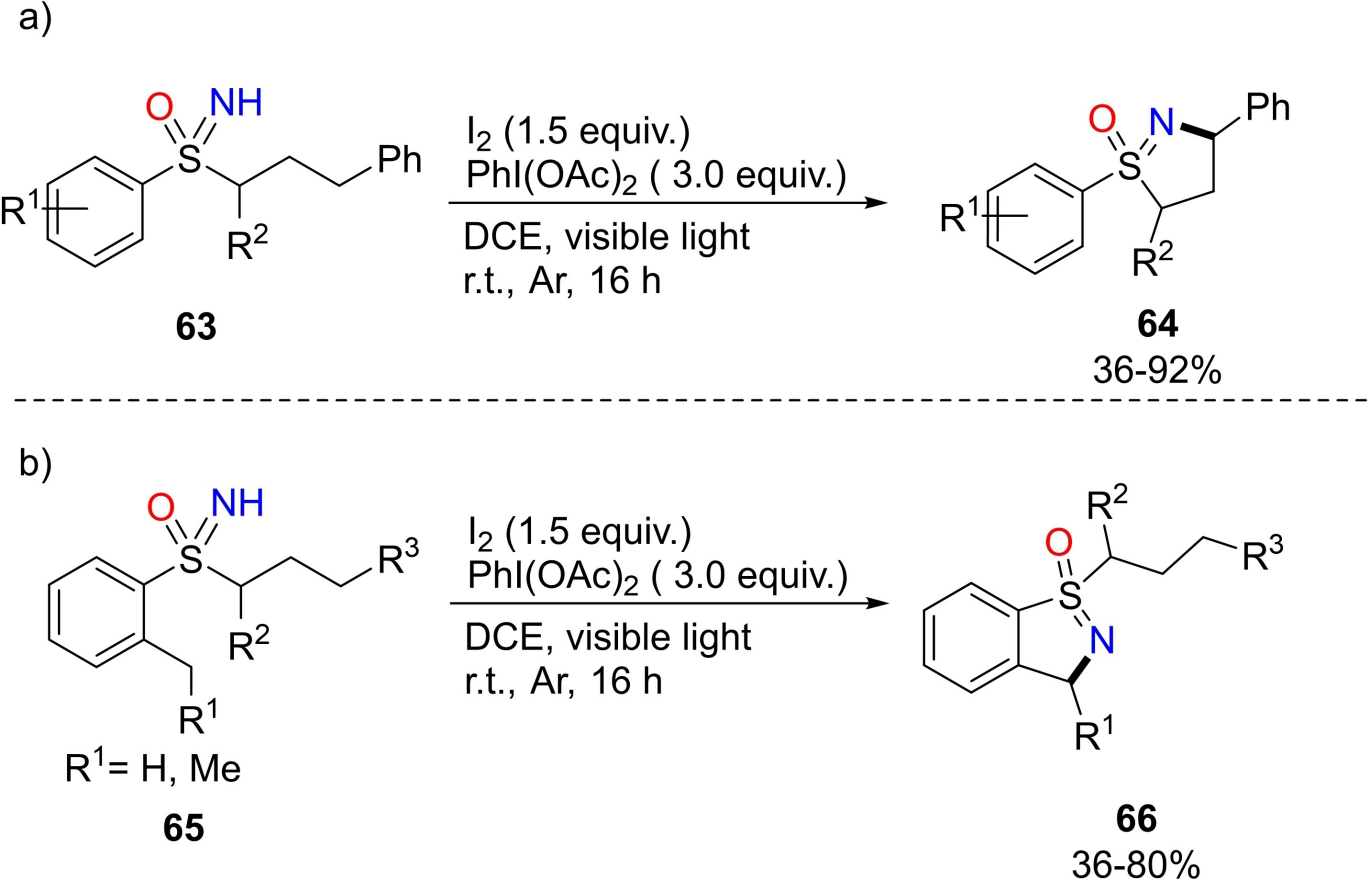
Synthesis of dihydroisothiazoles and benzo[*d*]isothiazoles‐1‐oxides.

Two years later, the same research group developed a strategy for the synthesis of dihydroisothiazole oxides **64** from S‐aryl‐S‐phenylpropyl‐NH‐sulfoximines **63** (Scheme 59, a).^[142]^ The transformation, a Hofmann‐Löffler‐Freytag type cyclization reaction, needs molecular iodine, diacetoxyiodobenzene and visible light irradiation. Similarly, benzo[*d*]isothiazoles‐1‐oxides **66** can be obtained upon the same reaction conditions from *ortho*‐alkyl substituted S‐arylsulfoximines **65** (Scheme 59, b). Moreover, when *ortho*‐alkyl substituted S‐aryl‐S‐phenylpropylsulfoximines were used, the reaction afforded a mixture of dihydroisothiazole oxides and benzo[*d*]isothiazoles‐1‐oxides.

**Scheme 60:**
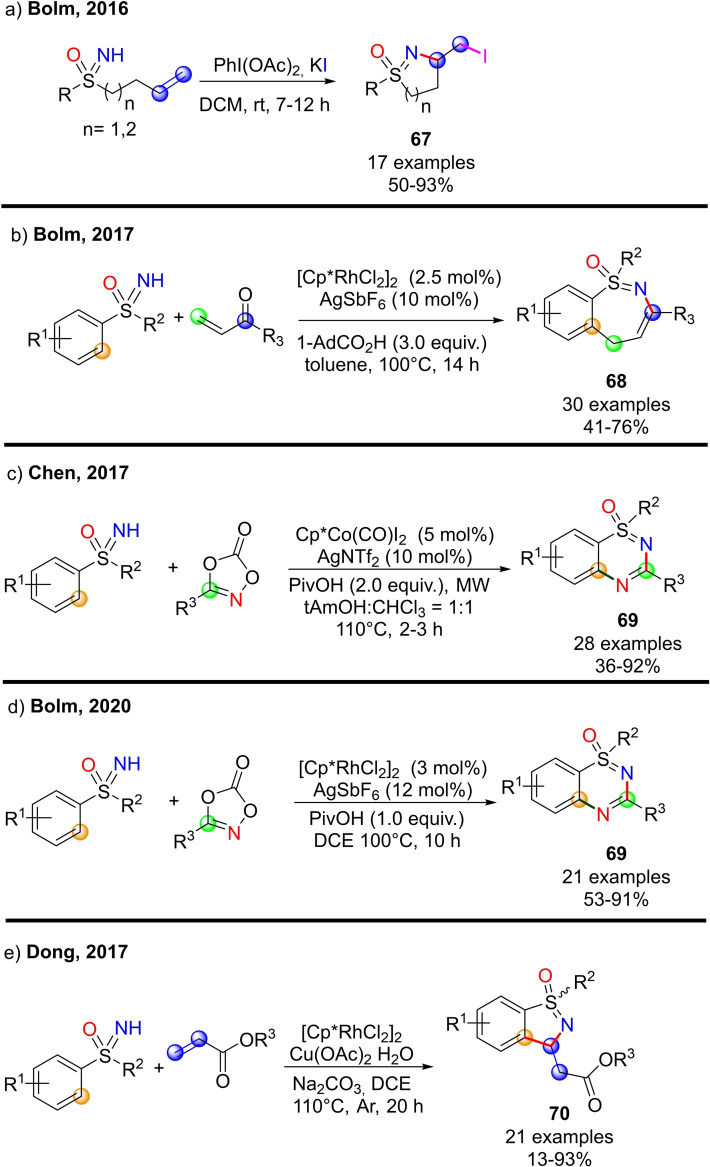
Examples of cyclization reactions involving NH‐sulfoximines.

In 2016, Bolm reported an efficient method for the halocyclization of NH‐sulfoximines towards the synthesis of S‐oxides of dihydro isothiazoles and tetrahydro‐1,2‐thiazines, in the presence of (diacetoxyiodo) benzene as the oxidant and potassium iodide as the halogen source.^[143]^ The reaction occurred with excellent regio‐ and stereoselectivity affording the corresponding five and six‐membered heterocycles **67** in good to excellent yields (Scheme 60, a). The interest toward benzothiazepines scaffold,^[144]^ inspired Bolm and co‐workers in developing a new method for the synthesis of 1,2‐benzothiazepine 1‐oxides **68** via a Rh‐catalyzed [4+3] annulations of NH‐sulfoximines with α,β‐unsaturated ketones.^[145]^ A wide range of functional groups were well tolerated, and the heterocyclic products could be obtained in high yields (Scheme 60, b). Moreover, thiadiazine 1‐oxides **69** could be efficiently prepared by the Cp*Co(III)‐catalyzed reaction of NH‐sulfoximines and 1,4,2‐dioxazol‐5‐ones as reported by Chen (Scheme 60, c).^[146]^ Bolm developed the synthesis of thiadiazine 1‐oxides from sulfoximines and 1,4,2‐dioxazol‐5‐ones using rhodium catalysis.^[147]^ The reaction proceeds in dichloroethane, affording the desired products **69** in good yields (scheme 60, d). In 2017, Dong and Li described the synthesis of benzoisothiazole **70** by tandem annulation of NH‐sulfoximines and olefins (Scheme 60, e).^[148]^ The reaction involves the ortho C−H activation, olefination, and subsequent intramolecular aza‐Michael cyclization. Good yields for the desired products were achieved by using [Cp*RhCl_2_]_2_ as the catalyst, Cu(OAc)_2_^.^H_2_O as the oxidant, Na_2_CO_3_ as the base, and conduction the reaction in DCE at 110 °C. Moreover, the presence of a variety of functional groups was tolerated.

**Scheme 61:**
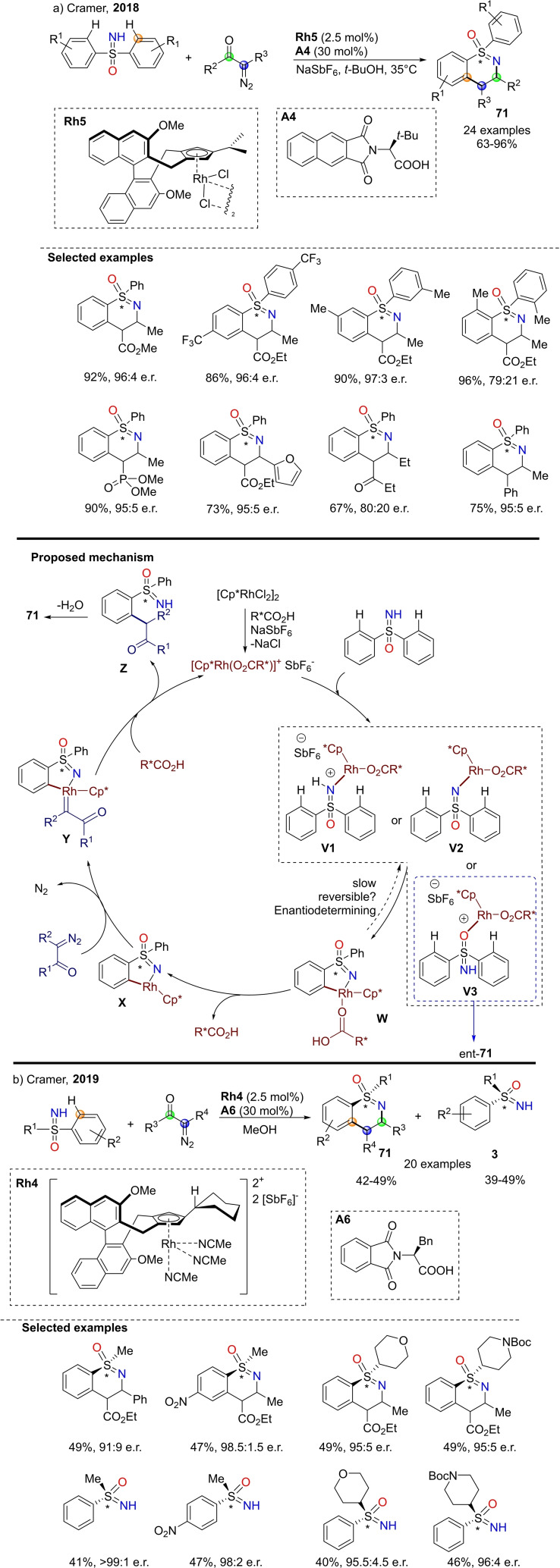
Synthesis of optically active 1,2‐benzothiazine from NH‐sulfoximines and diazoketones.

Recently, Cramer and coworkers disclosed the enantioselective preparation of S‐chiral 1,2‐benzothiazine via NH‐sulfoximines C−H functionalization with diazoketones catalyzed by optically active Rh(III) cyclopentadienyl‐based complexes (Scheme 61, a).^[149]^ The reported method proceeds efficiently with a broad range of diazoketones and affords the corresponding products **71** with high enantioselectivity using diverse substituted diarylsulfoximines. Moreover, the selectivity of the reaction was found to be boosted by the presence of a chiral optically active carboxylic acid. The transformation is thought to begin with the coordination of NH‐sulfoximine to the Rh(III) center giving intermediates **V1** or **V2**, that evolves towards the enantio‐determining *ortho*‐C−H activation through a concerted metalation‐deprotonation pathway affording intermediate **W** (Scheme 61, a). Subsequently, the coordination of the diazo compound promotes the formation of carbenoid species **Y**, that undergoes insertion and deprotonation leading to ketone **Z**, which affords sulfoximine **71** after condensation with loss of water. Reasonably, the coordination of sulfoximines from the oxygen atom would lead to a different complex (**V3**), that may evolve towards the product with inverted enantio‐selection (ent‐**71**). A year later, the same group developed a successful kinetic resolution of aryl alkyl NH‐sulfoximines via the C−H functionalization upon similar conditions (Scheme 61, b).^[150]^ In this case, a single enantiomer of the starting sulfoximine is efficiently transformed into the corresponding 1,2‐benzothiazine **71**, while the other remains unreacted, and can be isolated in excellent optical purity.

**Scheme 62:**
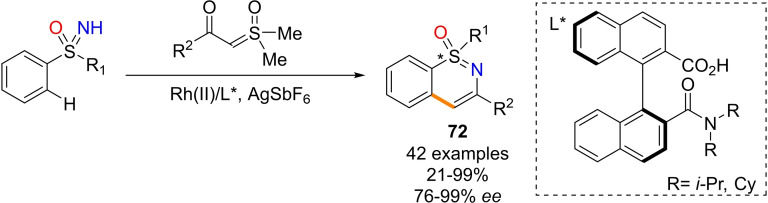
Synthesis of optically active 1,3‐disubtituted‐1λ^4^‐benzo[*e*][1,2]thiazines 1‐oxide.

Shi and co‐workers reported the preparation of chiral 1,3‐disubstituted‐1λ^4^‐benzo[*e*][1,2]thiazines 1‐oxides **72** with excellent enantioselectivity from NH‐sulfoximines and α‐carbonyl sulfoxonium ylides upon Ru(II) catalysis (Scheme 62).^[151]^ The reaction proceeds through a C−H activation/annulation process and uses chiral binaphthyl monocarboxylic acids as the chiral ligands. The products were thereby obtained in high yields and enantioselectivity by desymmetrization or kinetic resolution.

**Scheme 63:**
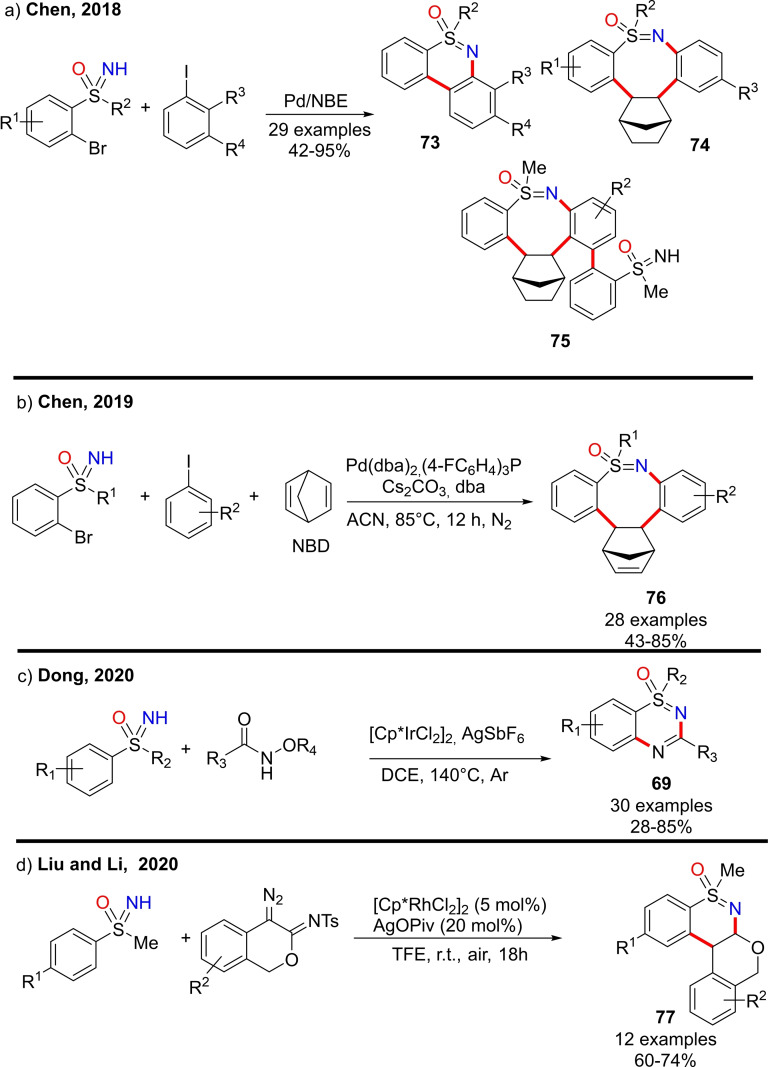
Other cyclization reactions involving NH‐sulfoximines.

In 2018, Chen and co‐workers developed a facile synthesis of polycyclic sulfoximine derivatives by one‐pot and one‐step annulation reaction, employing NH‐sulfoximines and aryl iodide as substrates, and Pd(OAc)_2_/norbornene (NBE) as catalysts to afford divergent tricyclic dibenzothiazines **73** or eight‐membered fused heterocyclic sulfoximines **74** and **75** (Scheme 63, a).^[152]^ Operational convenience, excellent selectivity, and good functional groups tolerance characterize this strategy. A similar approach for the formation of fused medium‐sized sulfoximine polyheterocycles **76** has been also reported.^[153]^ The method consists of a multicomponent reactions of NH‐sulfoximines with aryl iodides, and norbornadiene (NBA), in the presence of Pd(dba)_2_ as the catalyst, (4‐F‐C_6_H_4_)_3_P as the phosphine ligand, (Scheme 63, b). Very recently, a novel one‐pot strategy for the synthesis of various functionalized thiadiazine‐1‐oxides via C−H activation/cyclization between NH‐sulfoximines and N‐alkoxyamides was developed by Dong.^[154]^ High yields of the corresponding products **69** are therefore accessible by using [Cp*IrCl_2_]_2_ and AgSbF_6_ as catalysts, in DCE at 140 °C (Scheme 63, c). In addition, fused isochromeno‐1,2‐benzothiazines **77** are accessible from sulfoximines, as reported by Liu, Li and coworkers (Scheme 63, d).^[155]^ The reaction involved the use of S‐phenyl sulfoximines and 4‐diazoisochroman‐3‐imine as the substrates, and needed a rhodium (III) catalysis, affording the desired products in moderate to good yield.

**Scheme 64:**
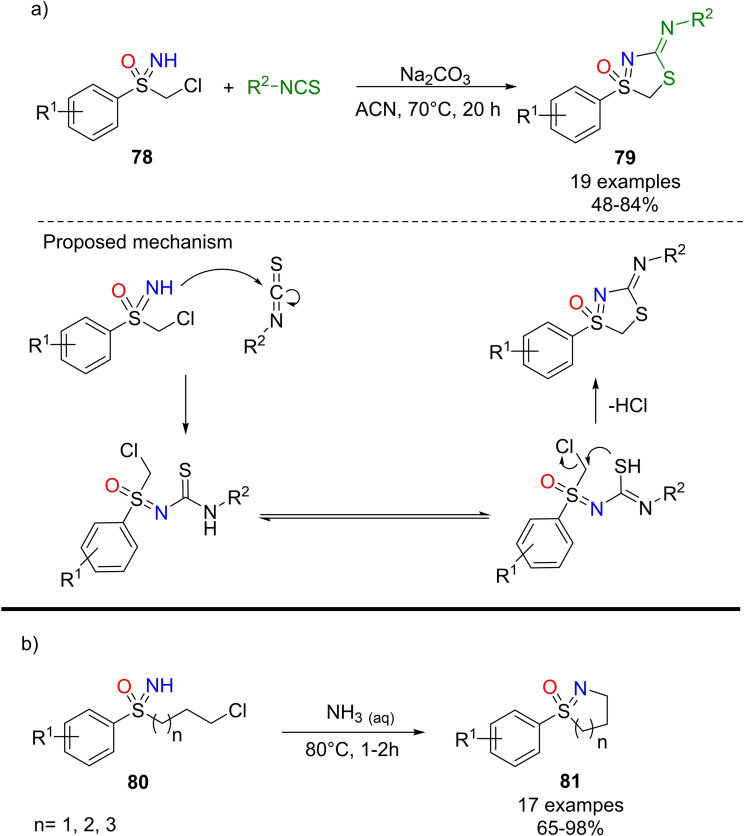
Synthesis of endocyclic sulfoximines.

Novel five‐membered endocyclic sulfoximines can be prepared by the reaction of S‐chloromethyl NH‐sulfoximines **78** and aryl isocyanates, as reported by Li and Ge.^[156]^ The reaction scope was investigated under optimal conditions (with N_2_CO_3_ as base in acetonitrile at 70 °C for 20 h), affording the desired products **79** in good to high yields (Scheme 64, a). The proposed mechanism involves the nucleophilic attach of sulfoximine to isothiocyanate, followed by the intramolecular ring closing reaction from the tautomeric thiol derivative, with loss of HCl. In 2020, Lücking reported the synthesis of five‐, six‐, and seven‐membered cyclic sulfoximines **81** by reacting chloroalkylsulfoximines **80** with an aqueous solution of ammonia at 80 °C (Scheme 64, b).^[157]^

## 5 Conclusions

Sulfoximines, the aza‐analogs of sulfones, have emerged as promising lead compounds in medicinal chemistry, and useful building blocks for organic synthesis and catalysis. We summarized the most recent advances in the field focusing on modern tactics to access NH‐sulfoximines encompassing the most recent methods for their transformation. Selective N−H functionalizations of sulfoximines including metal catalyzed and metal‐free methods of N‐arylation, N‐acylation, N‐phosphorylation, N‐sulfenylation, N‐sulfonylation N‐halogenation, and other useful elaborations of the sulfoximine group, have been collected. The use of more sustainable technology as the flow technology, and the fine control of the stereochemistry at the sulfur center has been discussed. This review mostly considered progress and achievements from 2015 showcasing the importance, and the need of fundamental research in this field. Moreover, many challenges and opportunities are foreseen for the future, and we hope that reading this review will stimulate synthetic chemists to develop research projects including these fascinating aza‐analogues of sulfones.

## Conflict of interest

The authors declare no conflict of interest.

## Biographical Information

*Michael Andresini obtained his M.Sci. degree (summa cum laude) in Chemical Sciences from University of Bari in 2018. After a short experience at BCMaterials (Basque Country, Spain), in 2019 he returned to University of Bari where he joined the PhD program in Drug Sciences under the supervision of Prof. Renzo Luisi. His research activity is focused on the development of synthetic strategies for the preparation of sulfur‐based functional groups and heterocycles, organometallic mediated transformations, and the use of microfluidic technology*.

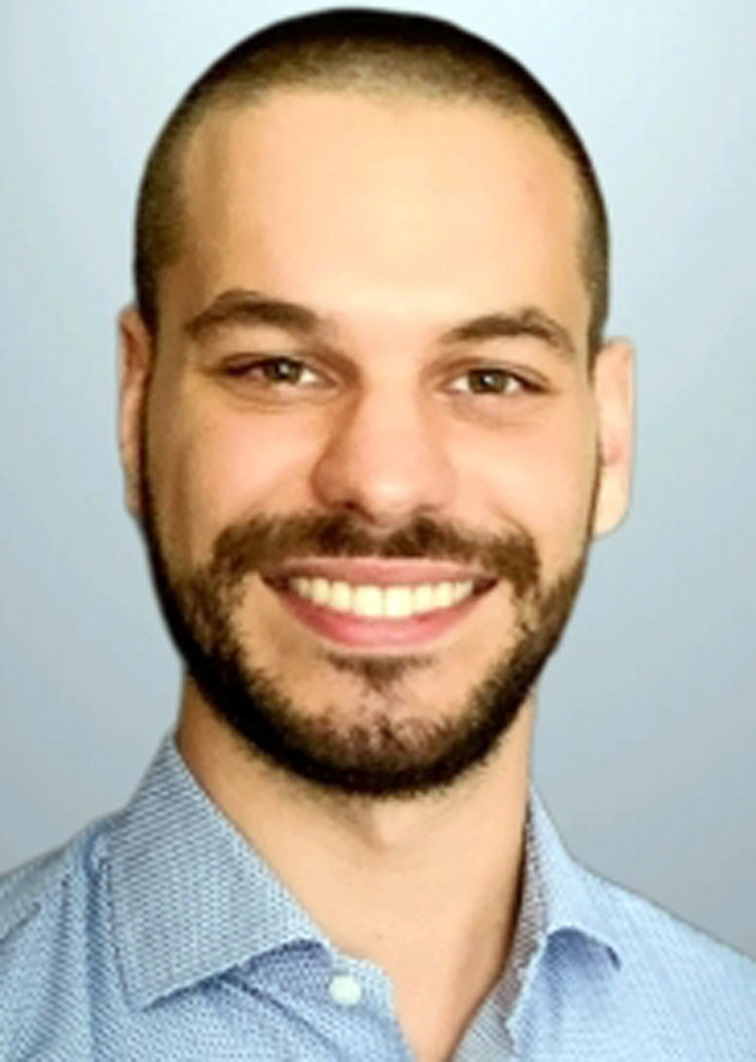

## Biographical Information

*Arianna Tota obtained the M.Sci. (summa cum laude) in Chemistry and Pharmaceutical Technology at the University of Bari (Italy) in 2015. In 2020, she obtained the Ph.D. in Chemical and Molecular Sciences under the supervision of Prof. Renzo Luisi. Her research activity is focused on the electrophilic nitrogen transfer to sulfur and the chemistry of nitrogen‐bearing compounds. In 2019, she has been a visiting scholar at the Department of Synthetic Chemistry and Biological Chemistry, Kyoto University (Japan), working in the group of Prof. Aiichiro Nagaki. During this time, she was involved in the field of flow microreactor technology applied to organometallic chemistry*.

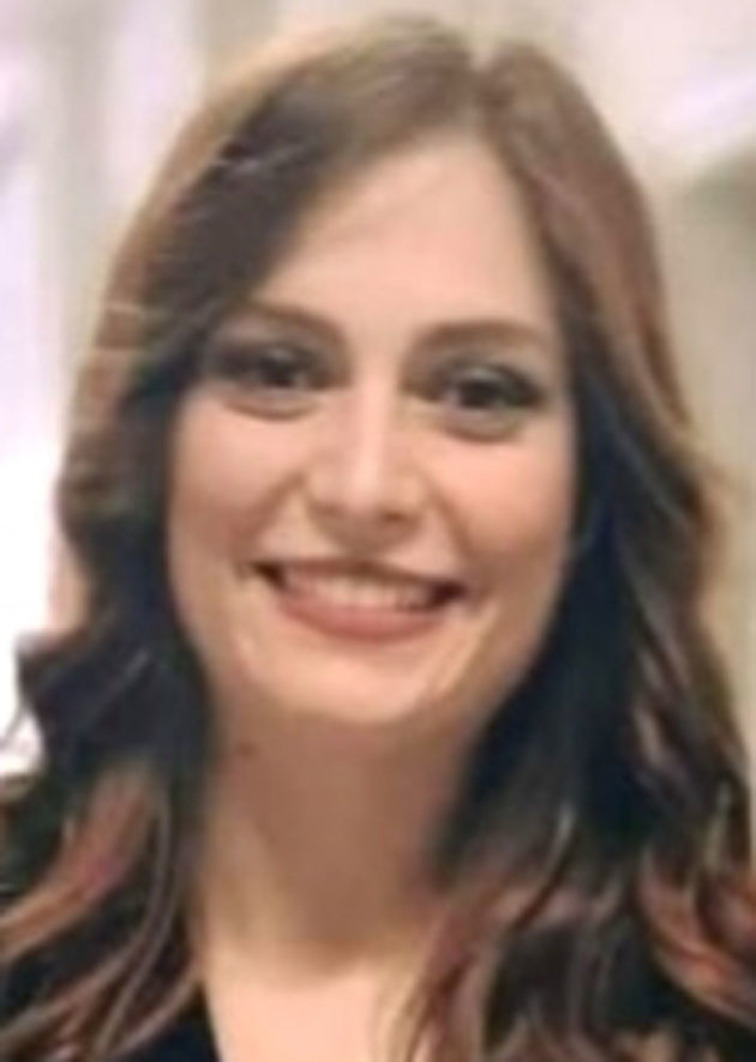

## Biographical Information

*Leonardo Degennaro obtained the master degree in Chemistry and Pharmaceutical Technology in 1999 and the PhD in Applied Chemical and Enzymatic Synthesis in 2003. In 2002 he was “visiting scholar” at the University of Groningen under the supervision of Prof. B. L. Feringa. In 2006 he was appointed assistant professor in Organic Chemistry at the Department of Pharmacy of University of Bari. In 2011 he has been “visiting assistant professor” at the University of Kyoto working in the group of Prof. J.‐i. Yoshida. The research activity is aimed at developing new stereocontrolled synthesis by using small heterocycles and organometallic species, and microreactor technology*

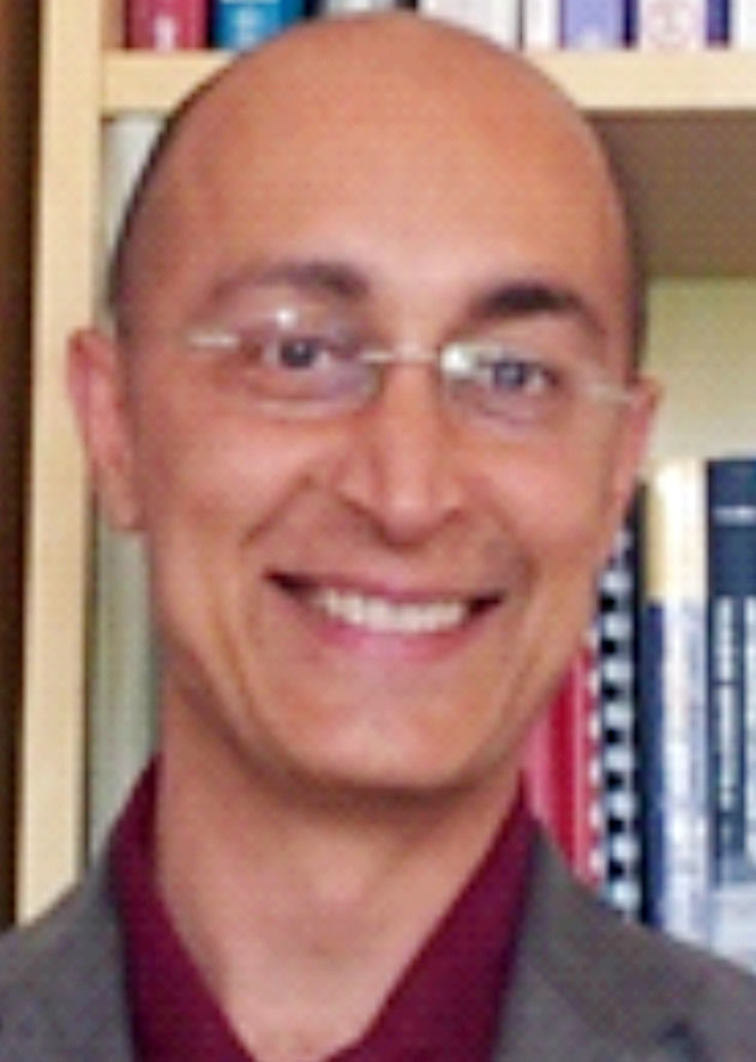

## Biographical Information

*Dr James Bull is a University Research Fellow at Imperial College London. His research focuses on the development of synthetic and catalytic methods to access medicinally relevant structural motifs and heterocycles. He obtained his MSci degree from the University of Cambridge, then spent a year at GlaxoSmithKline. He returned to University of Cambridge for his PhD with Professor Steven Ley. In 2007 he joined Université de Montréal as a postdoc with Professor André Charette. He started a Ramsay Memorial Fellowship at Imperial College in 2009, an EPSRC Career Acceleration Fellowship in 2011, and in 2016 was awarded a Royal Society University Research Fellowship*.

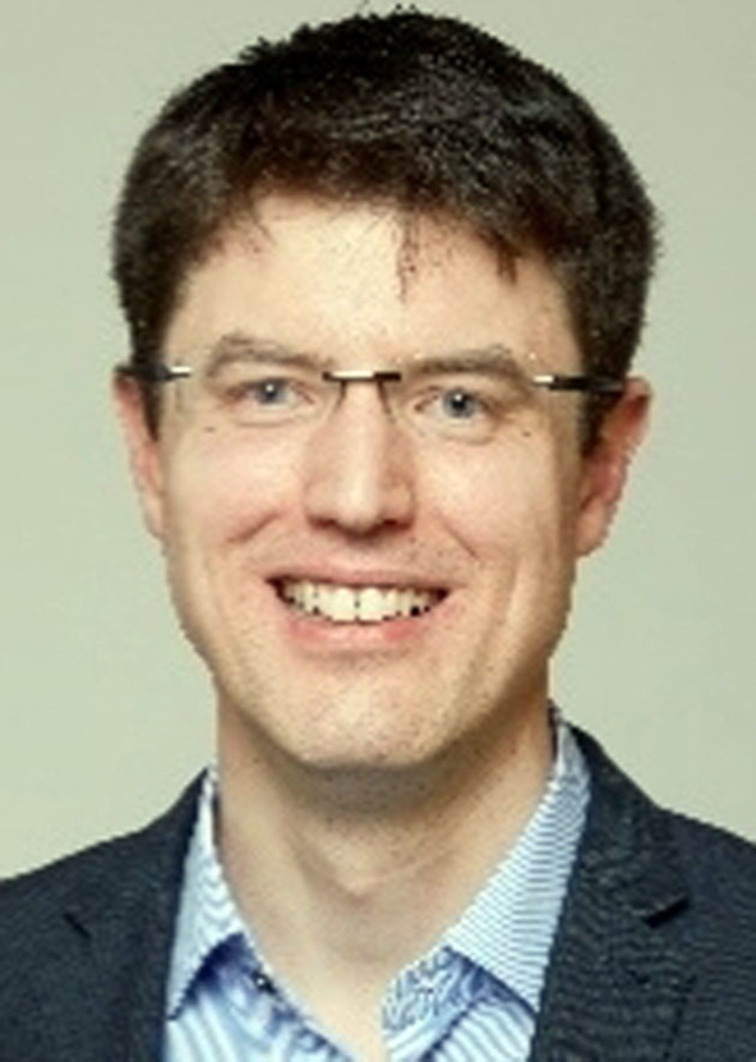

## Biographical Information

*Renzo Luisi is full professor of Organic Chemistry at the University of Bari (Italy). The research activity focuses on the chemistry of hetero‐substituted organolithiums, the development of new synthetic methodologies, and the use of flow technology. He obtained the PhD in 2000 under the guidance of Professor Saverio Florio. He has been visiting student at the Roger Adams Lab at Urbana Champaign in the group of Prof. Peter Beak, and visiting professor at the University of Manchester in the group of Jonathan Clayden. He is RSC fellow and recipient of the 2014 CINMPIS award Innovation in Organic Synthesis*.

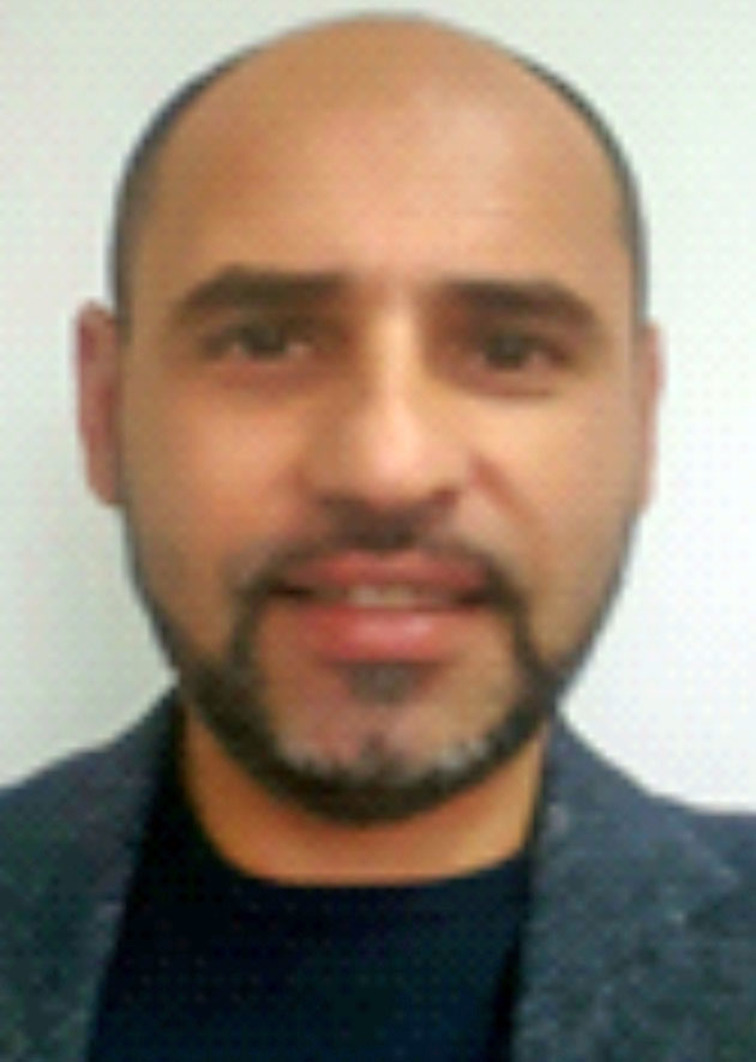

## Supporting information

As a service to our authors and readers, this journal provides supporting information supplied by the authors. Such materials are peer reviewed and may be re‐organized for online delivery, but are not copy‐edited or typeset. Technical support issues arising from supporting information (other than missing files) should be addressed to the authors.

Supporting Information

## References

[1a] U. Lücking , Angew. Chem. 2013, 125, 9570–9580;

[1b] U. Lücking , Org. Chem. Front. 2019, 6, 1319–1324.

[2] K. E. Arndt , D. C. Bland , N. M. Irvine , S. L. Powers , T. P. Martin , J. R. McConnell , D. E. Podhorez , J. M. Renga , R. Ross , G. A. Roth , B. D. Scherzer , T. W. Toyzan , Org. Process Res. Dev. 2015, 19, 454–462.

[3] J. M. Manning , S. Moore , W. B. Rowe , A. Meister , Biochemistry 1969, 8, 2681–2685. 5799144 10.1021/bi00834a066

[4] P. G. Richman , M. Orlowski , A. Meister , J. Biol. Chem. 1973, 248, 6684–6690. 4147652

[5] H. R. Bentley , E. E. McDermott , J. Pace , J. K. Whitehead , T. Moran , Nature 1949, 163, 675–676.18120748

[6a] P. Mäder , L. Kattner , J. Med. Chem. 2020, 63, 14243; 32870008 10.1021/acs.jmedchem.0c00960

[6b] Y. Han , K. Xing , J. Zhang , T. Tong , Y. Shi , H. Cao , H. Yu , Y. Zhang , D. Liu , L. Zhao , Eur. J. Med. Chem. 2021, 209, 112885; 33227576 10.1016/j.ejmech.2020.112885

[6c] C. Gnamm , A. Jeanguenat , A. C. Dutton , C. Grimm , D. P. Kloer , A. J. Crossthwaite , Bioorg. Med. Chem. Lett. 2012, 22, 3800. 22552196 10.1016/j.bmcl.2012.03.106

[7a] H. H. Bailey , Chem.-Biol. Interact. 1998, 111–112, 239–254; 10.1016/s0009-2797(97)00164-6 9679558

[7b] B. Hernández-Breijo , J. Monserrat , S. Ramírez-Rubio , E. P. Cuevas , D. Vara , I. Díaz-Laviada , M. D. Fernández-Moreno , I. D. Román , J. P. Gisbert , L. G. Guijarro , World J. Gastroenterol. 2011, 17, 3899–3911. 22025878 10.3748/wjg.v17.i34.3899 PMC3198019

[8a] U. Lücking , R. Jautelat , M. Krüger , T. Brumby , P. Lienau , M. Schäfer , H. Briem , J. Schulze , A. Hillisch , A. Reichel , A. M. Wengner , G. Siemeister , ChemMedChem 2013, 8, 1067–1085; 23671017 10.1002/cmdc.201300096

[8b] G. Siemeister , U. Luecking , A. M. Wengner , P. Lienau , W. Steinke , C. Schatz , D. Mumberg , K. Ziegelbauer , Mol. Cancer Ther. 2012, 11, 2265–2273. 22821149 10.1158/1535-7163.MCT-12-0286

[9a] A. Scholz , T. Oellerich , A. Hussain , S. Lindner , U. Luecking , A. O. Walter , P. Ellinghaus , R. Valencia , F. von Nussbaum , D. Mumberg , M. Brands , S. Ince , H. Serve , K. Ziegelbauer , Cancer Res. 2016, 76, 3022;

[9b] A. Scholz , U. Luecking , G. Siemeister , P. Lienau , U. Boemer , P. Ellinghaus , A. O. Walter , R. Valencia , S. Ince , F. von Nussbaum , D. Mumberg , M. Brands , K. Ziegelbauer , Cancer Res. 2015, 75, DDT02–02;

[9c] U. Luecking , A. Scholz , P. Lienau , G. Siemeister , D. Kosemund , R. Bohlmann , K. Eis , M. Gnoth , I. Terebesi , K. Meyer , K. Prelle , R. Valencia , S. Ince , F. von Nussbaum , D. Mumberg , K. Ziegelbauer , B. Klebl , A. Choidas , P. Nussbaumer , M. Baumann , C. Schultz-Fademrecht , G. Ruehter , J. Eickhoff , M. Brands , Cancer Res. 2015, 75, 2828.

[10] K. M. Foote , A. Lau , J. W. M. Nissink , Future Med. Chem. 2015, 7, 873–891. 26061106 10.4155/fmc.15.33

[11] C. Gege , F. J. Bravo , N. Uhlig , T. Hagmaier , R. Schmachtenberg , J. Elis , A. Burger-Kentischer , D. Finkelmeier , K. Hamprecht , T. Grunwald , et al., Sci. Transl. Med. 2021, 13, 598. 10.1126/scitranslmed.abf8668 34135112

[12] W. L. Mock , J.-T. Tsay , J. Am. Chem. Soc. 1989, 111, 4467–4472.

[13] S. Oae , K. Harada , K. Tsujihara , N. Furukawa , Int. J. Sulfur Chem. 1972, 2, 49–61.

[14] J. A. Sirvent , U. Lücking , ChemMedChem. 2017, 12, 7, 487–501. 10.1002/cmdc.201700044 PMC5485063 28221724

[15a] C. R. Johnson , Acc. Chem. Res. 1973, 6, 341–347;

[15b] D. Craig , F. Grellepois , A. J. P. White , J. Org. Chem. 2005, 70, 6827–6832; 16095302 10.1021/jo050747d

[15c] H.-J. Gais , G. S. Babu , M. Günter , P. Das , Eur. J. Org. Chem. 2004, 1464–1473;

[15d] M. Harmata , X. Hong , J. Am. Chem. Soc. 2003, 125, 5754–5756; 12733915 10.1021/ja034744z

[15e] M. Harmata , X. Hong , C. L. Barnes , Tetrahedron Lett. 2003, 44, 7261–7264;

[15f] M. Harmata , N. Pavri , Angew. Chem. Int. Ed. 1999, 38, 2419–2421; 10.1002/(sici)1521-3773(19990816)38:16<2419::aid-anie2419>3.0.co;2-i 10458808

[15g] S. Koep , H.-J. Gais , G. Raabe , J. Am. Chem. Soc. 2003, 125, 13243–13251; 14570500 10.1021/ja030324y

[15h] X. Shen , W. Miao , C. Ni , J. Hu , Angew. Chem. Int. Ed. 2014, 53, 775–779; 10.1002/anie.201308484 24307659

[15i] X. Shen , Q. Liu , W. Zhang , J. Hu , Eur. J. Org. Chem. 2016, 906–909.

[16a] C. Bolm , O. Simic , J. Am. Chem. Soc. 2001, 123, 3830–3831; 11457120 10.1021/ja004261k

[16b] M. Harmata , S. K. Ghosh , Org. Lett. 2001, 3, 3321–3323; 11594824 10.1021/ol016546n

[16c] C. Bolm , M. Martin , O. Simic , M. Verrucci , Org. Lett. 2003, 5, 427–429; 12583735 10.1021/ol027273e

[16d] C. Bolm , M. Felder , J. Müller , Synlett 1992, 5, 439–441;

[16e] C. Bolm , M. Verrucci , O. Simic , P. G. Cozzi , G. Raabe , H. Okamura , Chem. Commun. 2003, 22, 2826–2827; 10.1039/b309556h 14651124

[16f] M. Langner , C. Bolm , Angew. Chem. Int. Ed. 2004, 43, 5984–5987; 10.1002/anie.200460953 15547912

[16g] M. Langner , P. Remy , C. Bolm , Chem. Eur. J. 2005, 11, 6254–6265; 16075444 10.1002/chem.200500497

[16h] M. T. Reetz , O. G. Bondarev , H.-J. Gais , C. Bolm , Tetrahedron Lett. 2005, 46, 5643–5646.

[17] S. Wiezorek , P. Lamers , C. Bolm , Chem. Soc. Rev. 2019, 48, 5408–5423. 31535112 10.1039/c9cs00483a

[18a] C. R. Johnson , Aldrichimica Acta 1985, 18, 3–10;

[18b] M. Reggelin , C. Zur , Synthesis 2000, 1, 1–64;

[18c] H.-J. Gais , Heteroat. Chem. 2007, 18, 472–481;

[18d] M. Harmata , Chemtracts 2003, 16, 660–666;

[18e] H. Okamura , C. Bolm , Chem. Lett. 2004, 33, 482–487;

[18f] J. A. Bull , L. Degennaro , R. Luisi , Synlett 2017, 28, 2525–2538;

[18g] P. Ghosh , B. Ganguly and S. Das , Asian J. Org. Chem. 2020, 9, 2035–2082.

[19] Bizet , C. M. M. Hendriks and C. Bolm , Chem. Soc. Rev. 2015, 44, 3378–3390. 25941981 10.1039/c5cs00208g

[20] H. Okamura , C. Bolm , Org. Lett. 2004, 6, 1305–1307. 15070323 10.1021/ol049715n

[21a] C. R. Johnson , C. W. Schroeck , J. Am. Chem. Soc. 1973, 95, 7418–7423;

[21b] Y. Tamura , J. Minamikawa , K. Sumoto , S. Fujii , M. Ikeda , J. Org. Chem. 1973, 38, 1239–1241; 4691167 10.1021/jo00946a045

[21c] C. R. Johnson , R. A. Kirchhoff , H. G. Corkins , J. Org. Chem. 1974, 39, 2458–2459.

[22] T. Siu , A. K. Yudin , Org. Lett. 2002, 4, 1839–1842. 12027627 10.1021/ol0257530

[23] J. L. Jat , M. P. Paudyal , H. Gao , Q.-L. Xu , M. Yousufuddin , D. Devarajan , D. H. Ess , L. Kürtis , J. R. Falck , Science 2014, 343, 61–65. 24385626 10.1126/science.1245727 PMC4175444

[24] J. Miao , N. G. J. Richards , H. Ge , Chem. Commun. 2014, 50, 9687–9689. 10.1039/c4cc04349a 25016917

[25] J. Wang , J. Zhang , K. Miao , H. Yun , H. C. Shen , W. Zhao , C. Liang , Tetrahedron Lett. 2017, 58, 333–337.

[26a] P. E. Eaton , G. R. Carlson , J. T. Lee , J. Org. Chem. 1973, 38, 4071;

[26b] S. C. Virgil, in *Encyclopedia of Reagents for Organic Synthesis*, L. A. Paquette, Ed. John Wiley & Sons, Chichester **2001**, p. 4129–4132.

[27a] A. Mertens , K. Lammertsma , M. Arvanaghi , G. A. Olah , J. Am. Chem. Soc. 1983, 105, 5657–5660;

[27b] G. A. Olah , T. D. Ernst , J. Org. Chem. 1989, 54, 1203–1204.

[28] M. Zenzola , R. Doran , L. Degennaro , R. Luisi , J. A. Bull , Angew. Chem. Int. Ed. 2016, 51, 7203–7207. 10.1002/anie.201602320 PMC5074267 27126053

[29a] K. D. Collins , F. Glorius , Nat. Chem. 2013, 5, 597–601; 23787750 10.1038/nchem.1669

[29b] K. D. Collins , A. Rühling , F. Glorius , Nat. Protoc. 2014, 9, 1348–1353; 24833173 10.1038/nprot.2014.076

[29c] K. D. Collins , F. Glorius , Acc. Chem. Res. 2015, 48, 619–627. 25699585 10.1021/ar500434f

[30a] C. G. Espino , J. Du Bois , Angew. Chem. Int. Ed. 2001, 40, 598–600; 10.1002/1521-3773(20010202)40:3<598::AID-ANIE598>3.0.CO;2-9 29712035

[30b] C. Iacobucci , S. Reale , F. De Angelis , Angew. Chem. Int. Ed. 2016, 55, 2980–2993; 10.1002/anie.201507088 26799781

[31] A. S. Ivanov , I. A. Popov , A. I. Boldyrev , V. V. Zhdankin , Angew. Chem. Int. Ed. 2014, 53, 9617–9621; 10.1002/anie.201405142 25045143

[32] M. Ochiai , T. Kaneaki , N. Tada , K. Miyamoto , H. Chuman , M. Shiro , S. Hayashi , W. Nakanishi , J. Am. Chem. Soc. 2007, 129, 12938–12939. 17915875 10.1021/ja075811i

[33] M. A. Graham, H. Askey, A. D. Campbell, L. Chan, K. G. Cooper, Z. Cui, A. Dalgleish, D. Dave, G. Ensor, M. R. Galan Espinosa, et al., *Org. Process Res. Dev*. **2021**, *25*, 43–56.

[34] K. M. Foote , J. W. M. Nissink , T. McGuire , P. Turner , S. Guichard , J. W. T. Yates , A. Lau , K. Blades , D. Heathcote , R. Odedra , et al., J. Med. Chem. 2018, 61, 9889–9907. 30346772 10.1021/acs.jmedchem.8b01187

[35] H. C. Englert , U. Gerlach , H. Goegelein , J. Hartung , H. Heitsch , D. Mania , S. Scheidler , J. Med. Chem. 2001, 44, 1085–1098. 11297455 10.1021/jm000985v

[36] B. Gutmann , P. Elsner , A. O'Kearney-McMullan , W. Goundry , D. M. Roberge , C. O. Kappe , Org. Process Res. Dev. 2015, 19, 1062–1067.

[37a] K. M. Foote, J. W. M. Nissink, P. Turner, AstraZeneca AB, Swed., AstraZeneca UK Limited, *Morpholino pyrimidines and their use in therapy* WO2011154737 A1, **2011**, CAN156 : 74464;

[37b] I. Collins, M. D. Garrett, *Curr. Opin. Pharmacol*. **2005**, *5*, 366–373; 10.1016/j.coph.2005.04.009 15964238

[37c] W. G. Kaelin, *Nat. Rev. Cancer* **2005**, *5*, 689–698. 10.1038/nrc1691 16110319

[38] W. R. F. Goundry , K. Dai , M. Gonzalez , D. Legg , A. O'Kearney-McMullan , J. Morrison , A. Stark , P. Siedlecki , P. Tomlin , J. Yang , Org. Process Res. Dev. 2019, 23, 1333–1342.

[39] A. Tota , M. Zenzola , S. J. Chawner , S. St John-Campbell , C. Carlucci , G. Romanazzi , L. Degennaro , J. A. Bull , R. Luisi , Chem. Commun. 2017, 53, 348–351. 10.1039/c6cc08891k 27929152

[40] J.-F. Lohier , T. Glachet , H. Marzag , A. C. Gaumont , V. Reboul , Chem. Commun. 2017, 53, 2064–2067. 10.1039/c6cc09940h 28133647

[41] For related studies on sulfonimidamides, also see: E. L. Briggs , A. Tota , M. Colella , L. Degennaro , R. Luisi , J. A. Bull , Angew. Chem. Int. Ed. 2019, 58, 14303–14310; 10.1002/anie.201906001 31390133

[42] Y. Xie , B. Zhou , S. Zhou , S. Zhou , W. Wei , J. Liu , Y. Zhan , D. Cheng , M. Chen , Y. Li , B. Wang , X.-S. Xue , Z. Li , ChemistrySelect 2017, 2, 1620–1624.

[43] G. Zhang , H. Tan , W. Chen , H. C. Shen , Y. Lu , C. Zheng , H. Xu , ChemSusChem 2020, 13, 922–928. 31950602 10.1002/cssc.201903430

[44] C. M. Gabriel , N. R. Lee , F. Bigorne , P. Klumphu , M. Parmentier , F. Gallou , B. H. Lipshutz , Org. Lett. 2017, 19, 194–197. 27997200 10.1021/acs.orglett.6b03468

[45] A. C. Barnes , P. W. Hairsine , S. S. Matharu , P. J. Ramm , J. B. Taylor , J. Med. Chem. 1979, 22, 418–424. 430479 10.1021/jm00190a012

[46] T. Glachet , X. Franck , V. Reboul , Synthesis 2019, 51, 4, 971–975.

[47a] U. Lücking, R. Bohlmann, A. Scholz, G. Siemeister, M. J. Gnoth, U. Bömer, D. Kosemund, P. Lienau, G. Rüther, C. Schulz-Fademrecht, PCT Int. Appl WO 2012160034, **2012**;

[47b] J. A. Sirvent , D. Bierer , R. Webster , U. Lücking , Synthesis 2017, 49, 1024–1036.

[48] A. Tota , C. Carlucci , L. Pisano , G. Cutolo , G. J. Clarkson , G. Romanazzi , L. Degennaro , J. A. Bull , P. Rollin and R. Luisi , Org. Biomol. Chem. 2020, 18, 3893–3897. 32392272 10.1039/d0ob00647e

[49] R. M. Bär , L. Langer , M. Nieger , S. Bräse , Adv. Synth. Catal. 2020, 362, 1356–1361.

[50] M. L. G. Borst , C. M. J. Ouairy , S. C. Fokkema , A. Cecchi , J. M. C. A. Kerckhoffs , V. L. de Boer , P. J. van den Boogaard , R. F. Bus , R. Ebens , R. van der Hulst , J. Knol , R. Libbers , Z. M. Lion , B. W. Settels , E. de Wever , K. A. Attia , P.-J. Sinnema , J. M. de Gooijer , K. Harkema , M. Hazewinkel , S. Snijder , K. Pouwer , ACS Comb. Sci. 2018, 20, 335–343. 29714998 10.1021/acscombsci.7b00150

[51] U. Lücking , G. Siemeister , M. Krüger , R. Jautelat , Sulfoximine-substituted pyrimidines as CDK-and/or VEGF inhibitors, their production and use as pharmaceutical agents 2008, U. S. Patent No. 7,338,958.

[52a] W. Zhang , F. Wang , J. Hu , Org. Lett. 2009, 11, 2109–2112; 19368376 10.1021/ol900567c

[52b] W. Zhang , W. Huang , J. Hu , Angew. Chem. Int. Ed. 2009, 48, 9858–9861; 10.1002/anie.200905077 19937885

[52c] W. Zhang , J. Hu , Adv. Synth. Catal. 2010, 352, 2799–2804.

[53a] Y. Arai , R. Tomita , G. Ando , T. Koike , M. Akita , Chem. Eur. J. 2016, 22, 1262–1265; 26639021 10.1002/chem.201504838 PMC4737308

[53b] N. Noto , T. Koike , M. Akita , J. Org. Chem. 2016, 81, 7064–7071; 27304729 10.1021/acs.joc.6b00953

[53c] M. Daniel , G. Dagousset , P. Diter , P.-A. Klein , B. Tuccio , A.-M. Goncalves , G. Masson , E. Magnier , Angew. Chem. Int. Ed. 2017, 56, 3997–4001; 10.1002/anie.201700290 28252241

[54] T.-N. Le , P. Diter , B. Pegot , C. Bournaud , M. Toffano , R. Guillot , G. Vo-Thanh , E. Magnier , Org. Lett. 2016, 18, 5102–5105. 27682457 10.1021/acs.orglett.6b02548

[55] P. Kirsch , M. Lenges , D. Kuehne , K.-P. Wanczek , Eur. J. Org. Chem. 2005, 797–802.

[56] S. Chaabouni , J.-F. Lohier , A.-L. Barthelemy , T. Glachet , E. Anselmi , G. Dagousset , P. Diter , B. Pégot , E. Magnier , V. Reboul , Chem. Eur. J. 2018, 24, 17006–17010. 30300956 10.1002/chem.201805055

[57] G. B. Craven , E. L. Briggs , C. M. Zammit , A. McDermott , S. Greed , D. P. Affron , C. Leinfellner , H. R. Cudmore , R. R. Tweedy , R. Luisi , et al., J. Org. Chem. 2021, 86, 7403–7424. 34003635 10.1021/acs.joc.1c00373

[58] N. Camaioni , G. Ridolfi , V. Fattori , L. Favaretto , G. Barbarella , Appl. Phys. Lett. 2004, 84, 1901–1903.

[59a] I. Viola , I. E. Palama , A. M. L. Coluccia , M. Biasiucci , B. Dozza , E. Lucarelli , F. Di Maria , G. Barbarella , G. Gigli , Integr. Biol. 2013, 5, 1057–1066; 10.1039/c3ib40064f 23806977

[59b] J. T. II Petroff , K. N. Skubic , C. K. Arnatt , R. D. McCulla , J. Org. Chem. 2018, 83, 14063–14068. 30339008 10.1021/acs.joc.8b01931

[60a] K. Uno , H. Niikura , M. Morimoto , Y. Ishibashi , H. Miyasaka , M. Irie , J. Am. Chem. Soc. 2011, 133, 13558–13564; 21819048 10.1021/ja204583e

[60b] O. Nevskyi , D. Sysoiev , A. Oppermann , T. Huhn , D. Wöll , Angew. Chem. Int. Ed. 2016, 55, 12698–12702; 10.1002/anie.201606791 27619176

[61] B. Verbelen , E. Siemes , A. Ehnbom , C. Räuber , K. Rissanen , D. Wöll , C. Bolm , Org. Lett. 2019, 21, 4293–4297. 31120764 10.1021/acs.orglett.9b01475

[62] H. Yu , Z. Li , C. Bolm , Angew. Chem. 2018, 130, 330–333;30407705

[63] K. E. Arndt , D. C. Bland , N. M. Irvine , S. L. Powers , T. P. Martin , J. R. McConnell , D. E. Podhorez , J. M. Renga , R. Ross , G. A. Roth , B. D. Scherzer , T. W. Toyzan , Org. Process Res. Dev. 2015, 19, 454–462.

[64a] U. Lücking , Angew. Chem. 2013, 125, 9570–9580;

[64b] M. Frings , C. Bolm , A. Blum , C. Gnamm , Eur. J. Med. Chem. 2017, 126, 225–245. 27821325 10.1016/j.ejmech.2016.09.091

[65] T. Q. Davies , M. J. Tilby , J. Ren , N. A. Parker , D. Skolc , A. Hall , F. Duarte , M. C. Willis , J. Am. Chem. Soc. 2020, 142, 15445–15453. 32841007 10.1021/jacs.0c06986 PMC7498162

[66] P. M. Matos , W. Lewis , S. P. Argent , J. C. Moore , R. A. Stockman , Org. Lett. 2020, 22, 2776–2780. 32176512 10.1021/acs.orglett.0c00761

[67] For related reports see:

[67a] P. M. Matos , W. Lewis , J. C. Moore , R. A. Stockman , Org. Lett. 2018, 20, 3674–3677; 29878785 10.1021/acs.orglett.8b01473

[67b] P. M. Matos , R. A. Stockman , Org. Biomol. Chem. 2020, 18, 6429–6442. 32797133 10.1039/d0ob01191f

[68] V. Semak , C. Escolano , C. Arróniz , J. Bosch , M. Amat , Tetrahedron: Asymmetry 2010, 21, 2542–2549.

[69] Y. Aota , T. Kano , K. Maruoka , J. Am. Chem. Soc. 2019, 141, 19263–19268. 31739663 10.1021/jacs.9b11298

[70] Y. Aota , T. Kano , K. Maruoka , Angew. Chem. Int. Ed. 2019, 58, 17661–17665; 10.1002/anie.201911021 31568618

[71a] C. P. R. Hackenberger , G. Raabe , C. Bolm , Chem. Eur. J. 2004, 10, 2942–2952; 15214076 10.1002/chem.200306016

[71b] P. Stoss , G. Satzinger , Tetrahedron Lett. 1973, 14, 267–268;

[71c] S. J. Park , H. Buschmann , C. Bolm , Bioorg. Med. Chem. Lett. 2011, 21, 4888–4890. 21752640 10.1016/j.bmcl.2011.06.029

[72] S. Dong , M. Frings , H. Cheng , J. Wen , D. Zhang , G. Raabe , C. Bolm , J. Am. Chem. Soc. 2016, 138, 2166–2169. 26847138 10.1021/jacs.6b00143

[73] T.-N. Le , P. Diter , B. Pégot , C. Bournaud , M. Toffano , R. Guillot , G. Vo-Thanh , E. Magnier , Org. Lett. 2016, 18, 5102–5105. 27682457 10.1021/acs.orglett.6b02548

[74] V. Snieckus , Chem. Rev. 1990, 90, 879–933.

[75] A.-L. Barthelemy , A. Prieto , P. Diter , J. Hannedouche , M. Toffano , E. Anselmi , E. Magnier , Eur. J. Org. Chem. 2018, 27, 3764–3770.

[76] S. G. Pyne , Z. Dong , Tetrahedron Lett. 1999, 40, 6131–6134.

[77] M. Harmata , S. K. Ghosh , Org. Lett. 2001, 3, 3321–3323. 11594824 10.1021/ol016546n

[78] S. G. Pyne , Tetrahedron Lett. 1986, 27, 1691–1694.

[79] D. M. David , M. Bakavoli , S. G. Pyne , B. W. Skelton , A. H. White , Tetrahedron 1995, 51, 12393–12402.

[80] S. K. Tiwari , H.-J. Gais , A. Lindenmaier , G. S. Babu , G. Raabe , L. R. Reddy , F. Köhler , M. Günter , S. Koep , V. B. R. Iska , J. Am. Chem. Soc. 2006, 128, 7360–7373. 16734492 10.1021/ja061152i

[81] D. Craig , N. J. Geach , Synlett 1993, 7, 481–482.

[82] G. Sklute , C. Bolm , I. Marek , Org. Lett. 2007, 9, 1259–1621. 17348664 10.1021/ol070070b

[83] P. K. Chinthakindi , G. C. Nandi , T. Govender , H. G. Kruger , T. Naicker , P. I. Arvidsson , Synlett 2016, 27, 1423–1427.

[84] M. J. McGrath , C. Bolm , Beilstein J. Org. Chem. 2007, 3, 33. 17939868 10.1186/1860-5397-3-33 PMC2100055

[85] P. Sen Kumar , P. V. Bharatam , Tetrahedron 2005, 61, 5633–5639.

[86a] A. Mertens , K. Lammertsma , M. Arvanaghi , G. A. Olah , J. Am. Chem. Soc. 1983, 105, 5657–5660;

[86b] G. A. Olah , T. D. Ernst , J. Org. Chem. 1989, 54, 1203–1204.

[87] L. Degennaro , A. Tota , S. De Angelis , M. Andresini , C. Cardellicchio , M. A. Capozzi , G. Romanazzi , R. Luisi , Eur. J. Org. Chem. 2017, 2017, 6486–6490.

[88] W. Zheng , M. Tan , L. Yang , R. R. Kuchukulla , L. Zhou , Q. Zeng , Eur. J. Org. Chem. 2020, 11, 1764–1768.

[89] L. Yang , J. Feng , M. Qiao , Q. Zeng , Org. Chem. Front. 2018, 5, 24–28.

[90] Y. Peng , Y. Lin , R. Nie , Y. Zheng , Y. Liu , L. Guo , Y. Wu , Eur. J. Org. Chem. 2018, 844–850.

[91] Y. Lin , Y. Liu , Y. Zheng , R. Nie , L. Guo , Y. Wu , ACS Sustainable Chem. Eng. 2018, 6, 13644–13649.

[92] S. Gupta , S. Baranwal , P. Chaudhary , J. Kandasamy , Org. Chem. Front. 2019, 6, 2260–2265.

[93a] M. R. Yadav , R. K. Rit , A. K. Sahoo , Chem. Eur. J. 2012, 18, 5541–5545; 22461080 10.1002/chem.201200092

[93b] M. R. Yadav , R. K. Rit , A. K. Sahoo , Org. Lett. 2012, 14, 3724–3727; 22765229 10.1021/ol301579q

[93c] M. R. Yadav , R. K. Rit , A. K. Sahoo , Org. Lett. 2013, 15, 1638–1641; 23477353 10.1021/ol400411v

[93d] F. Misani , T. W. Fair , L. Reiner , J. Am. Chem. Soc. 1951, 73, 459–461.

[94a] C. Bolm , J. D. Kahmann , G. Moll , Tetrahedron Lett. 1997, 38, 1169–1172;

[94b] C. Bolm , G. Moll , J. D. Kahmann , Chem. Eur. J. 2001, 7, 1118–1128. 11303871 10.1002/1521-3765(20010302)7:5<1118::aid-chem1118>3.0.co;2-3

[95] M. Shankar , K. Ghosh , K. Mukherjee , R. K. Rit , A. K. Sahoo , Org. Lett. 2016, 18, 6416–6419. 27978646 10.1021/acs.orglett.6b03314

[96] K. Mukherjee , M. Shankar , K. Ghosh , A. K. Sahoo , Org. Lett. 2018, 20, 1914–1918. 29561160 10.1021/acs.orglett.8b00468

[97] M. Shankar , K. Ghosh , K. Mukherjee , R. K. Rit , A. K. Sahoo , Org. Lett. 2018, 20, 5144–5148. 30095912 10.1021/acs.orglett.8b02068

[98] M. Shankar , R. K. Rit , S. Sau , K. Mukherjee , V. Gandon , A. K. Sahoo , Chem. Sci. 2020, 11, 10770–10777.

[99a] K. Ghosh , R. K. Rit , E. Ramesh , A. K. Sahoo , Angew. Chem. Int. Ed. 2016, 55, 7821–7825; 10.1002/anie.201600649 27008210

[99b] M. Shankar , A. Saha , S. Sau , A. Ghosh , V. Gandon , A. K. Sahoo , Chem. Sci. 2021, 12, 6393–6405; 34084439 10.1039/d1sc00765c PMC8115082

[99c] K. Ghosh , R. K. Rit , M. Shankar , K. Mukherjee , A. K. Sahoo , Chem. Rec. 2020, 20, 1017–1042. 32779389 10.1002/tcr.202000063

[100a] S.-L. Huang , D. Swern , J. Org. Chem. 1979, 44, 2510–2513;

[100b] C. Bolm , C. P. R. Hackenberger , O. Simic , M. Verrucci , D. Muller , F. Bienewald , Synthesis 2002, 879–887;

[100c] C. Bolm , H. Okamura , M. Verrucci , J. Organomet. Chem. 2003, 687, 444–450;

[100d] G. Y. Cho , C. Bolm , Org. Lett. 2005, 7, 1351–1354. 15787504 10.1021/ol050176b

[101] M. Muneeswara , S. S. Kotha , G. Sekar , Synthesis 2016, 48, 1541–1549.

[102] B. D. Bala , N. Sharma , G. Sekar , RSC Adv. 2016, 6, 97152–97159.

[103] N. Sharma , G. Sekar , RSC Adv. 2016, 6, 37226–37235.

[104] A. Porey , S. Santra , J. Guin , Asian J. Org. Chem. 2016, 5, 870–873.

[105] K. K. Rajbongshi , S. Ambala , T. Govender , H. G. Kruger , P. I. Arvidsson , T. Naicker , Synthesis 2020, 52, 1279–1286.

[106] W. Jiang , Y. Huang , L. Zhou , Q. Zeng , Sci. China Chem. 2019, 62, 1213–1220.

[107] S.-r. Guo , P. S. Kumar , Y.-q. Yuan , M.-h. Yang , Tetrahedron Lett. 2017, 58, 2681–2684.

[108] S. Baranwal , S. Gupta , S. Sabiah , J. Kandasamy , Asian J. Org. Chem. 2019, 8, 2218–2227.

[109] H. Noda , Y. Asada , M. Shibasaki , N. Kumagai , Chem. Commun. 2017, 53, 7447–7450. 10.1039/c7cc03386a 28569297

[110] C. Pimpasri , L. Sumunnee , S. Yotphan , Org. Biomol. Chem. 2017, 15, 4320–4327. 28470272 10.1039/c7ob00776k

[111] C. Wang , H. Wang , C. Bolm , Adv. Synth. Catal. 2020, 3, 747–750.

[112] C. Wang , D. Ma , Y. Tu , C. Bolm , Org. Lett. 2020, 22, 8937–8940. 33124424 10.1021/acs.orglett.0c03338

[113] H. Zhou , J. Hong , J. Huang , Z. Chen , Asian J. Org. Chem. 2017, 6, 817–820.

[114] Z. Li , M. Frings , H. Yu , C. Bolm , Org. Lett. 2019, 21, 9, 3119–3122. 10.1021/acs.orglett.9b00772 30986071

[115] D. L. Priebbenow , C. Bolm , Org. Lett. 2014, 16, 1650–1652. 24588424 10.1021/ol5003016

[116] C. Bohnen , C. Bolm , Org. Lett. 2015, 17, 3011–3013. 26029817 10.1021/acs.orglett.5b01384

[117] A. Zupanc , M. Jereb , J. Org. Chem. 2021, 86, 5991–6000. 33764766 10.1021/acs.joc.1c00292 PMC8154609

[118] H. Wang , Y. Cheng , P. Becker , G. Raabe , C. Bolm , Angew. Chem. Int. Ed. 2016, 55, 12655–12658; 10.1002/anie.201605743 27444808

[119] H. Wang , D. Zhang , H. Sheng , C. Bolm , J. Org. Chem. 2017, 82, 22, 11854–11858. 10.1021/acs.joc.7b01535 28745886

[120] J. Kalim , T. Duhail , T.-N. Le , N. Vanthuyne , E. Anselmi , A. Togni , E. Magnier , Chem. Sci. 2019, 10, 10516–10523. 32110339 10.1039/c9sc04289j PMC7020794

[121] T. Wirth , M. Elsherbini , A. Osi , H. Alharbi , F. Karam , Synthesis 2021, 10.1055/a-1508--9593.

[122] C. Wang , Y. Tu , D. Ma , C. Bolm , Angew. Chem. Int. Ed. 2020, 59, 14134–14137; 10.1002/anie.202005844 PMC7496861 32415689

[123a] C. Bolm , J. P. Hildebrand , Tetrahedron Lett. 1998, 39, 5731–5734;

[123b] P.-F. Larsson , A. Correa , M. Carril , P.-O. Norrby , C. Bolm , Angew. Chem. Int. Ed. 2009, 48, 5691–5693; 10.1002/anie.200902236 19554589

[124] C. Bolm , J. P. Hildebrand , J. Rudolph , Synthesis 2000, 7, 911–913.

[125a] C. Moessner , C. Bolm , Org. Lett. 2005, 7, 2667–2669; 15957917 10.1021/ol050816a

[125b] R. K. Chinnagolla , A. Vijeta , M. Jeganmohan , Chem. Commun. 2015, 51, 12992–12995. 10.1039/c5cc04589d 26177516

[126] J. Kim , J. Ok , S. Kim , W. Choi , P. H. Lee , Org. Lett. 2014, 16, 4602–4605. 25142135 10.1021/ol502174n

[127] B. Vaddula , J. Leazer , R. S. Varma , Adv. Synth. Catal. 2012, 354, 986–990.

[128] S. K. Aithagani , S. Dara , G. Munagala , H. Aruri , M. Yadav , S. Sharma , R. A. Vishwakarma , P. P. Singh , Org. Lett. 2015, 17, 5547–5549. 26562479 10.1021/acs.orglett.5b02804

[129] Y. Jiang , Y. You , W. Dong , Z. Peng , Y. Zhang , D. An , J. Org. Chem. 2017, 82, 5810–5818. 28508647 10.1021/acs.joc.7b00633

[130] A. Wimmer , B. Königa , Adv. Synth. Catal. 2018, 360, 3277–3285. 30344467 10.1002/adsc.201800607 PMC6175368

[131] Q. Yang , P. Y. Choy , Q. Zhao , M. P. Leung , H. S. Chan , C. M. So , W.-T. Wong , F. Y. Kwong , J. Org. Chem. 2018, 83, 11369–11376. 30062889 10.1021/acs.joc.8b01599

[132] W. Dong , C. Liu , X. Ma , Y. Zhang , Z. Peng , D. Xie , D. An , Tetrahedron 2019, 75, 3886–3893.

[133] A. Wimmer , B. König , Org. Lett. 2019, 21, 2740–2744. 30938532 10.1021/acs.orglett.9b00698 PMC6480096

[134] D. Liu , Z. Liu , C. Ma , K. Jiao , B. Sun , L. Wei , J. Lefranc , S. Herbert , T. Mei , Angew. Chem. Int. Ed. 2021, 60, 9444–9449. 10.1002/anie.202016310 33576561

[135] S. K. Aithagani , M. Kumar , M. Yadav , R. A. Vishwakarma , P. P. Singh , J. Org. Chem. 2016, 81, 5886–5894. 27304317 10.1021/acs.joc.6b00593

[136a] A. T. Londregan , K. Burford , E. L. Conn , K. D. Hesp , Org. Lett. 2014, 16, 3336–3339; 24885646 10.1021/ol501359r

[136b] A. T. Londregan , S. Jennings , L. Wei , Org. Lett. 2011, 13, 1840–1843. 21375291 10.1021/ol200352g

[137] L. Sumunnee , C. Pimpasri , M. Noikham , S. Yotphan , Org. Biomol. Chem. 2018, 16, 2697–2704. 29582873 10.1039/c8ob00375k

[138a] C. Enguehard-Gueiffier , A. Gueiffier , Mini-Rev. Med. Chem. 2007, 7, 888–899; 17897079 10.2174/138955707781662645

[138b] J. Liu , Q. Chen , Prog. Chem. 2010, 22, 631–638.

[139] N. Luan , Z. Liu , S. Han , L. Shen , J. Li , D. Zou , Y. Wu , Y. Wu , Tetrahedron 2020, 61, 151362–151363.

[140] J. Xu , Q. Song , Org. Chem. Front. 2017, 4, 938–942.

[141] L. Wang , D. L. Priebbenow , X. Y. Chen , F.-F. Pan , C. Bolm , Eur. J. Org. Chem. 2015, 2015, 3338–3343.

[142] D. Zhang , H. Wang , H. Cheng , J. G. Hernández , C. Bolm , Adv. Synth. Catal. 2017, 359, 4274–4277.

[143] H. Wang , M. Frings , C. Bolm , Org. Lett. 2016, 18, 2431–2434. 27168417 10.1021/acs.orglett.6b00958

[144a] D. Saha , G. Jain , A. Sharma , RSC Adv. 2015, 5, 70619–70639;

[144b] A. K. Ganguly , S. S. Alluri , D. Caroccia , D. Biswas , C.-H. Wang , E. Kang , Y. Zhang , A. T. McPhail , S. S. Carroll , C. Burlein , V. Munshi , P. Orth , C. Strickland , J. Med. Chem. 2011, 54, 7176–7183. 21916489 10.1021/jm200778q

[145] J. Wen , H. Cheng , G. Raabe , C. Bolm , Org. Lett. 2017, 19, 6020–6023. 29045158 10.1021/acs.orglett.7b03106

[146] J. Huang , Y. Huang , T. Wang , Q. Huang , Z. Wang , Z. Chen , Org. Lett. 2017, 19, 1128–1131. 28212044 10.1021/acs.orglett.7b00120

[147] P. Shi , Y. Tu , C. Wang , D. Kong , D. Ma , C. Bolm , Org. Lett. 2020, 22, 8842–8845. 33170727 10.1021/acs.orglett.0c03212

[148] Y. Li , L. Dong , Org. Biomol. Chem. 2017, 15, 9983–9986. 29170775 10.1039/c7ob02586f

[149] Y. Sun , N. Cramer , Angew. Chem. Int. Ed. 2018, 57, 15539–15543; 10.1002/anie.201810887 30300950

[150] M. Brauns , N. Cramer , Angew. Chem. Int. Ed. 2019, 58, 8902–8906; 10.1002/anie.201904543 31045299

[151] T. Zhou , P.-F. Qian , J.-Y. Li , Y.-B. Zhou , H.-C. Li , H.-Y. Chen , B.-F. Shi , J. Am. Chem. Soc. 2021, 143, 6810–6816. 33909436 10.1021/jacs.1c03111

[152] H. Zhou , W. Chen , Z. Chen , Org. Lett. 2018, 20, 2590–2594. 29664649 10.1021/acs.orglett.8b00776

[153] X. Chen , H. Zhou , Z. Chen , Org. Chem. Front. 2019, 6, 3415–3419.

[154] H.-B. Xu , J.-H. Yang , X.-Y. Chai , Y.-Y. Zhu , L. Dong , Org. Lett. 2020, 22, 2060–2063. 32101014 10.1021/acs.orglett.0c00520

[155] B. Wang , X. Han , J. Li , C. Li , H. Liu , Molecules 2020, 25, 2515. 10.3390/molecules25112515 PMC7321204 32481606

[156] Y. Hua , W. Zhang , X. Wang , Z. Ge , R. Li , Tetrahedron 2017, 73, 4387–4391.

[157] E. Boulard , V. Zibulski , L. Oertel , P. Lienau , M. Schäfer , U. Ganzer , U. Lücking , Chem. Eur. J. 2020, 26, 4378–4388. 31961028 10.1002/chem.201905461

