# Supplementary material for: Synthesis and Transformations of NH‐Sulfoximines
Source: Chemistry. 2021 Oct 13;27(69):17293–321. doi: 10.1002/chem.202102619 (PMC9291533; doi:10.1002/chem.202102619)
Supplement: Supplementary file 1 — Supporting Information [file CHEM-27-17293-s001.pdf]

Author Contributions

M.A. Writing – original draft:Equal
A.T. Writing – original draft:Equal
L.D. Writing – review & editing:Supporting
J.B. Writing – review & editing:Lead
R.L. Writing – original draft:Lead; Writing – review & editing:Equal
